# Discovering new potential inhibitors to SARS-CoV-2 RNA dependent RNA polymerase (RdRp) using high throughput virtual screening and molecular dynamics simulations

**DOI:** 10.1038/s41598-022-24695-4

**Published:** 2022-11-21

**Authors:** Dylan Brunt, Phillip M. Lakernick, Chun Wu

**Affiliations:** College of Science and Mathematics, Rowan University, Glassboro, NJ 08028 USA

**Keywords:** Computational biology and bioinformatics, Drug discovery

## Abstract

RNA dependent RNA polymerase (RdRp), is an essential in the RNA replication within the life cycle of the severely acute respiratory coronavirus-2 (SARS-CoV-2), causing the deadly respiratory induced sickness COVID-19. Remdesivir is a prodrug that has seen some success in inhibiting this enzyme, however there is still the pressing need for effective alternatives. In this study, we present the discovery of four non-nucleoside small molecules that bind favorably to SARS-CoV-2 RdRp over the active form of the popular drug remdesivir (RTP) and adenosine triphosphate (ATP) by utilizing high-throughput virtual screening (HTVS) against the vast ZINC compound database coupled with extensive molecular dynamics (MD) simulations. After post-trajectory analysis, we found that the simulations of complexes containing both ATP and RTP remained stable for the duration of their trajectories. Additionally, it was revealed that the phosphate tail of RTP was stabilized by both the positive amino acid pocket and magnesium ions near the entry channel of RdRp which includes residues K551, R553, R555 and K621. It was also found that residues D623, D760, and N691 further stabilized the ribose portion of RTP with U10 on the template RNA strand forming hydrogen pairs with the adenosine motif. Using these models of RdRp, we employed them to screen the ZINC database of ~ 17 million molecules. Using docking and drug properties scoring, we narrowed down our selection to fourteen candidates. These were subjected to 200 ns simulations each underwent free energy calculations. We identified four hit compounds from the ZINC database that have similar binding poses to RTP while possessing lower overall binding free energies, with ZINC097971592 having a binding free energy two times lower than RTP.

## Introduction

The highly contagious coronavirus induced illness COVID-19 is now a global threat to human well-being, with the current case numbers rising near 220 million and over 4.5 million deaths as of September 2021. With the increase in rollouts of vaccinations around the world, there are now some signs of relief to even the most affected regions. However, there is still the potential for new vaccine resistant strains of the coronavirus are evolving often as seen in the recent deadly outbreak of the new B.1.617.2 “Delta” variant in Nepal^1^. Additionally, there is the possibility for some vaccinated individuals to not develop an adequate immune response to create neutralizing antibodies. Therefore, there is still an urgent need for effective drug therapy in order to slow down the progression of sickness in affected patients. The SARS-CoV-2 virus is a positive-strand RNA virus whose genomic structure expresses high similarity with previous severe acute respiratory syndrome coronavirus (SARS-CoV). Like other RNA viruses, the replication of SARS-CoV-2 requires RNA-dependent RNA polymerase (nsp 12), a key enzyme which regulates the viral genome replication and transcription within the viral life cycle. It is thus a key validated target for the development of COVID-19 disease small molecule therapeutics.

Currently, Gilead’s Remdesivir (RDV) otherwise known as Veklury^Ⓡ^ has been approved by the FDA as an antiviral prodrug which targets the nucleotide addition process in RdRp. This prodrug is bio transformed in the body into the active-form remdesivir-triphosphate (RTP), a nucleoside mimic to adenosine triphosphate (ATP), which then undergoes nucleotide addition onto the growing RNA chain^2^*.* It is theorized that RDV inhibits RdRp by chain termination during the RNA translocation step^3^. Its action on treating SARS-CoV-2 infection has seen some success in reducing the time needed for recovery in very sick patients that require hospitalization^4^. However, this drug’s efficacy is also controversial, as its administration was recently halted due to the lack of significant improvement expected in COVID-19 infected patients. Therefore, because RDV is the only FDA approved drug for treating COVID-19 patients, there is interest in the discovery of alternative treatments. Additionally, there is interest in developing non-nucleoside mimetics with high RdRp affinity for this purpose, as these are thought to circumvent the polymerization reaction*.*

**Figure 1:**
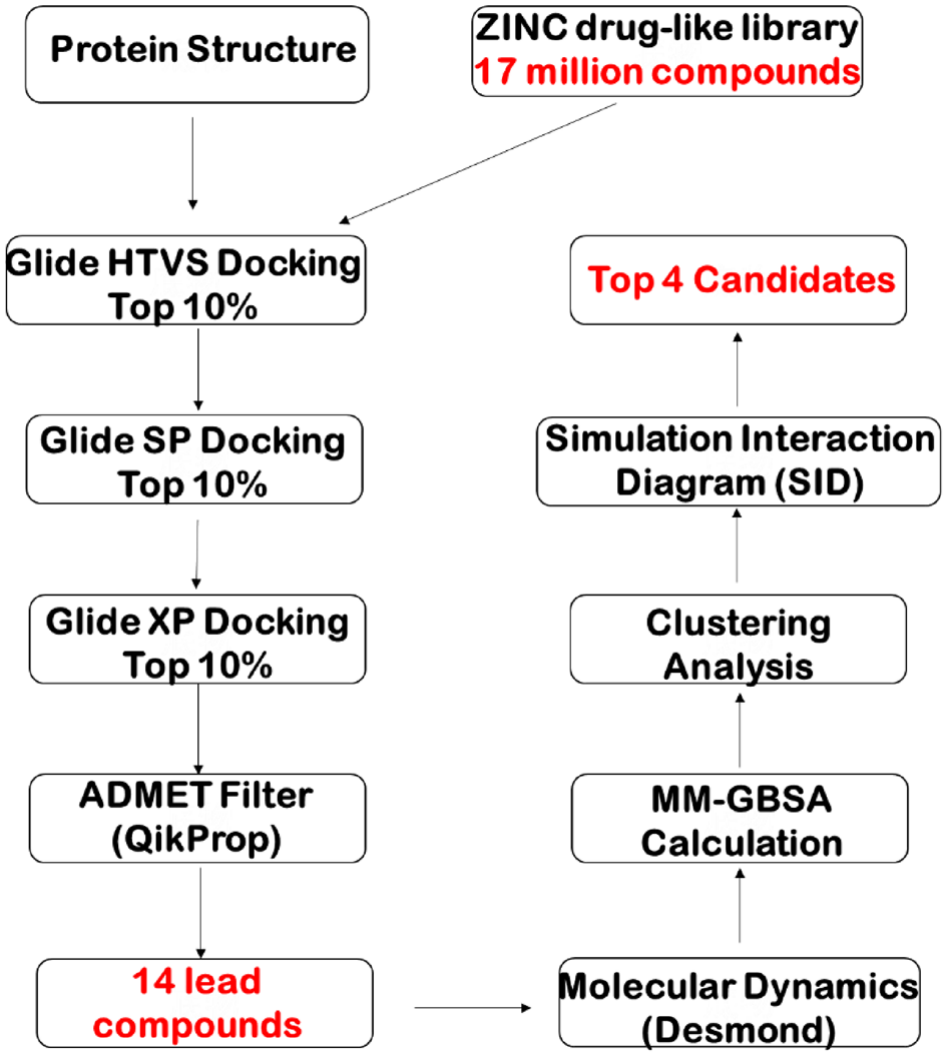
Virtual screening workflow designed to conduct this study.

There is a remarkable effort underway from researchers around the globe to help shed more light on potential alternatives for the inhibition of RdRp. There have been several studies (Table 1)^3,5–42^ that have reported the discovery of potential small molecule inhibitors of RdRp, mostly deriving from the in-silico approach coupled with virtual drug screening of numerous compound libraries. As of this study, few in-silico studies have considered the approach of using the massive ZINC library of approximately 17 million compounds using high-throughput virtual screening workflow (Fig. 1) paired with long, multi-system molecular dynamics (MD) simulations^43^. A previous study reported the discovery of two potential isoformic RdRp inhibitors: ZINC09128258 and ZINC09883305, however these were observed to have lower docked binding affinity than the compared standards^21^.

**Table 1:** Survey of the current studies on the discovery and/or repurposing of approved drugs for the inhibition of SARs-COV-2 RdRp.

Method	Protein target	Ligand library	# Output	Author
Extension reactions	None	6 FDA approved antiviral agents	6	Chien et al.^4^
Extension reactions, exonuclease reactions	None	Sofosbuvir	1	Jockusch et al.^2^
Extension reactions	None	Oligonucleotides purchased from DNA Technologies	3	Ju et al.^5^
Therapeutics	6NUR	10 Antiviral drugs	2	Aftab et al.^6^
Virtual screening	6M71	FDA Approved database of 7922 molecules	7	Ahmad et al.^7^
Virtual screening	6NUR, 6NUS	11 Favipiravir analogues	1	Aktas et al.^8^
Docking, ADMET analysis, bioactivity prediction	6M17	113 Quinoline-drugs	5	Alexpandi et al.^9^
Docking	6NUR	5 Pharmaceutical drugs	5	Al-Masoudi et al.^10^
Systematic screening, bioassays	6NUR	4947 Drugs from DrugBank, ChEMBL, Binding Database	102	Ao et al.^11^
Docking	5B6O	16 Antiviral drugs	4	Calligari et al.^12^
Docking	6M71	7 Falvonoid drugs	2	da Silva et al.^14^
Docking	6M71	171 Essential oil components	3	da Silva et al.^13^
Sequence analysis, docking, modeling	6NUR	8 Pharmaceutical drugs	4	Elfiky^15^
Virtual screening	6M71, 7BTF	65 FDA approved small molecule antiviral drugs	5	Indu et al.^17^
Comprehensive analysis	7BV2	22 FDA approved drugs	2	Kandeel et al.^18^
Docking	Crystal Structure	Carotane sesquiterpenes	1	Mohamed et al.^19^
Docking	6M71, 7BV2	44 Drug candidates	5	Parvez et al.^20^
Virtual screening	6M71, 6NUR	FDA approved drugs	1	Pokhrel et al.^21^
Docking, free energy calculations	6NUR	8 FDA approved drugs	8	Ruan et al.^22^
Modeling	6YYT	2924 Compounds from the approved drug database	1	Tchesnokov et al.^23^
Modeling, Docking	Homology Model	FDA Approved drug database	135	Wu et al.^24^
Virtual screening	384 PDB Structures	7894 Drug data compounds	10	Zhao et al.^25^
Docking, MD simulation (100 ns)	6NUR, 6M71, 7BTF	76 Perscription drugs	4	Ahmed et al.^26^
MD simulation (30 ns)	6NUR	3277 Approved drugs	3	Barage et al.^27^
Docking, MD simulation (3.99 ns)	6M71	13 Alkaloids from *Cryptolepis sanguinolenta*	13	Borquaye et al.^28^
MD simulation (100 ns), docking	7BTF, 6M17	29 Bioactive compounds from South African medicinal plants	4	Dwarka et al.^29^
Docking, MD simulation (150 ns)	7BV2	MAW-22 (FBDD of the top 5 fragments)	1	El Hassab et al.^30^
Modeling, docking, MD simulation (51 ns)	6NUR, 7BTF, 6M71	30 Drugs against the SARS-CoV-2 RdRp	15	Elfiky^16^
MD simulation (260 ns), modeling, docking	2XI3	7 Pharmaceutical drugs	4	Elfiky et al.^15^
MD simulation (50 ns)	6M71	8 Pharmaceutical drugs	8	Elkarhat et al.^41^
MD simulation (20 ns), docking	6NUR	A select number of pharmaceutical drugs from the FDA	18	Gul et al.^31^
MD simulation (5.5 ns), docking	7BV2	97 Natural amide-like compounds	3	Gutierrez-Villagomez et al.^32^
Docking, MD simulation (40 ns)	7BV2	12 Species of Clerodendrum	1	Kar et al.^33^
Docking, MD simulation (100 ns)	6M71	617 Source species from Northern South Africa	5	Khan et al.^34^
MD simulation (100 ns)	6M71	FDA approved and investigational drug	2	Mutlu et al.^35^
MD simulation (50 ns)	6M71, 7BV2	Various libraries of FDA approved drugs, natural products, antiviral compounds, and drug repurposing compounds	1	Narayanan et al.^36^
Docking, MD simulation (10 μs)	6M71, 6NUR	8800 Drug structures obtained from DrugBank	10	Ribaudo et al.^37^
Docking, MD simluation (50 ns)	6M71	22 Major bioactive molecules from 10 medicinal plants	7	Sharma et al.^38^
Docking, MD simulation (50 ns)	6NUR	12 FDA approved drugs 6	6	Singh et al.^39^

In our present study, we have utilized long MD simulations (1.0 µs) each of SARS-CoV-2 RdRp in complex with free form active RTP and ATP and template-primer RNA starting from the recently solved crystal complex (PDBID: 7BV2)^44^. Using the most abundant conformations of RTP complex from 1.0 µs simulations, we performed pharmacophore-based screening of the ZINC 15 library against the binding site of RdRp, leading to fourteen compounds with top docking scores^43^. From these top fourteen compounds, we performed classical MD simulations (200 ns each) to observe the stability for each complex system. Lastly, we performed Molecular Mechanism-General Born Surface Area (MM-GBSA) energy predictions to observe their relative binding affinities to RdRp after simulation^39,45–47^. Four ZINC compounds showed stable binding to the RdRp-RNA complex. Additionally, these four systems showed improved MM-GBSA energies both the RTP and ATP complex systems as well as in reference to their initial docked complexes. This discovery helps introduce crucial knowledge of small molecule binding to RdRp in addition to the effort in discovering and development of anti-SARS-CoV-2 RdRp agents using in-silico methods.

## Experimental section

### Structure building and ligand preparation

The recent electron microscopy solved crystal structure of SARS-COV-2 nsp12-nsp7-nsp8 RdRp in complex with template-primer RNA and covalently bound RDV named PDB ID 7BV2 was retrieved from the RCSB protein database^44,48^. This structure was then prepared with the default charge state being optimized at pH 7 using Maestro’s built-in Protein Preparation Wizard^49–51^. The complex was then preprocessed, optimized, and minimized using the Protein Preparation Wizard within Maestro software^49^. A receptor docking grid was generated around the covalently bound RDV with a X: 7.07, Y: − 3.87 and Z: 1.27 spatial coordinates and a grid box size allowing for ligands with length ≤ 20 Å. Free form RTP was then generated by breaking phosphodiester bonds between U20 of primer strand and remdesivir. The phosphate tail was then added onto the 5′ carbon of remdesivir to render the triphosphate form bound within the *i* binding site. The ligand RTP was then redocked using the receptor grid file generated prior. The resulting docking grid was also applied for the docking of ATP towards RdRp. Before any docking procedures, the ligands were first prepared for docking by generating the ionization/tautomeric states at pH 7 ± 2 using Maestro’s Epik tool^52,53^. Each ligand was docked utilizing the Glide module within Maestro software and the GlideXP scoring function was employed^50,51,54^.

### MD system setup

The OPLS2005 force field was used to model the protein and the ligands^55–57^. When creating a MD simulation system for RDV-TP, ATP and the 14 ZINC compounds, its XP docked complex with the two crystal Mg^2+^ ions was placed into an orthorhombic water box with a buffer distance of 10 Å using the SPC water model^58^ and a salt concentration of 0.15 M NaCl was added to the system^55,56^.

### Relaxation and production runs

The MD simulations for each system were performed using the Desmond simulation package^59^ and the default simulation protocols were followed just like our early work^60–62^. Here a brief description is given. Each system was relaxed using the default relaxation protocol for non-membrane proteins^63^. There are eight stages which constitute the protocol: (1) minimization of the system with a restraint of solute heavy atoms. (2) Minimization without any restraints. (3) Simulation with heating from 0 to 300 K, H2O barrier and gradual restraining. (4) Simulation under the NPT ensemble which is constant number of particles, pressure of 1 bar and constant temperature of 300 K with an H2O barrier and heavy atoms being restrained. (5) Simulation under the NPT ensemble with equilibration solvent. (6) Simulation under the NPT ensemble with protein heavy atoms annealing from 10.0 to 2.0 kcal/mol. (7) Simulation under the NPT ensemble with Cα atoms restrained at 2.0 kcal/mol; and (8) simulation of 1.5 ns under the NPT ensemble with no restraints. After this relaxation procedure, six 1000 ns production runs for RTP, ATP and four top zinc ligand systems, and ten 200 ns production runs were conducted under the NPT ensemble using the default protocol, leading to a total of 8000 ns. M-SHAKE^64^ was used to constrain bonds with hydrogen atoms, which allowed for 2.0 fs time steps during the simulation. Long-range electrostatic interactions were treated under periodic boundary conditions by the k-space Gaussian split Ewald method^65^. The charge grid spacing was ~ 1 Å and the direct sum tolerance was 10^–9^. Van der Waals interactions were based on a uniform density approximation. A distance of 9 Å was set as the cutoff for short range non-bonded interactions. Non-bonded forces were calculated by an r-REPA integrator^66^; the short range forces updated every 2 fs and the long range every 6 fs. The trajectories were saved every 50 ps for analysis. Pressure of 1 bar was controlled using the Martyna–Tobias–Klein chain coupling scheme^67^ (coupling constant = 2 ps), and temperature of 300 K by the Nosé–Hoover^67^ chain coupling scheme (coupling constant = 1 ps).

### Conformational clustering of RdRp complexes

The Desmond trajectory clustering tool was used to group the complex structures from the last 100 ns of simulation for the ATP and RTP systems^58^. The merging distance cutoff was set to be 2.5 Å. The centroid representative (i.e. the structure having the largest number of neighbors in the structural family) was used to display the structural family. The most abundant conformations are identified as the cluster with the most occupancy. This was performed for the complexes containing ATP and RTP. Clustering was then performed on the top fourteen ZINC compounds selected from HTVS after 200 ns simulation.

### Pharmacophore screening and hit prioritization

The pharmacophore screening was performed using the active Remdesivir against ZINC product molecules using LigandScout 4.3^68^. In our model, we determined the seven pharmacophore features to include the two negative ionizable groups, one aromatic ring, and four hydrogen bonds acceptors with their corresponding positions. Before further experiments, we determined the ionizable/tautomeric states of each compound under pH 7 ± 2 using Maestro’s Epik tool^52,53^. The lowest ionization/tautomeric states were selected, and their bond geometries were minimized to the lowest energetic state. We then employed our prepared RdRp complex to generate a receptor grid file around the RTP binding site, or *i* site, and the ionization, tautomeric and bond states of ATP were generated at pH ± 2 using Maestro’s Epik tool^52,53^. This receptor grid file was additionally used for our docking of the ZINC database compounds using the Glide XP docking scoring function within Maestro software^49,68^. High-Throughput Virtual Screening (HTVS) was also performed using the Glide module on the most abundant cluster representative of the RTP system after simulation utilizing the ZINC15 compound database^43,50,51^. This process consists of screening at three levels with gradually increasing the computational cost. The first level is based on ligand ADME/Toxicity prediction using QikProp of Schrodinger and docking using Glide^69^. The second is performed using MD simulations of the docked complexes with explicit solvents (water)^63^. Lastly, the third is determined by the binding energy calculations. After HTVS, we narrowed down the library of compounds to the top fourteen based on their superior Glide scores and #star value^50,51^. We then subjected these fourteen to 200 ns simulations and performed MM-GBSA calculations to determine their relative binding affinities after the simulation^39,45^. From the fourteen compounds, four compounds were selected based on their favorable binding energies.

### Confirmation of stability of hits and MM-GBSA calculation

The Desmond SID module was employed to analyze the interaction between proteins and ligands as well as to determine their stability in each MD simulation^63^. This includes the Root-Mean Square Deviation (RMSD) of the protein–ligand complexes and Root-Mean Square Deviation (RMSF) of both the protein and ligands. Additionally, secondary structure elements (SSE) were explored. Lastly, the protein–ligand contacts containing H-bond, ionic, hydrophobic interactions were explored.

In order to check the convergence of the simulations, we investigated the root mean square deviation (RMSD) values of protein Cɑ, RNA O5′ atoms, and ligand main atoms for each trajectory using the SID tool within Maestro software^63^. The root mean square fluctuation (RMSF) of each individual amino acid, RNA and ligand atom were also calculated to characterize the local movement of individual structural components of each complex.

The Molecular Mechanism-General Born Surface Area (MM-GBSA) binding energies were calculated on the frames for the whole duration of both systems as well as for each ZINC system^45^. The OPLS3 force field, VSGB 2.0 solvation model and the default Prime procedure was used for the MM-GBSA calculations^46,47,55–57,70^. The default procedure consists of three steps: Receptor alone (minimization), Ligand alone (minimization), Receptor-ligand complex (minimization). The total binding free energy equation is: ΔG_Bind_ = E_complex (minimized)_ − (E_ligand (minimized)_ + E_receptor(minimized)_). To gain a more detailed understanding of binding nature, the original interaction terms (Coulombic + H-bond + GB solvation + van der Waals + π–π packing + self-contact + lipophilic) were merged into three components: E_electrostatics_, E_vdW_, and E_lipophilic_, where E_electrostatics_ = (H_bond_ + E_coulomb_ + E_GBsolvation_), E_vdW_ = (E_vdW_ + E_π-π_ + E_self-contact_) and E_lipophilic_.

### Normal mode analysis by PCA

The trajectory of ATP, RTP and the four top zinc ligands was used in the Normal Mode Wizard in VMD^71^ to generate the top 5 normal modes by a principal component analysis (PCA).

## Results and discussion

### The binding of remdesivir triphosphate and ATP to RdRp

Adenosine triphosphate was docked against the crystal structure of SARS-CoV-2 RdRp (PDBID: 7BV2) at the *i-*binding site which formed hydrogen pairing with the template strand of 14 nucleotide bases and the primer strand of 11 nucleotide bases^44^. The conformational pose of the docked RTP compared to the covalently bound RDV in the crystal structure was nearly identical (Fig. S2). We then generated a docking grid from the docking pose of RTP to dock ATP into the same catalytic site of RdRp. The conformational pose of ATP compared to the covalently bound crystal RDV was nearly identical. It was also seen that the triphosphate tail of RTP was positioned near one of the Mg^+^ ions. These conformations were then used to perform long (1.0 µs) simulations for each complex to further elucidate more about the relaxed conformations of each and to obtain a stable conformation for our high-throughput virtual screening portion of the study.

**Figure 2:**
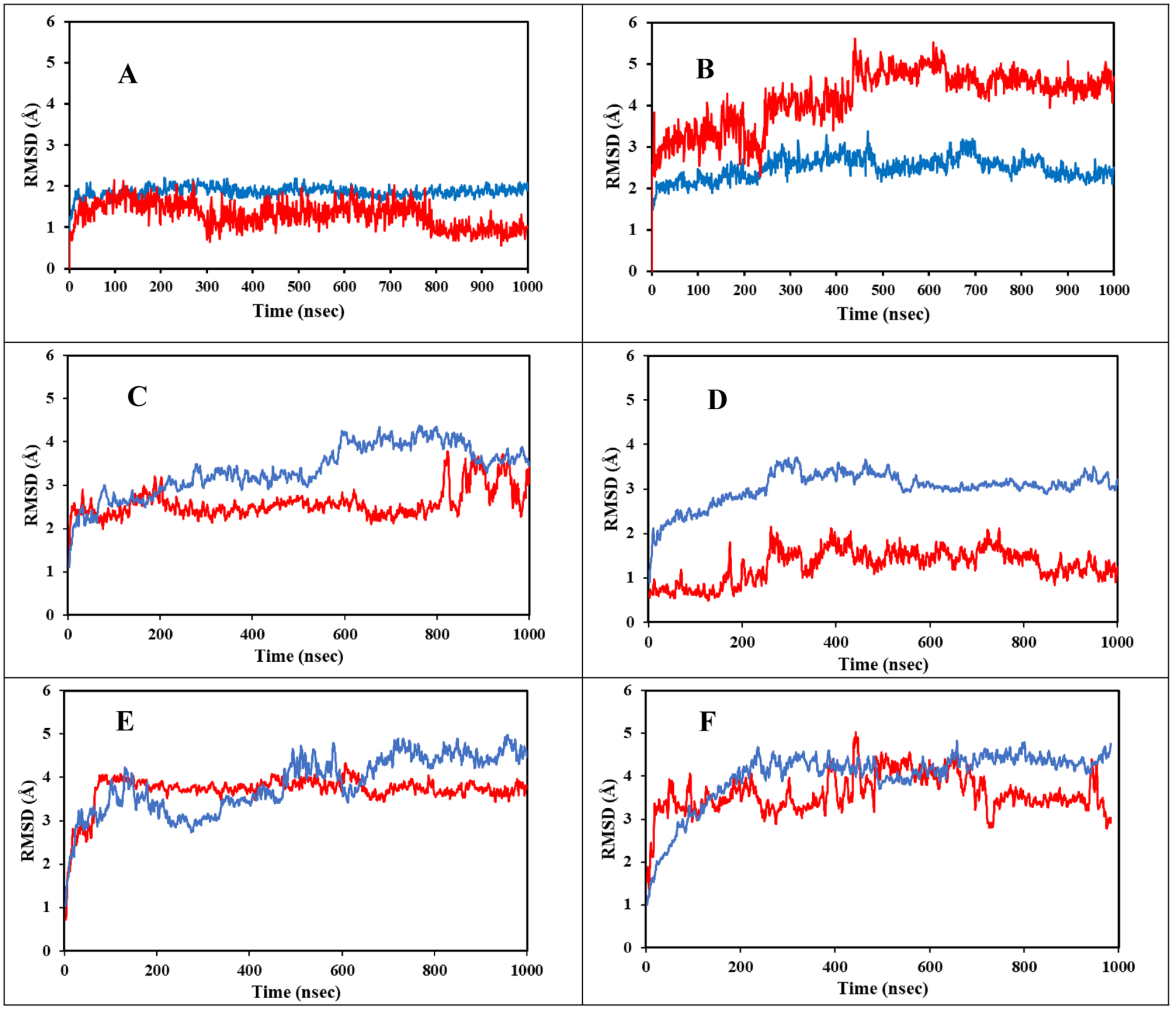
The RMSD values for the receptor Cα and ligand main atoms for the RTP, ATP and four ZINC systems. Protein Cα is shown in blue and ligand main atoms is shown in red. Figure (**A**) is the system containing RTP, (**B**) is the system containing ATP, (**C**) is the system containing ZINC097971592, (**D**) is the system containing ZINC002146610, (**E**) is the system containing ZINC069492350 and (**F**) is the system containing ZINC408592119.

The trajectory convergence of the simulation for both systems was confirmed through analyzing the RMSD during the 1000 ns (1.0 µs) simulations shown in Fig. 2A,B. The trajectory RMSD is of the protein receptor backbone Cɑ atoms and the ligand main atoms for both the ATP and RTP complexes. It is clearly seen in Fig. 2A that the RTP system reached stability very early in the simulation (~ 50 ns) while the ATP system in Fig. 2B reached stability around 550 ns. Importantly, both protein complexes maintained average RMSD values of approximately 2.0 Å. This is indicative that the differences between ATP and RTP binding had no significant change in the overall RdRp conformation through the 1.0 µs simulation.

The ligand RMSD average values for the ATP system are within 1 Å of the protein RMSD values and reached stability at approximately 550 ns. The ligand RMSD for the RTP system also matched the average distance as in the protein with a convergence very early in the simulation; approximately 50 ns. It is clearly seen that the complex containing ATP possessed higher RMSD values compared to the RTP complex indicating that the binding of RTP was more stable during the simulation.

We additionally wanted to confirm the stability of the template-primer RNA in the complex through simulation. The RMSD of the RNA main atoms is shown in Fig. S3, both ATP and RTP systems show clear stability and early convergence at around 50 ns with values both approximately 2.0 Å which is also consistent with the protein Cɑ RMSD. Likewise seen in the ligand main atoms RMSD plot, the complex containing RTP showed slightly lower average values compared to the ATP system indicating more stable binding occurred during the simulation. To observe the fluctuation of each individual amino acid in the protein structure, we recorded the RMSF of the RdRp complexes during the simulation.

Ligand main atoms RMSF (Fig. S5) was also recorded to observe the fluctuation of the individual atoms of each ligand in order to better understand the regions of stability of ATP and RTP. Clearly, RTP in complex saw less fluctuation per atom over the simulation as compared to ATP. Short peaks at atoms 1–4 indicate the slight instability seen in the gamma phosphate on the phosphate tail which is not abnormal. Interestingly, small peaks in atoms 16 and 30 indicate a slight increase in fluctuation seen at the 3′ and 4′ hydroxyl groups on the ribose ring of RTP. This specificity at only these two polar functional groups is also seen in atoms 29 and 30 of the ATP system and likely indicates positioning for nucleophilic attack during nucleotide addition.

### Clustering analysis

**Figure 3:**
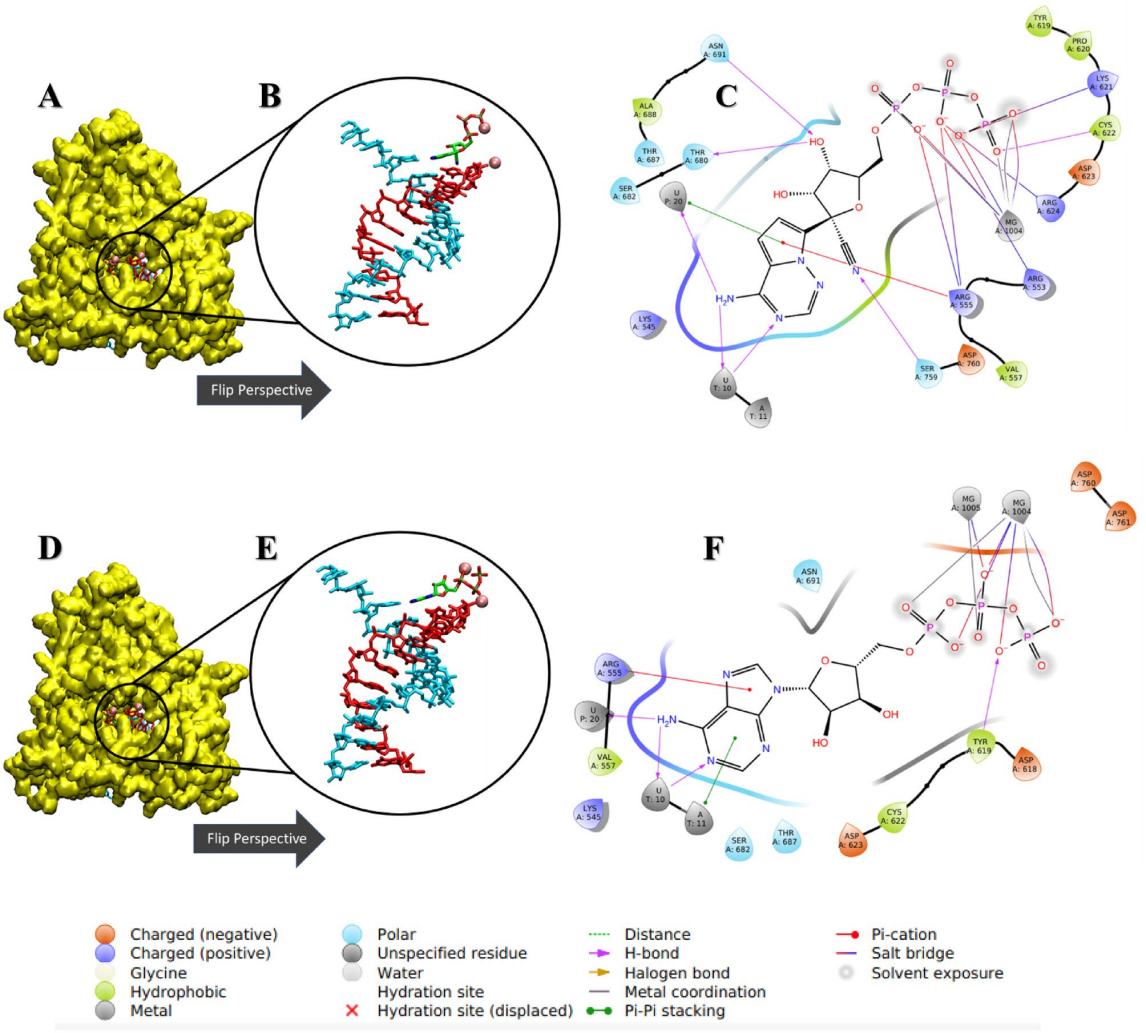
Ligand–receptor interaction diagrams and representations of the most abundant conformational pose from MD simulation. (**A,D**) The 3D surface representation of SARS-CoV-2 RdRp colored in yellow. (**B,E**) Zoomed and rotated perspective of the template-primer RNA in complex with ATP and RTP, respectively. (**C,F**) The detailed receptor-RNA-ligand contacts of the most abundant conformational pose of ATP and RTP, respectively. Template and primer strands represented in blue and red licorice, respectively. Ligands are colored in green and magnesium ions are represented as pink balls.

We then performed cluster analysis of both MD systems after the simulation to generate the most abundant conformational pose during the 1000 ns duration. In the 2D residue interaction seen with the most abundant conformational poses of each simulation (Fig. 3A–F), it was observed that the adenine motif of both ATP and RTP maintained H-Bond pairing with U10 on the template strand and partial pi-pi stacking of A11 on the primer strand. The RTP system was more able to sustain salt bridge interactions with positive residues R555, R553, R624, K621. The RTP system also saw interaction with S759 on the nitrile group while Y619 was seen to form H-Bonds with negative oxygen on the gamma phosphate of ATP. In addition, the RTP system also saw stabilization of the 3′ hydroxyl group on the ribose ring by H-Bonds with residues N691 and T680.

During the 1.0 µs simulation of both the ATP and RTP systems, several amino acid contacts were observed to be maintained throughout with high occupancy (Table S7). For both the ATP and RTP systems, there was significant occupancy in the H-Bond interactions between the triphosphate tails and a positive region of amino acids such as K551, K621, K798, R553 and R555 of ≥ 30% of the simulation time. For the RTP complex, residues K621, R553 and R555 formed H-bonds with negatively charged oxygen on alpha, beta and gamma phosphate with all possessing an occupancy of above 90%, indicating that these contacts could be crucial for the stability and positioning of RTP in the catalytic site of RdRp. These interactions were seen in previous study where both ATP and RTP were docked to RdRp^72^. Both systems also maintained the maximum occupancy interaction with Mg^+^ ions with the triphosphate tails, suggesting that at least one ion is responsible for the stabilization and positioning of incoming nucleotides. There was modest occupancy of hydrogen bond formation with hydroxyl groups located on the ribose ring portions with residues D623, D760, and N691, indicating secondary stabilizing roles for these amino acids potentially related to the initiation of nucleotide addition. Negative residue D623 saw an average occupancy of 56% bonding with both hydroxyl groups of the ribose ring portion of RTP. In the ATP complex, D760 saw 34% occupancy with the 3′ hydroxyl group located on the ribose ring which is involved in nucleotide addition.

In Table S6 are the histogram of protein–ligand contacts occupancy during the simulations for ATP and RTP complexes. These show the smaller contacts seen with occurrence of ≤ 30% of the simulation as well as contacts that occurred at over 100% which indicates multiple ligand atoms contacting same amino acid. In the ATP complex, the ligand contacted several more protein residues with brief occupancy compared to the RTP complex which is likely due to lower relative ligand bound stability seen in the former. Also, more water bridge interactions occurred with the ATP complex indicating weaker overall affinity compared to the RTP complex. Furthermore, RTP saw higher occupancy of H-bonds compared to others confirming the relative favorability of bonding to RdRp.

Previous studies have also reported similar findings on the critical amino acids involved in RDV binding. Koulgi et al. described an ensemble approach of the free form binding of prodrug RDV in complex with SARS-CoV-2 RdRp contacted amino acids Y451, T540, M542, R548, K551, R553, R555, A558, D618, S674, D761 and E811^73^. Amino acid R555 was observed to form H-bonds with the alpha phosphate group in RDV in all five ensemble representatives^73^. Other studies have aimed to observe the active triphosphate form interaction upon RdRp binding. Zhang et al. found that the nitrile group was positioned in a pocket including K545, Y546 and A547 with the phosphate tail interacting with K551, R553 and R555. Additionally, positive amino acids near the palm subdomain were found to be crucial for RTP activity^74^.

### Normal mode analysis

**Figure 4:**
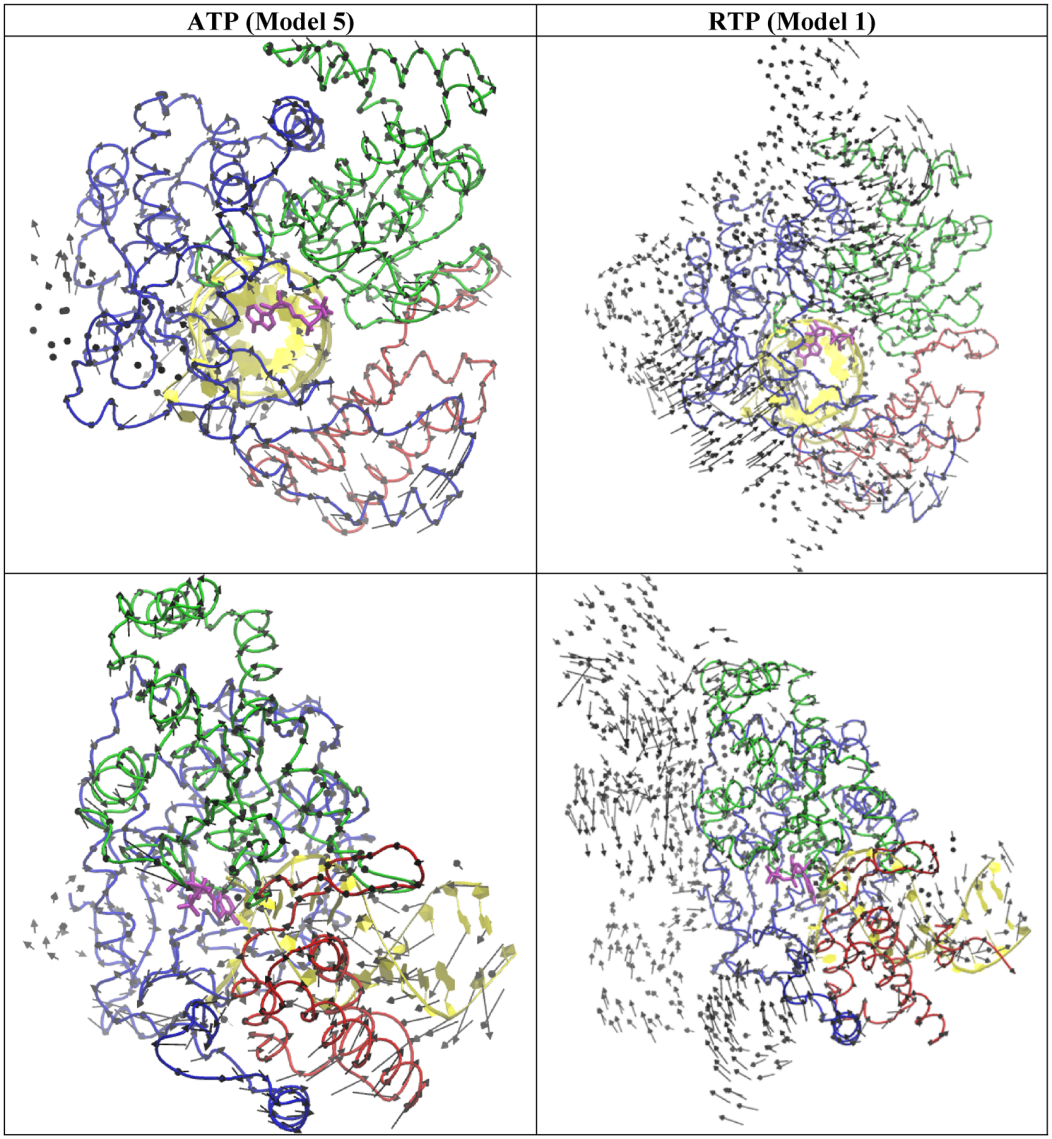
The top view (top) and the side view (bottom) of a selected normal mode from the ATP (model 5) and RTP system (model 1). RNA (yellow) and Thumb domain (Red), Palm domain (Green), and Finger domain (Blue) of RdRp is shown ribbon.

To probe the dynamics of the two systems (ATP vs RTP), the trajectory was analyzed by a PCA analysis to generate top five low frequency normal modes of ATP (Fig. S6a) and RTP (Fig. S6b). By visual inspection, although the two systems undergoes a similar opening and closing motion in their normal modes, subtle differences in magnitude between the two systems was also identified in some modes. For example, the APT system appears to have larger inter-domain movement than the RTP system among the three key RNA binding domains: Thumb domain, Palm domain and Finger domain. To show this, the mode 5 of the ATP system and the mode 1 of the RTP are shown in Fig. 4 and the movie files are provided in the supporting info. Because the ATP and RTP in these complex have already in a closed active site conformation, the translocation is the next critical step for growing the primer strand after incorporating AMP/RMP into the primer stand, this larger opening-closing mode of the ATP system than the RTP might forecast some critical difference in the translation step between the two systems. Interestingly, RTP has been found to halt RNA extension at the i + 3 site after RMP is incorporated into the RNA primer strand, through stalling the translocation of the primer strand^75^. In contrast, ATP does not have this translocation problem. In Fig. S6a, it can be observed that each mode undergoes a different opening and closing motion.

### High-throughput virtual screening finds 14 compounds

Once we validated that our simulations with ATP and RTP bound to RdRp met convergence during the simulation, we then used these complexes to perform our initial screening. Firstly, pharmacophoric screening of the ZINC database was performed by selecting seven pharmacophoric features, i.e. two negative ionizable, one aromatic ring, and four H-bond acceptors of RTP. Shown in Table 2 is the results from HTVS docking against RdRp to retrieve the top fourteen hits with the highest GlideXP scores and lowest #stars as the determining factors. The GlideXP scores measured in kcal/mol are tabulated based on decreasing favorability. A more negative GlideXP score typically indicates a good initial binding affinity to RdRp. The initial conformational pose of each ligand in complex with RdRp and RNA as well as their detailed 2D ligand contacts is shown in Table S2. These fourteen compounds were then subjected to classical 200 ns MD simulations and further MM-GBSA energy calculations for each.

**Table 2:** Summary of docking results and pharmacokinetic information of the top 14 ZINC candidates.

Molecule	Docking score (kcal/mol)	# Stars
ZINC000014651456	− 13.5	0
ZINC000257306096	− 12.9	0
ZINC000238950253	− 12.8	0
ZINC000299798705	− 12.7	0
ZINC000067790716	− 12.4	0
ZINC000089920955	− 11.9	0
ZINC000097971592	− 11.8	1
ZINC000065742965	− 11.7	0
ZINC000016040970	− 11.6	0
ZINC000408592119	− 11.6	0
ZINC000237948681	− 11.5	0
ZINC000069492350	− 11.4	0
ZINC000002146610	− 11.2	0
ZINC000084651559	− 11.2	0

Docking score is empirically calculated in kcal/mol from rigid receptor GlideHTVS protocols which help define compounds with good binding affinity. The scoring function is comprised of lipophilic, hydrogen bonding, hydrophobic terms as well as a rotable bond penalty. The #stars is a parameter defined by QikProp module and scores compounds based on their similarity to known medicines.

**Figure 5:**
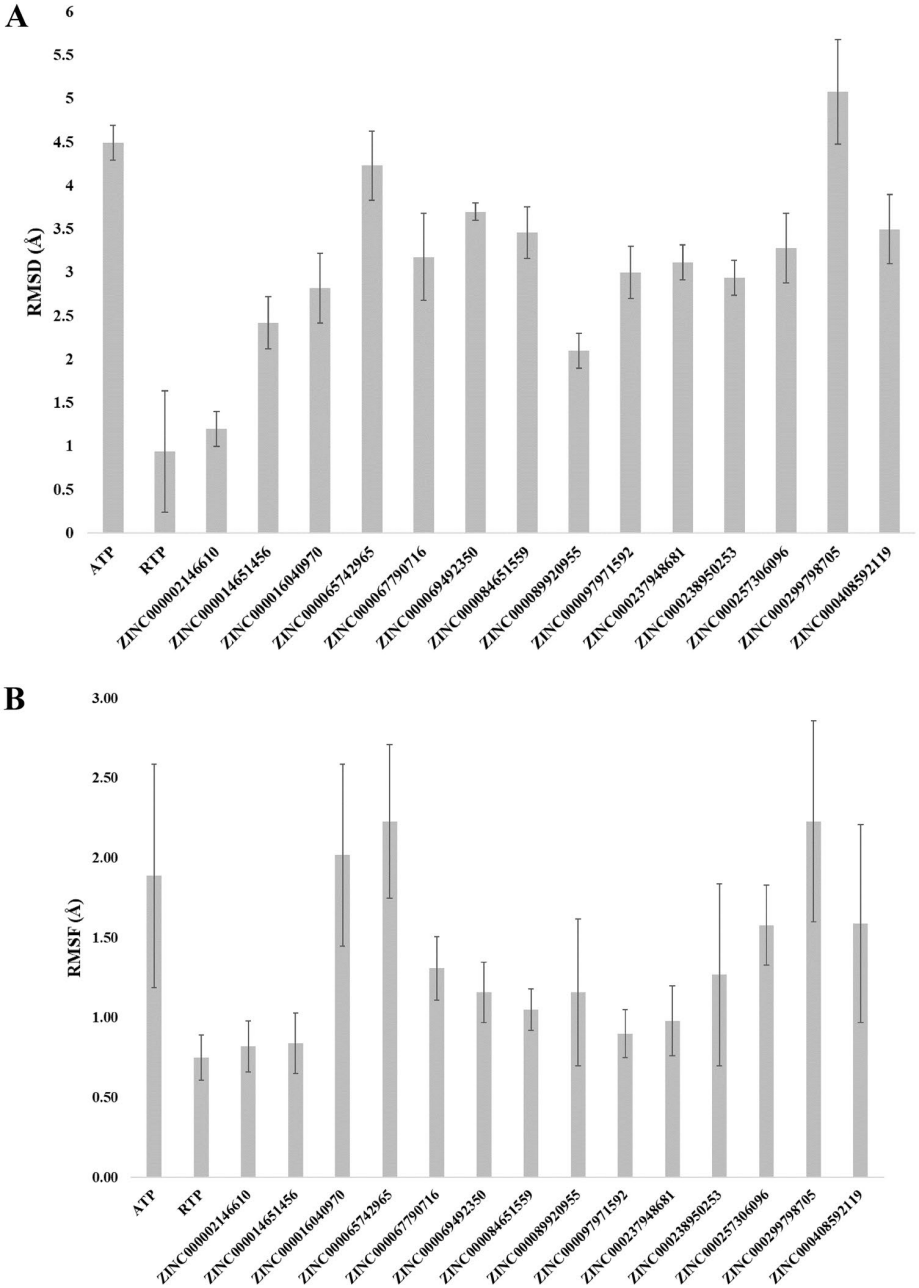
Average ligand RMSD values of all tested systems over the last 100 ns of simulation time (**A**). Average ligand RMSF of SARS-CoV-2 RdRp for all tested systems (**B**).

**Figure 6:**
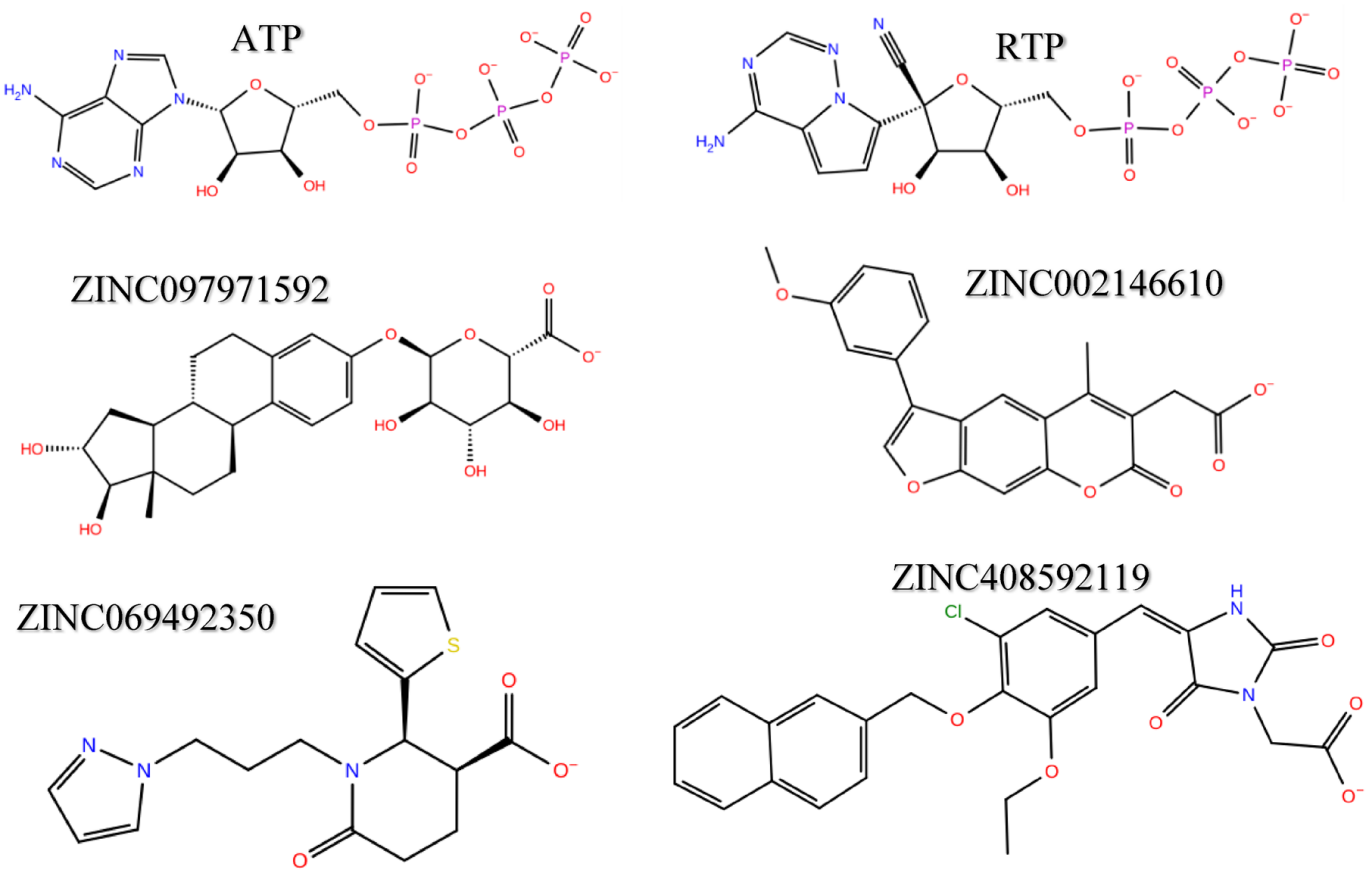
The 2D structures of RTP, ATP and the top four selected ZINC compounds.

The MM-GBSA energies and average protein–ligand RMSD of all fourteen MD simulations are summarized in Table S3. Within Fig. 5A,B show the ligand RMSD and RMSF values of all fourteen ZINC candidates with ATP and RTP systems calculated over the last 100 ns of simulation. The free energy of binding gives a more accurate insight into both the simulation efficacy and flexible binding affinity of ligands to their target as opposed to rigid ligand docking. The summary of the 200 ns simulation runs and MM-GBSA energy calculation in Table S3 shows that several compounds possessed more favorable free energy of binding compared to the ATP and RTP systems. From this subset of ligands, we selected the top four with the most favorable MM-GBSA calculated energies for further convergence analysis to ensure that the conformational stability was reached and maintained. The molecular structures of the four candidates are shown in Fig. 6. Furthermore, in Fig. 2C–F shows the RMSD of protein Cα atoms and ligand main atoms projected over the duration for these selected compounds. The protein portions reached convergence early in the simulation for each system containing ZINC097971592, ZINC002146610, ZINC069492350 and ZINC408592119. It was observed that the RMSD values of the protein Cα of the four ZINC systems were lower than that of the RTP system. Also, the ligand RMSD values for ZINC097971592 and ZINC408592119 were higher than that of the RTP complex.

**Figure 7:**
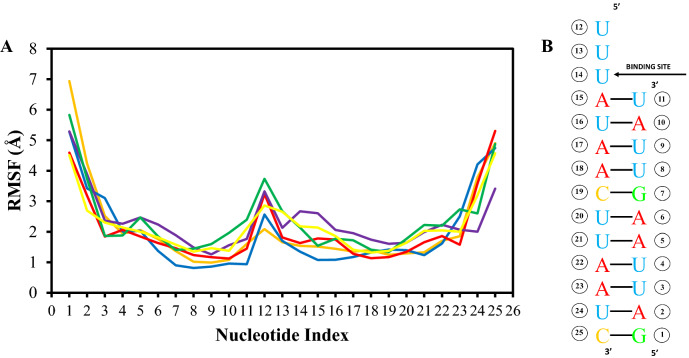
RMSF values of the template-primer RNA O5′ atoms of the ATP, RTP and four ZINC compound systems (**A**) and a guide displaying nucleotide indexing values respective to the nucleotide positions on the RNA chain (**B**).

We additionally wanted to observe the RMSF of the RNA main atoms during simulation to gain some more insight into the individual fluctuation of each nucleotide in the RNA sequence. In Fig. 7A, this displays the template-primer RNA RMSF obtained from the O5′ atoms on the RNA backbone for ATP and RTP systems and the four ZINC systems with Fig. 7B indicating the nucleotide sequence indexing used during the recording. The RMSF values were high at nucleotides G1-U3 on the 5′ end of the primer strand as well as high peaks around A22–C25 towards the 3′ end of the template strand for all 6 systems. This indicates high fluctuation induced from the exposure to solvent. In the middle of the plot is a slight peak around U12 on the template strand indicating that this portion did not interact with either ligand. Nucleotides U13-A15 on the template strand show a sharp decrease in fluctuation which is most likely due to the binding and hydrogen pairing of both ATP and RTP.

The average RMSD values over the last 50 ns of simulation for receptor and ligand is also summarized in Table 3. The four ZINC systems maintained similar average receptor RMSD values were approximately 3.9 Å which is near the value of the ATP system of 2.4 Å. This result suggests the RdRp complex system remained in similar conformational state regardless of their structure dissimilarity against ATP. Table 3 is also a breakdown of the individual MM-GBSA terms such as the Van der Waals (ΔG_VDW_), electrophilic (ΔG_ELE_) and lipophilic (ΔG_HYD_) energies of the top four selected ZINC compounds as well as ATP and RTP for comparison. The summation of these term equates to the relative free energy of binding (ΔG_Total_) measured in kcal/mol. Clearly, the four selected compounds from the ZINC database resulted in lower overall MM-GBSA values over the ATP and RTP systems of − 35.9 ± 3.1 kcal/mol and − 21.3 ± 5.9 kcal/mol, respectively. The RdRp complex containing ZINC069492350 had the most negative MM-GBSA value of − 43.8 ± 4.1 kcal/mol, with ZINC002146610, ZINC097971592 and ZINC408592119 having values of − 32.4 ± 5.1, − 37.4 ± 8.1 and − 31.4 ± 4.9 kcal/mol, respectively. A conserved trend was seen in the decomposition of each contributing energy term with the Van der Waals ΔG_VDW_ contributing the most towards the overall ΔG_Total_ especially for the case of the ZINC compounds. This is likely attributed simply to the high contract between nucleotides and the ZINC compounds in molecular mass, atom count and therefore a larger electron cloud to induce fluctuation to adjacent residues in the enzyme pocket. The average RMSD was also provided to confirm the stability off each system compared to the standard. The four ZINC systems maintained a similar average receptor RMSD value of approximately 3.3 Å compared to 2.4 Å of the ATP system. This result suggests the RdRp complex system of each ZINC system remained in a slightly more unstable conformational state against the ATP system.

**Table 3:** MM-GBSA energy values and the average receptor/ligand RMSD from the last 50 ns of 1000 ns simulation time.

Ligand	ΔG_VDW_	ΔG_ELE_	ΔG_HYD_	ΔG_Total_ (kcal/mol)	Receptor RMSD (Å)	Ligand RMSD (Å)
ATP	− 14.4 ± 5.0	− 15.3 ± 4.4	− 6.2 ± 0.7	− 35.9 ± 3.1	2.4	4.5
RTP	− 19.1 ± 4.1	6.9 ± 6.2	− 9.1 ± 1.4	− 21.3 ± 5.9	1.9	0.9
ZINC097971592	− 27.2 ± 4.4	15.2 ± 7.1	− 15.3 ± 1.7	− 37.4 ± 8.1	3.6	3.0
ZINC002146610	− 28.1 ± 3.0	12.9 ± 3.0	− 17.2 ± 1.3	− 32.4 ± 5.1	3.2	1.2
ZINC069492350	− 41.8 ± 3.1	14.3 ± 3.7	− 16.4 ± 2.1	− 43.8 ± 4.1	4.6	3.7
ZINC408592119	− 30.8 ± 3.2	23.1 ± 5.3	− 23.7 ± 1.6	− 31.4 ± 4.9	4.3	3.5

VDW energies was calculated with the summation of ligand ΔG_vdW_ energies, pi–pi packing correction energies and the self-contact correction energies.

Electrophilic terms are calculated by the summation of coulombic, hydrogen-bonding and generalized born electrostatic solvation energies. Hydrophilic term comprised of lipophilic energies. Total MM-GBSA (ΔG_Total_) calculated in kcal/mol is summation of ΔG_vdW_, ΔG_ele_ and ΔG_hyd_ terms.

**Figure 8:**
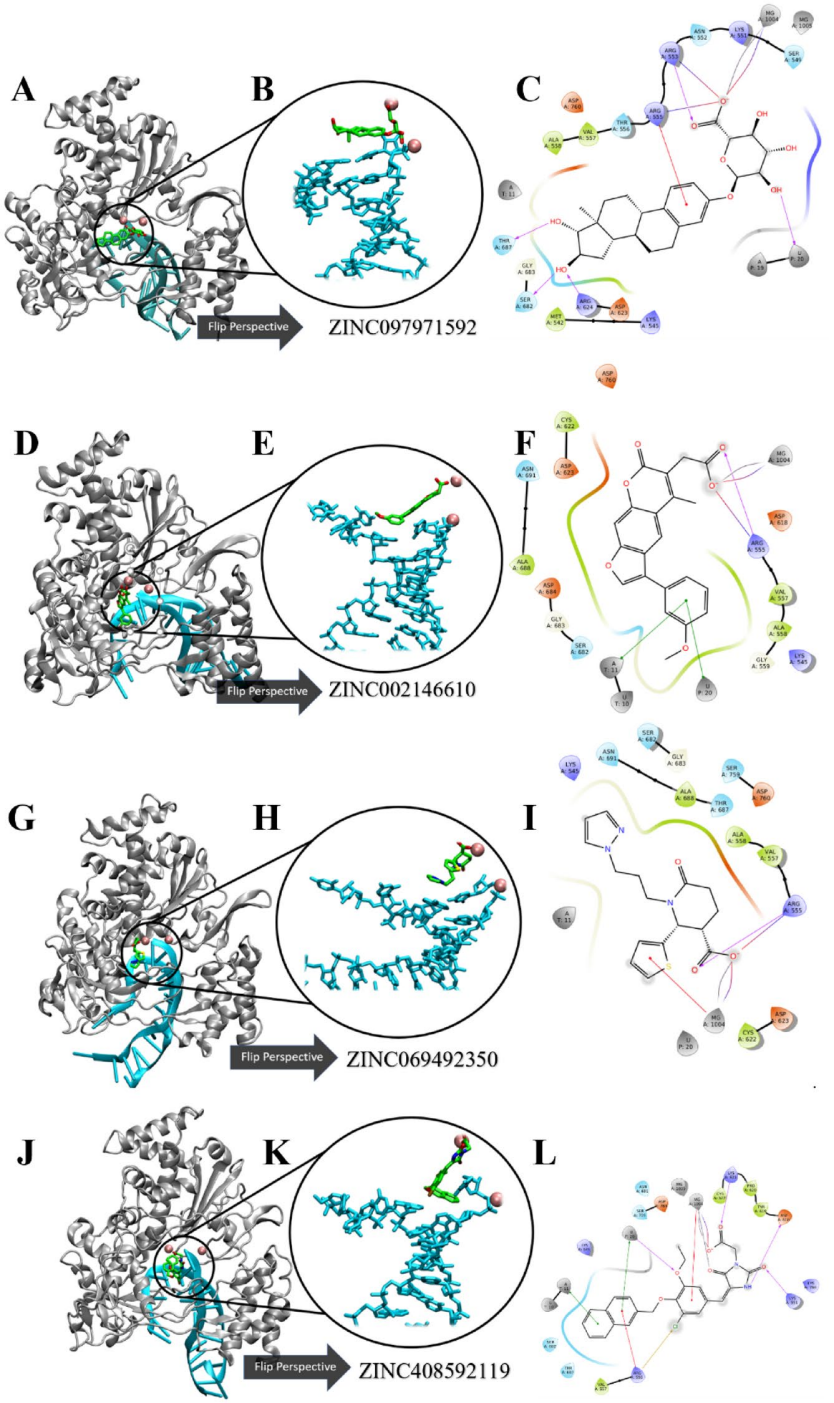
Ligand–receptor interaction diagrams and representations of the most abundant conformational pose from MD simulation. (**A,D,G,J**) The 3D cartoon representation of SARS-CoV-2 RdRp colored in gray. (**B,E,H,K**) Zoomed and rotated perspective of the template-primer RNA in complex with four ZINC candidates. (**C,F,I,L**) Detailed receptor-RNA-ligand contacts of the most abundant conformational pose of four ZINC candidates. Template and primer strands represented in blue and red licorice, respectively. Ligands are colored in green and magnesium ions are represented as pink balls.

The detailed 2D receptor-RNA-ligand contacts diagrams of the most abundant conformational pose for each of the top four ZINC candidates are shown in Fig. 8A–L. The 2d ligand–protein interactions diagram for the most abundant conformational pose of ZINC097971592, or estriol 3 glucuronide, in complex with RdRp is shown in Fig. 8C. There were several residue contacts conserved from the ATP and RTP pose. Positive residues R624, R555, and R553 formed H-bonds with mainly the polar negative carboxylate portion of the molecule, indicating that this compound is situated with the polar end in the positive catalytic region of RdRp. Additionally, ZINC097971592 also came into H-Bond contact with S682 on the hydroxyl groups on the aromatic portion. The ligand–protein contacts summary diagrams for these four ZINC candidates are displayed in Table S7 along with the total simulation protein–ligand contact heatmap to supplement in Table S11. In this, several of the residues seen in contact with the most abundant pose were also maintained with large occupancy through the simulation. Residues R555 and R624 established interactions with ZINC097971592 with 89% and 74% occupancy, respectively. Interestingly, K798 formed a salt-bridge with the negative oxygen with a relatively high occupancy value of 85%. Contacts with lesser occupancy were S682 with a 44% occupancy and R551, R553 of 50% or less. This compound’s enantiomers were identified in a previous VirtualFlow in that there was activity towards Papain-like protease (plpro), nucleoprotein, nsp10, nsp14 and orf7a in addition to nsp12^76^.

In Fig. 8F, the psoralen derivative ZINC002146610 also shows some similarity with the RTP pose and with the other ZINC hits in that the polar carboxylate group is facing inwards towards the positive amino acid region where triphosphate tail usually lays. Also, this compound formed pi–pi stacking interactions on the psoralen ring with nucleotide U20 on the primer strand and A11 of the template strand of RNA. Additionally, the polar end formed salt bridge and H-Bond interactions with R555 and the nearby Mg^+^ ion. The ligand–protein contacts summary for ZINC002146610 shows similar contacts seen in the most abundant pose. Positive amino acid R555 maintained an occupancy of 113% with the polar region of the molecule, meaning there were additional contacts seen with residue in addition to the full simulation time. Like the most abundant pose, the magnesium ion maintained full contact with the polar end via salt-bridge interactions which was seen to be further stabilized by D618. Additional stabilizing water-bridges were established with occupancies of 47% and 48% with D623 and R555, respectively. Interestingly, this compound as well as its derivatives have seen previous attention in being low toxicity potential selective and reversable inhibitors of (B5i) or the chymotrypsin-like subunit of human immunoproteasome which is associated with the treatment of autoimmune diseases and various types of cancer^77^.

In Fig. 8I, conserved residue R555 is seen in contact with the polar end of ZINC69492350 in similar fashion to the previous ZINC compounds in addition to the RTP system. Amino acid R555 has been seen to be crucial in the activity of small molecule binding to RdRp in this study as well as others^7–11,31,39–41,73,78,79^. In this binding pose of ZINC69492350, R555 forms a salt bridge to the negatively charged oxygen on the carboxyl group, with an additional hydrogen bond contact with the partially negative doubly bonded oxygen on the carboxyl end. Likewise, the aromatic portion of the molecule faces in direction with the hydrogen pairing nucleotide U10 on the template strand RNA. Surrounding amino acids are also conserved which includes D760, D623, S682 and K545. Lastly, the magnesium ions are seen to stabilize ZINC69492350 with pi-cation interactions with the thiophene portion. In the ligand–protein contacts summary diagram in the supporting information, R555 maintained full occupancy with the addition of other interactions less seen with a value of 150% with the polar carboxyl end of the molecule. Like the other selected ZINC compounds, D623 formed stabilizing water-bridge interactions with the same polar end with a value of 33%. Polar residue T687 contacted ZINC69492350 55% of the simulation, unlike the other compounds where it served as a spectator.

In Fig. 8L, ZINC408592119 lies in a similar binding pose to ATP and RTP. According to the amino acid contacts seen in the most abundant conformational pose of the simulation. Positive residues K551, K545, K621 and R555 were all seen to form hydrogen bonds and pi-cation interactions with partially positive groups on the polar ends of the molecule. Negative residues such as D618 and D760 are seen to form hydrogen bonding with partially positive portions such as on the imidizolidine group. The significance of D618 is consistent with previous studies^39–41,73,78,79^. In the ligand–protein contacts summary diagram in the supporting information, positive residue K551 maintained an occupancy of 34% in the same fashion as the most abundant pose. Magnesium also contributed full occupancy with the polar carboxyl end of the molecule consistent in Table S7 and other ZINC candidates. Lastly, in addition to forming hydrogen bonds with the imidazolidine portion, also formed a water-bridge 76% of the simulation. Table 4 is the receptor-ligand contacts with an occurrence of greater than 30% tabulated for the ATP, RTP and four ZINC hit complexes. Clearly, positive residues K621, R555, R553 and K551 all induced contact with more than two-thirds of the ligands selected, with R555 meeting all 5 ligands. Likewise, negative residues D623, D798 and D760 saw interaction with at least half of the ligands. Lastly, hydrophobic residue N691 interacted with all the selected ligands apart from ZINC408592119.

**Table 4:** Ligand–protein contacts during simulation from ATP, RTP and the four ZINC compound systems.

Residue	ATP	RTP	ZINC002146610	ZINC069492350	ZINC097971592	ZINC408592119
Y456	−	−	−	−	+	−
K545	+	−	−	+	−	−
A547	+	−	−	−	−	−
S549	+	−	−	−	+	−
A550	−	−	−	−	+	+
**K551**	** + **	** + **	** − **	** − **	** + **	** + **
**R553**	** − **	** + **	** + **	** + **	** + **	** + **
**R555**	** + **	** + **	** + **	** + **	** + **	** + **
T556	−	−	−	−	+	−
V557	−	−	+	+	−	−
A558	−	−	−	−	+	−
D618	−	−	+	−	−	+
Y619	−	−	+	−	−	+
**K621**	** + **	** + **	** + **	** − **	** − **	** + **
**D623**	** − **	** + **	** + **	** + **	** − **	** + **
R624	−	−	+	−	+	−
C662	−	−	+	−	−	−
S682	−	−	−	−	+	+
T687	−	−	−	+	+	−
A688	−	−	+	−	−	+
**N691**	** + **	** + **	** + **	** + **	** + **	** − **
**D760**	** − **	** + **	** + **	** − **	** − **	** + **
D761	+	−	−	−	−	−
**K798**	** + **	** + **	** − **	** − **	** + **	** + **
E811	+	−	−	−	−	−
R836	+	−	−	−	−	−

Ligand–protein contacts may consist of hydrogen bonding, lipophilic, hydrophobic and electrostatic interactions. Contacts shown are present in at least 30% of trajectory time.

Bold are residues that interacted with at least 3 of the six tested ligands.

**Figure 9:**
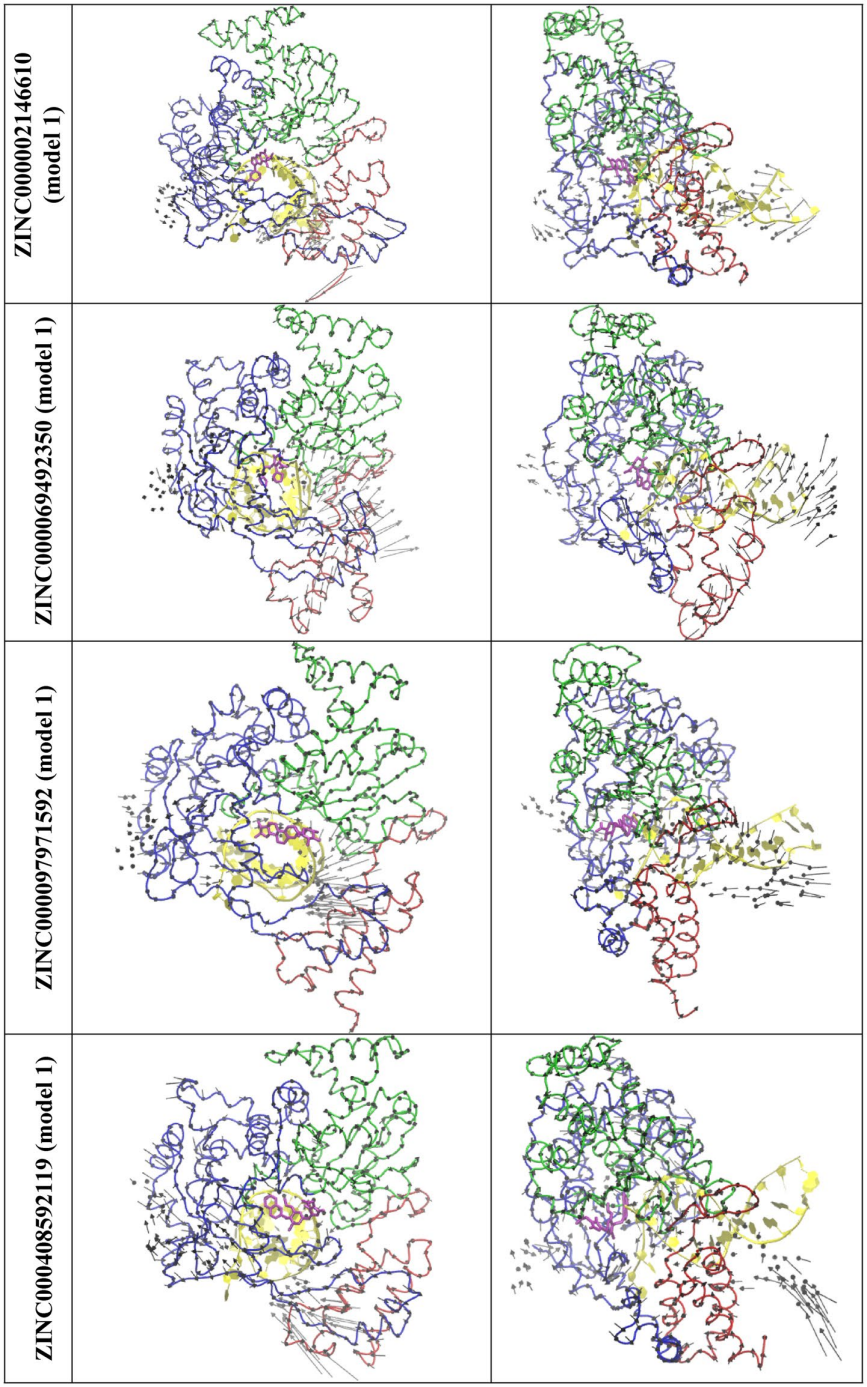
The top view (left) and the side view (right) of a selected normal mode from the top 4 ZINC systems that is most similar to the mode 1 of RTP system. RNA (yellow) and, Thumb domain (Red), Palm domain (Green), Finger domain (Blue) of RdRp is shown ribbon.

The top 5 normal modes were obtained for the four top zinc compound systems (ZINC000002146610/Fig. S6c, ZINC000069492350/Fig. S6d, ZINC000097971592/Fig. S6e, and ZINC000408592119/Fig. S6f). The visual inspection suggest that although these modes of the four compounds also undergo an opening to closing motion, the magnitude is small which is more similar to RTP than ATP. Figure 9 show the mode 1 from each system. Figure S7 show the mode that is most similar to the mode 5 of the ATP system. It can be noted that the ZINC000408592119 undergoes similar opening to closing motions in comparison to the ATP system. It was also observed that ZINC000002146610, ZINC000069492350, and ZINC000097971592 have similar opening and closing motions in comparison to the RTP system.

The absorption, distribution, metabolism and excretion (ADME) properties were also explored for these four compounds in addition to ATP and prodrug RTP using the SwissADME webserver^75^ (Table 5). Within the four non-nucleoside analogs, three of them were reported to have high predicted GI absorption and cytochrome P450 inhibition for CYP1A2, CYP2C19, CYP2C9 and CYP3A4. With these compounds being very early drug hits selected using in silico methods, obviously there is the need for experimental evaluation needed to evaluate their biological attributes. Additionally, it is still unclear how these compounds would behave and be metabolized when introduced to a biological system. Based on the predicted bioavailability, three of the four compounds possess high (56%) availability in rats.

**Table 5:** Summary of drug property predictions of ATP, RTP and top four ZINC candidates obtained from the SwissADME webserver.

Molecule	SILICOS-IT class	GI adsorption	CYP1A2 inhibitor	CYP2C19 inhibitor	CYP2C9 inhibitor	CYP2D6 inhibitor	CYP3A4 inhibitor	Bioavalability score	PAINS alerts
ATP	Soluble	Low	No	No	No	No	No	0.11	0
RDV	Slightly soluble	Low	No	No	No	No	Yes	0.17	0
ZINC097971592	Poorly soluble	High	Yes	No	Yes	No	No	0.56	0
ZINC002146610	Soluble	High	No	No	No	No	No	0.56	0
ZINC069492350	Soluble	Low	No	No	No	No	No	0.11	0
ZINC408592119	Poorly soluble	High	No	Yes	Yes	No	Yes	0.56	0

## Conclusion

In this study, we aimed to determine the binding of the active-form of remdesivir to SARS-CoV-2 RdRp in complex with template-primer RNA and magnesium ions. Using the model constructed from PDB 7BV2^44^, we then constructed a complex with ATP for further validation. We confirmed that these models were stable during the 1.0 µs MD simulation by observing the receptor/ligand RMSD and RMSF values. We then performed HTVS of 1179 compounds from the ZINC database against the most abundant conformation obtained from the RTP system. Based on their best docking score and low #stars, we selected the best 14 compounds form this list. We then subjected these 14 to an additional 200 ns relaxation MD simulation as well as MM-GBSA binding energy calculations to further elucidate more about their binding behavior and comparability to the classical nucleotides ATP and RTP. From these results, we ultimately selected the four ZINC candidates: ZINC097971592, ZINC002146610, ZINC69492350 and ZINC408592119 based on their lower MM-GBSA binding energy values than that of ATP and RTP. We also observed the detailed ligand-receptor contacts for each ZINC compounds pre and post simulation to compare their similarity in conformational pose to ATP and RTP. It was observed that R551, R553 and R555 have significant importance relating to negative motif hydrogen bond stabilization of both nucleotide phosphate tails and the ZINC polar ends. Additionally, negative amino acids D618 and D760 were seen to establish metal coordination bridges with Mg^+^ ions to further stabilization, as well as other water bridges and hydrogen bonding interactions with nearly full occupancies. These discoveries suggest that the four selected ligands from HTVS of the ZINC database could display similar or better binding affinity and inhibition activities of towards RdRp in-vitro compared to traditional nucleoside analogues.

## Supplementary Information

Supplementary Information.

## Data Availability

All data generated or analysed during this study are included in this published article and its Supplementary Information document.
