# Supplementary material for: Discovering new potential inhibitors to SARS-CoV-2 RNA dependent RNA polymerase (RdRp) using high throughput virtual screening and molecular dynamics simulations
Source: Sci Rep. 2022 Nov 21;12:19986. doi: 10.1038/s41598-022-24695-4 (PMC9676757; doi:10.1038/s41598-022-24695-4)
Supplement: Supplementary file 1 — Supplementary Information. [file 41598_2022_24695_MOESM1_ESM.pdf]

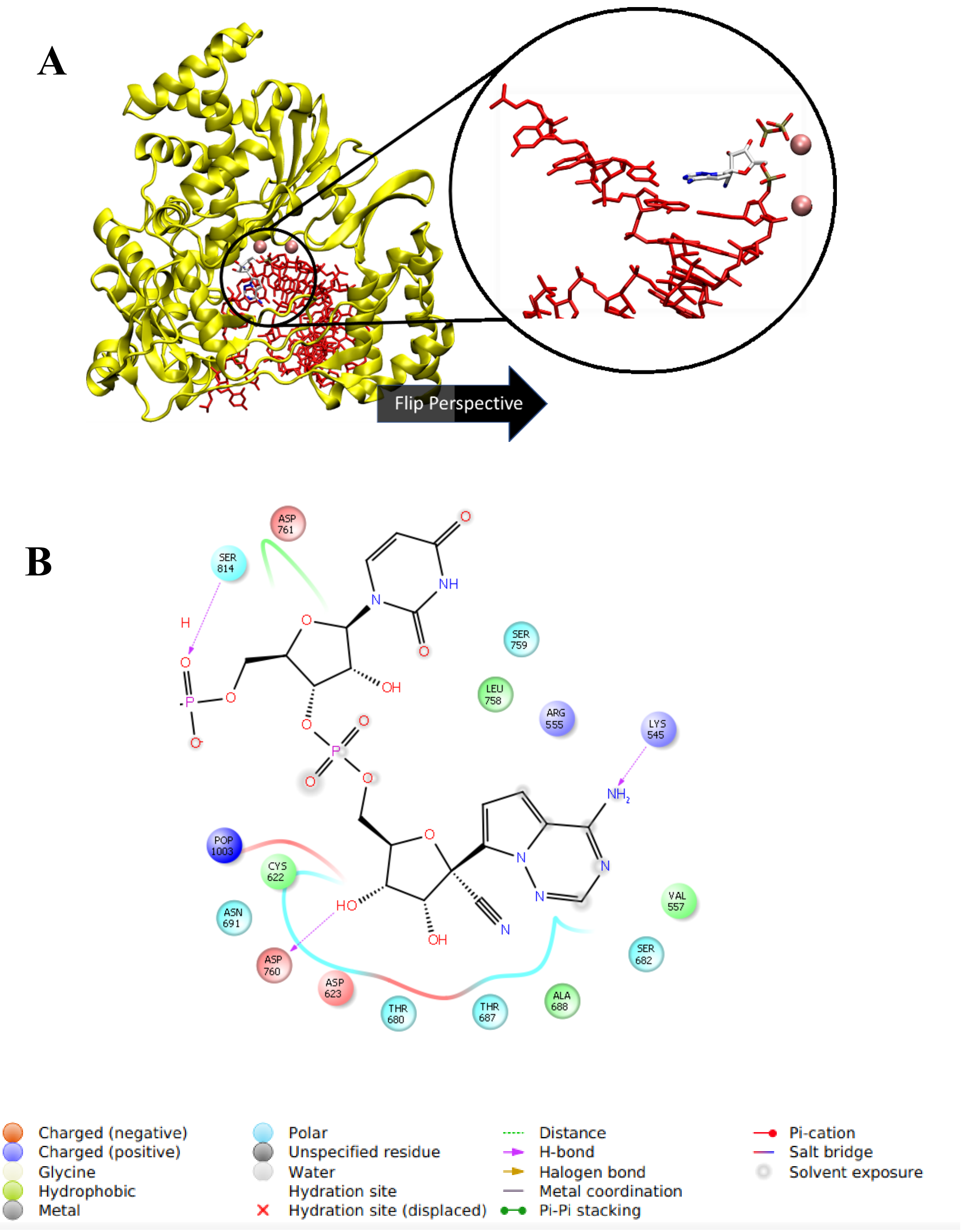

Figure S1. Crystal structure of RdRp in complex with dsRNA with covalently bound RDV. **A** is Crystal PDB: 7BV2 zoomed view of RDV (white) bound dsRNA template-primer complex with Mg ions (pink) and free diphosphate. **B** is a diagram of the interactions between covalently bound RDV and protein residues.

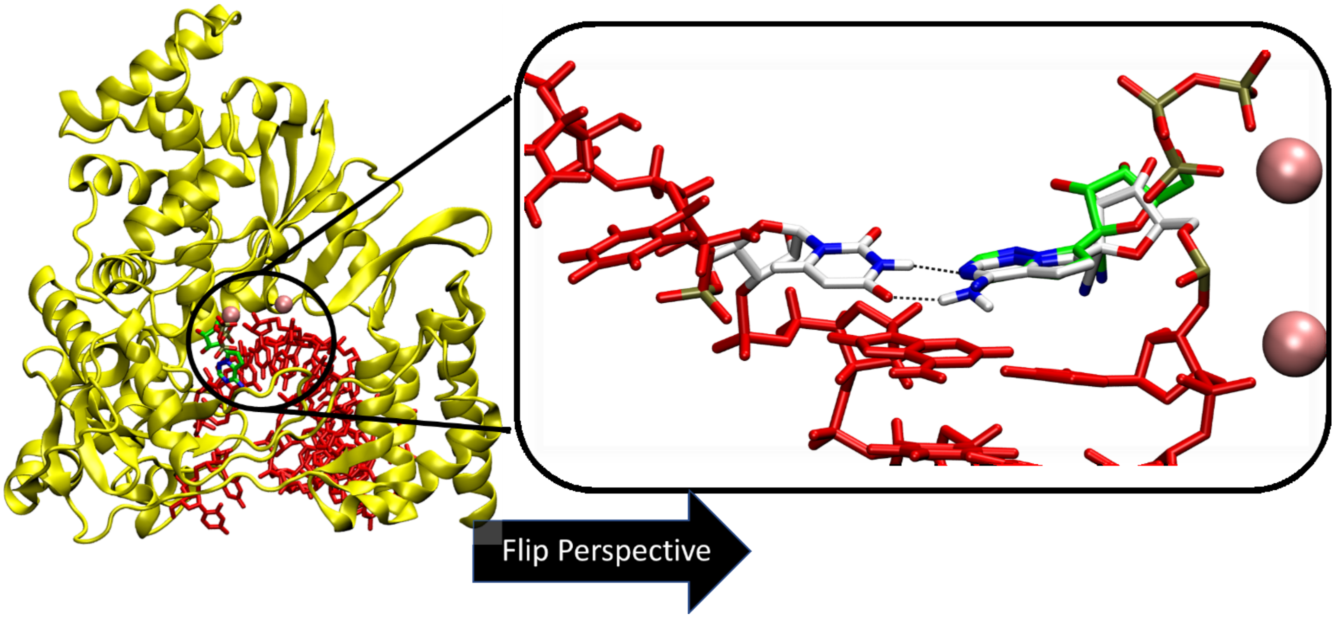

Figure S2. Superposition between our docked RTP pose and the Crystal RDV pose. Crystal RdRp is colored in yellow with dsRNA presented in red licorice. Crystal RDV is colored in white with our free form docked RTP pose colored in green. Magnesium ions presented as pink balls.

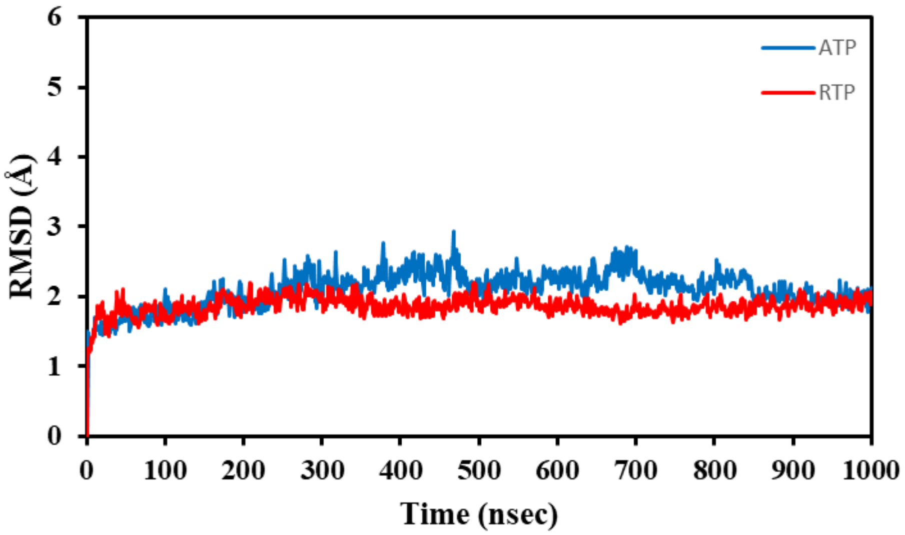

Figure S3. The RMSD values of template-primer RNA for ATP and RTP systems.

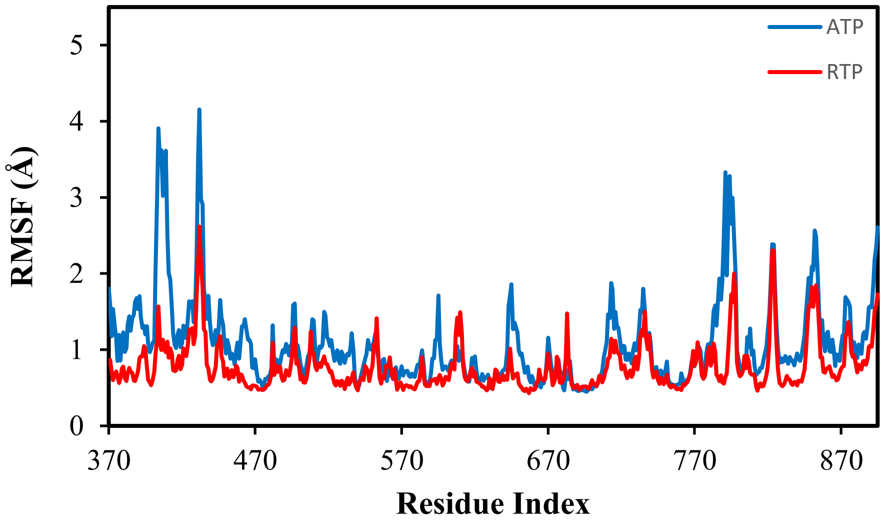

Figure S4. Protein Cα RMSF values for ATP and RTP systems over full 1 μs MD simulation time.

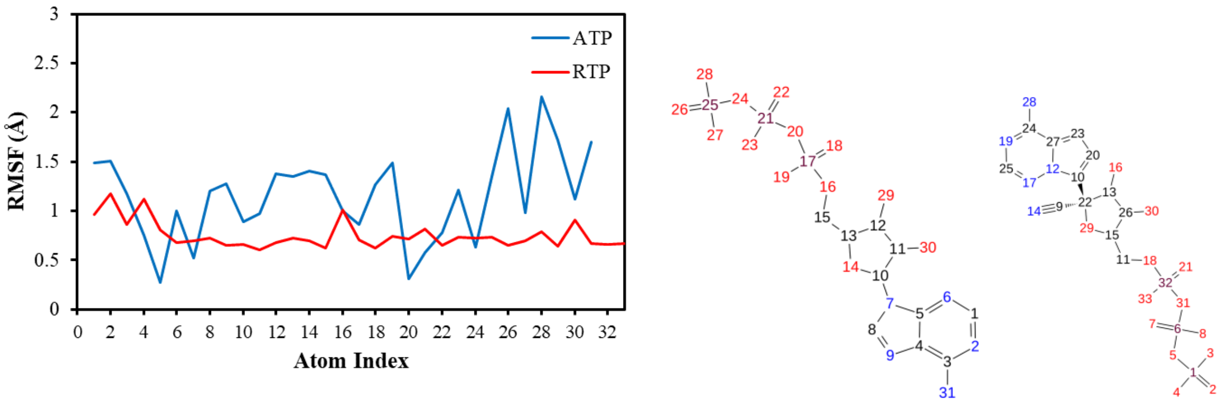

Figure S5. The RMSF values of the ligand main atoms of ATP and RTP red systems over 1000 ns simulation.

Mode	Top	Side
1	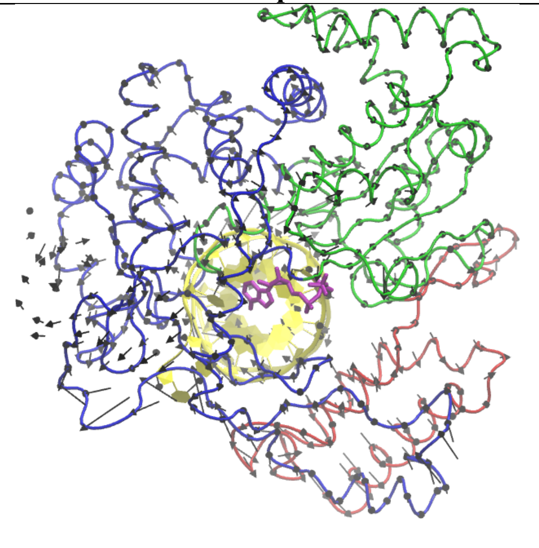	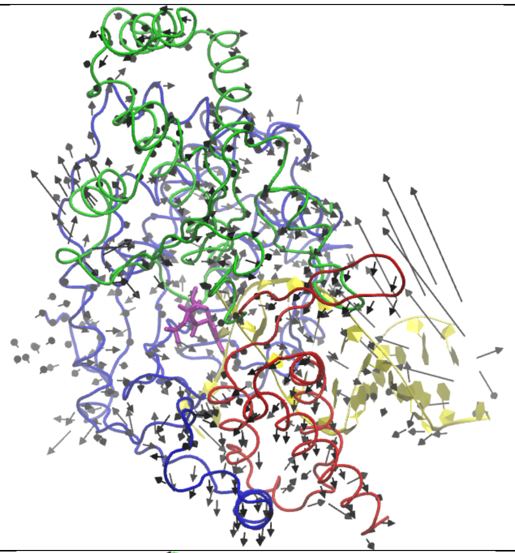
2	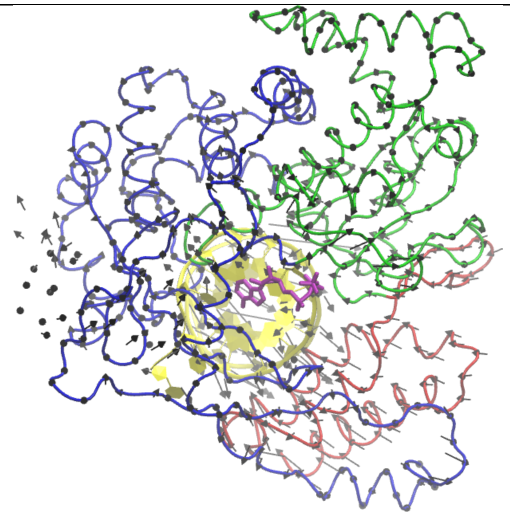	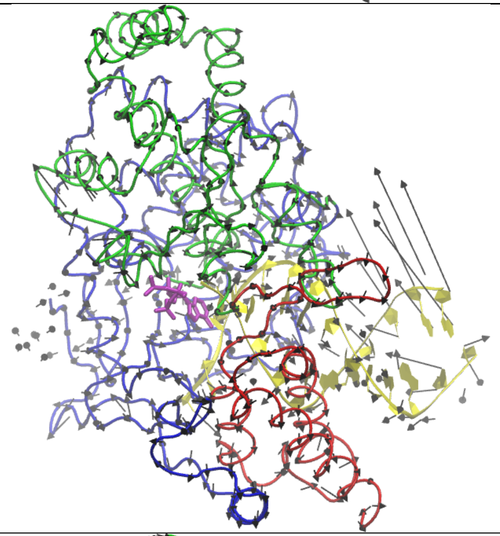
3	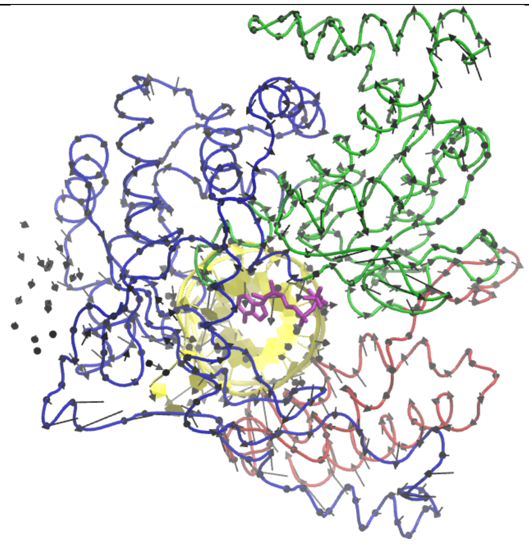	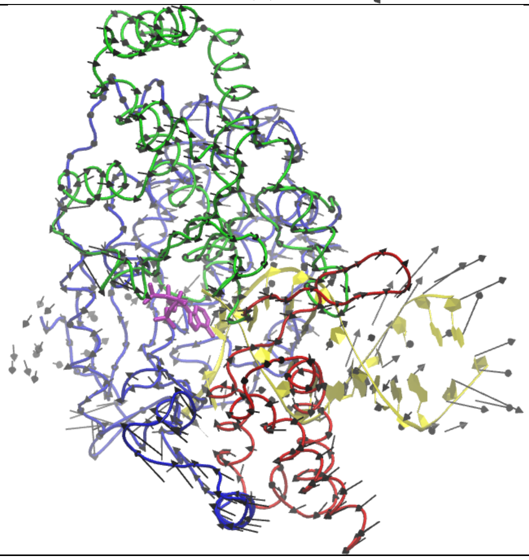

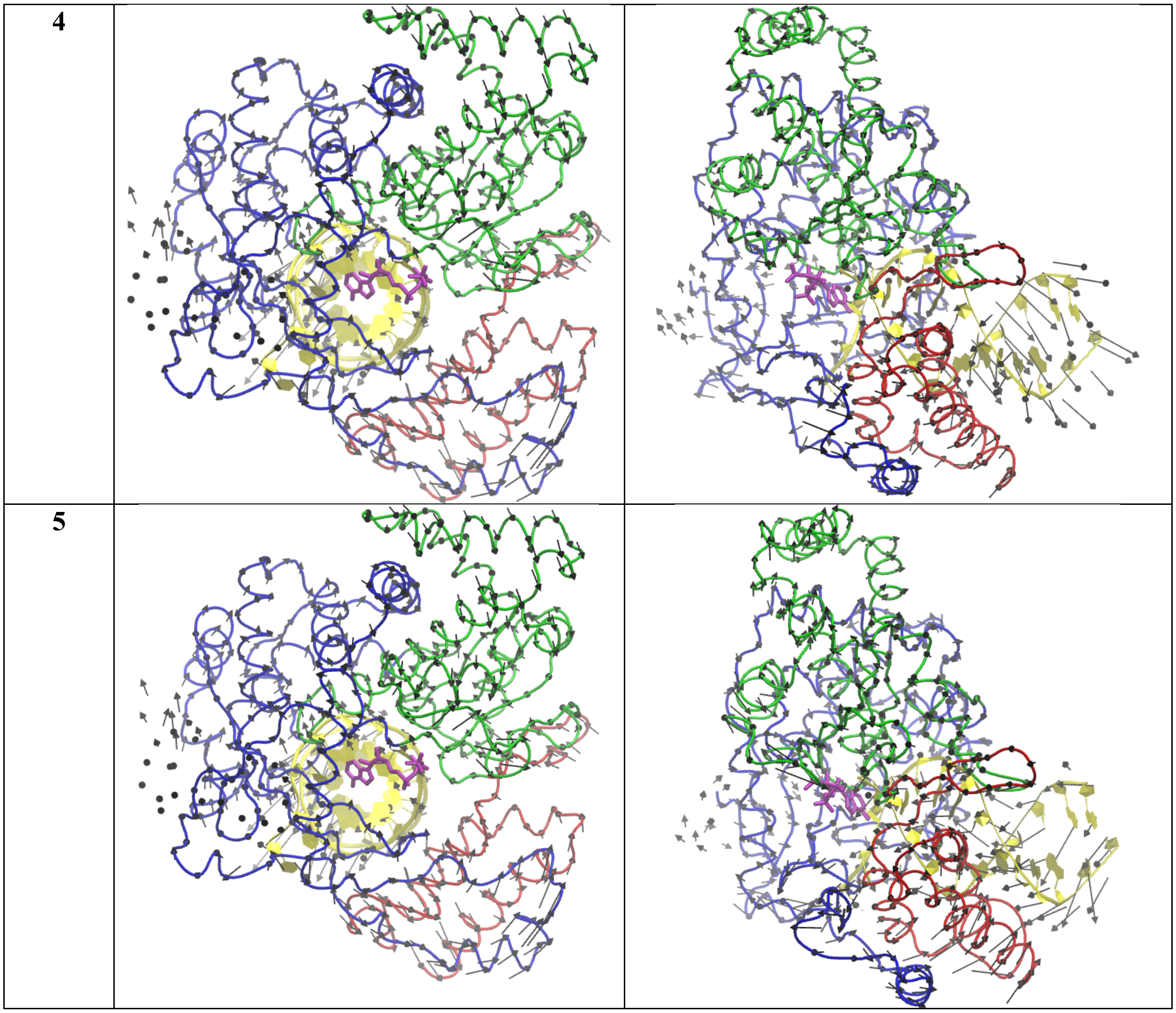

Figure S6a The top five normal modes from the ATP system. RNA (yellow) and Finger domain (Blue), Palm domain, (Green) Thumb domain (Red) of RdRp is shown ribbon.

Modes	Top	Side
**1**	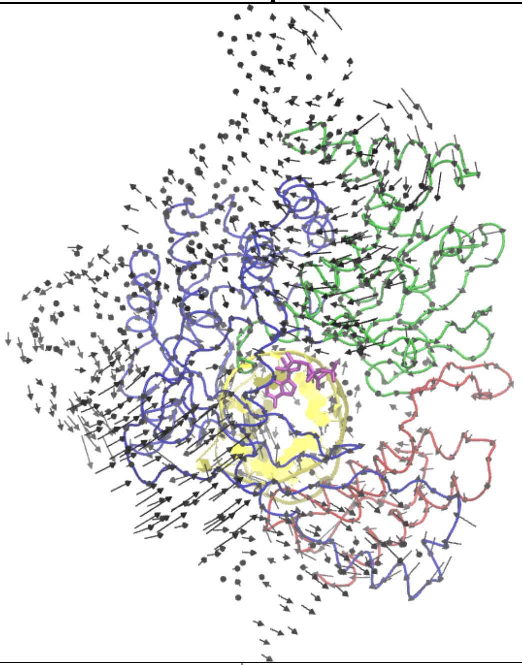	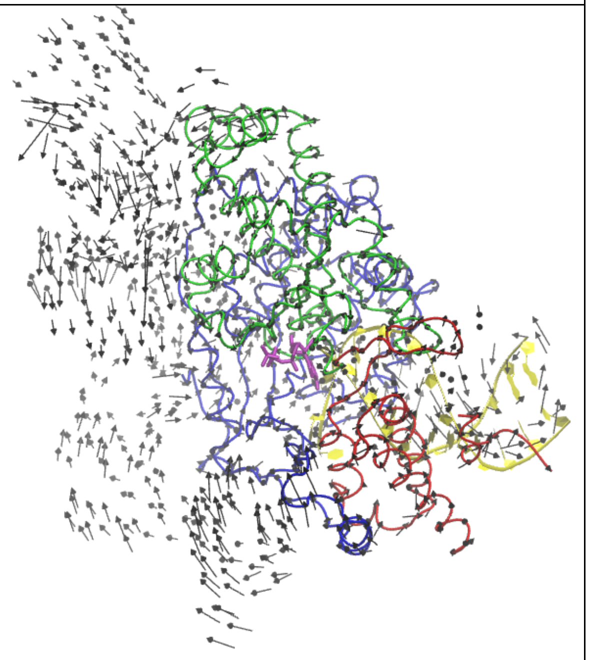
**2**	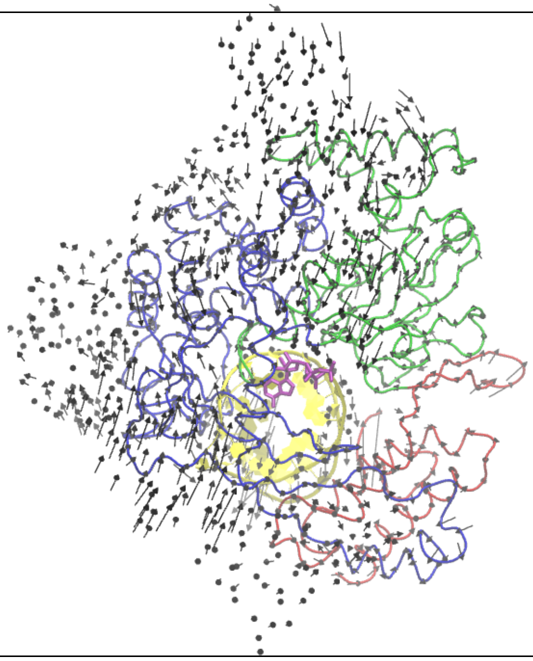	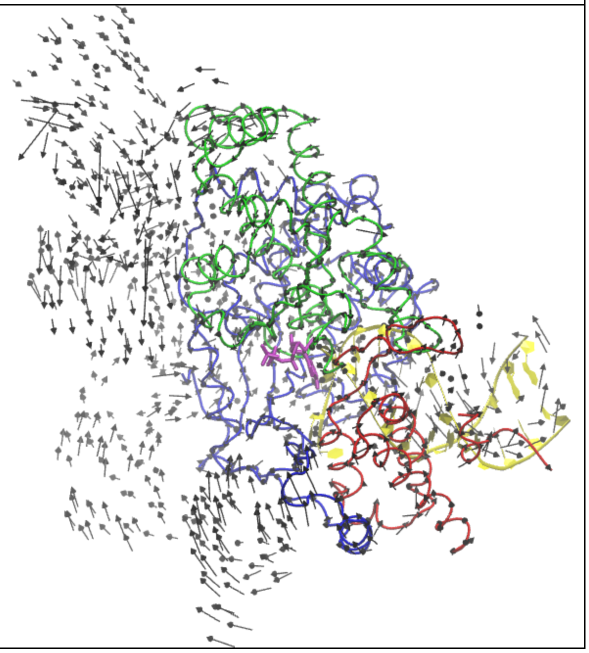

**3**	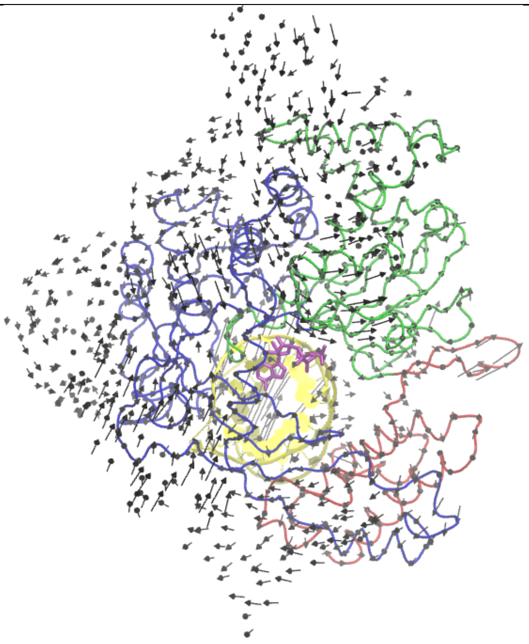	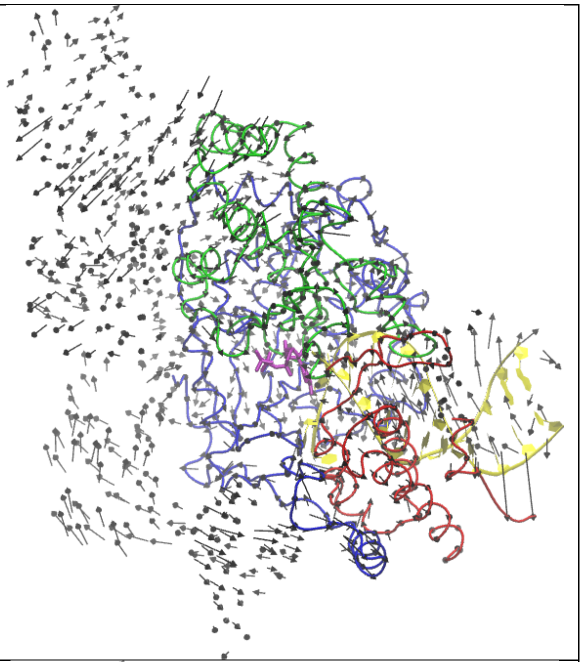
**4**	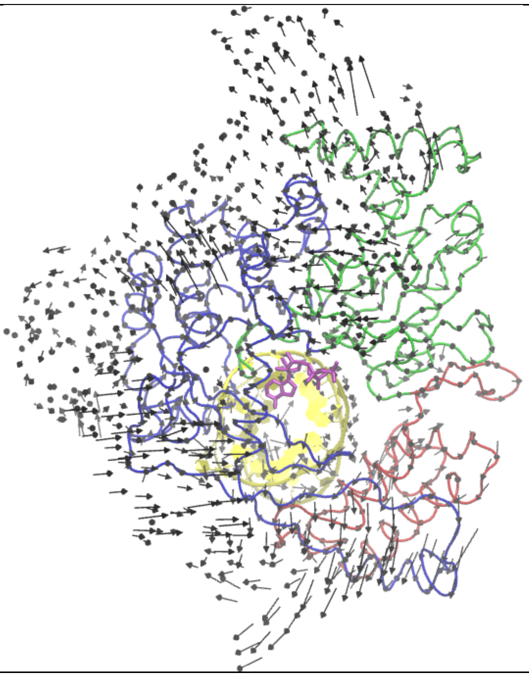	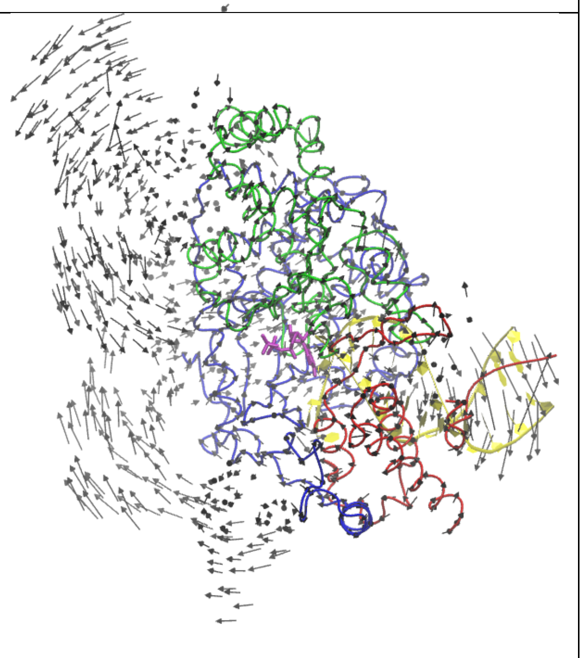

**5**	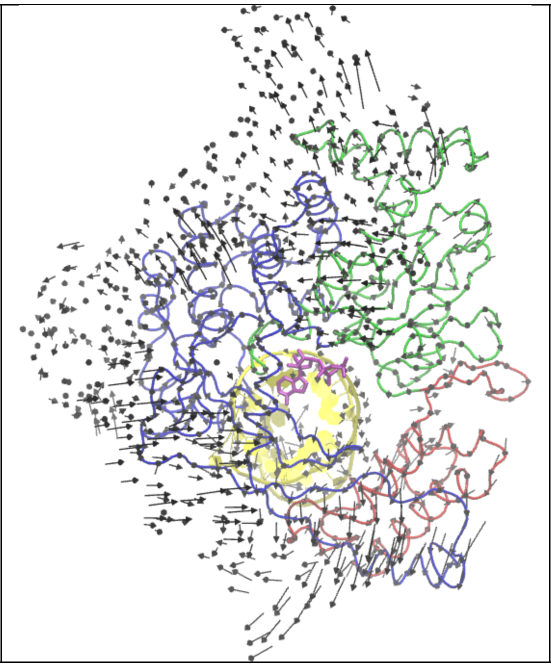	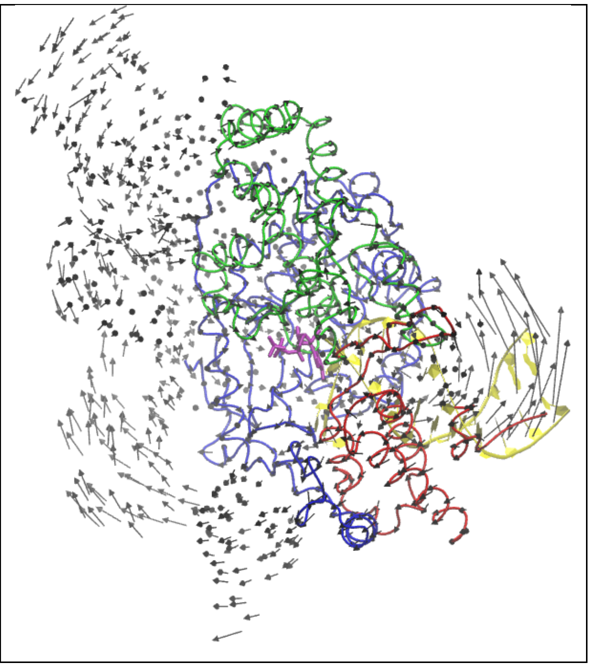

Figure S6b The top five normal modes from the RTP system. RNA (yellow) and Finger domain (Blue), Palm domain (Green) Thumb domain (Red) of RdRp is shown ribbon.

Modes	Top	Side
1	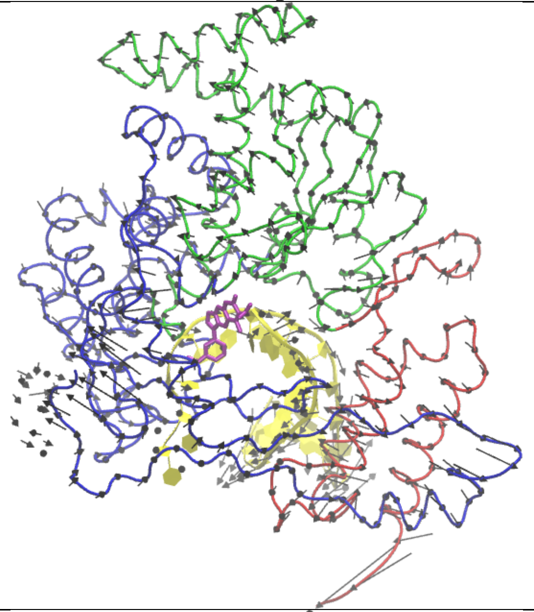	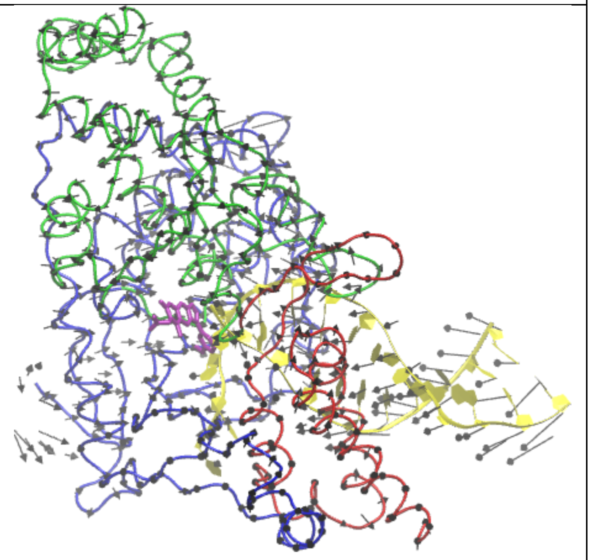
2	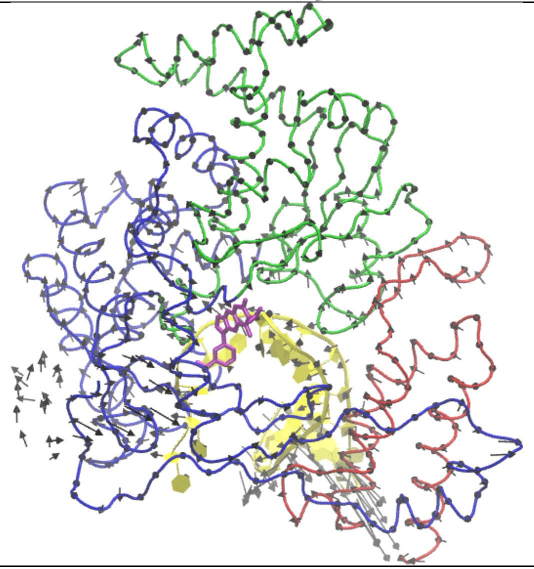	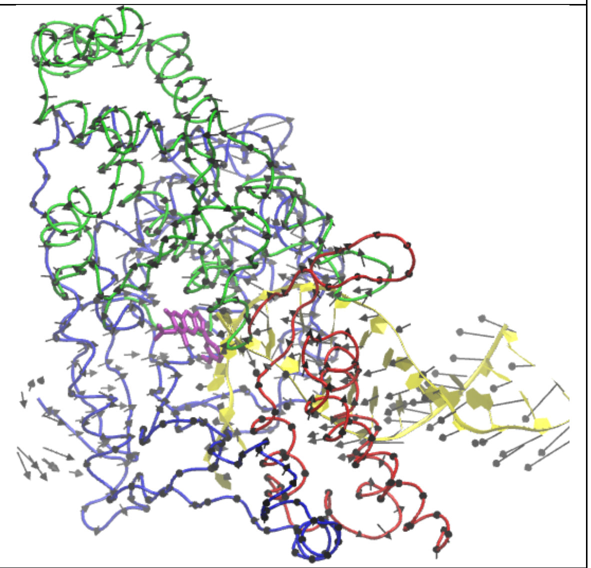

**3**	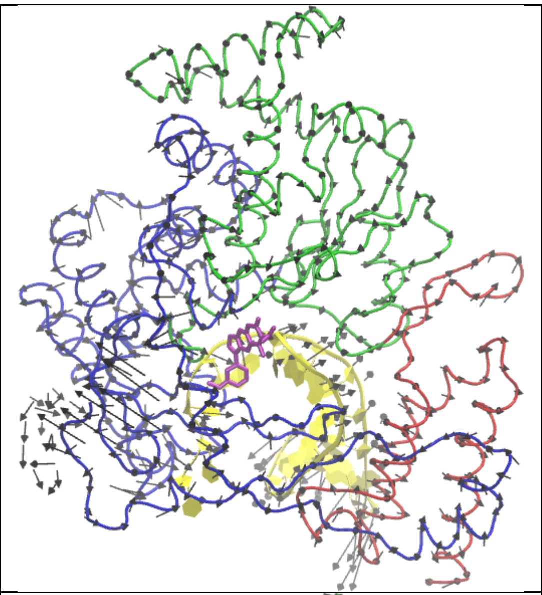	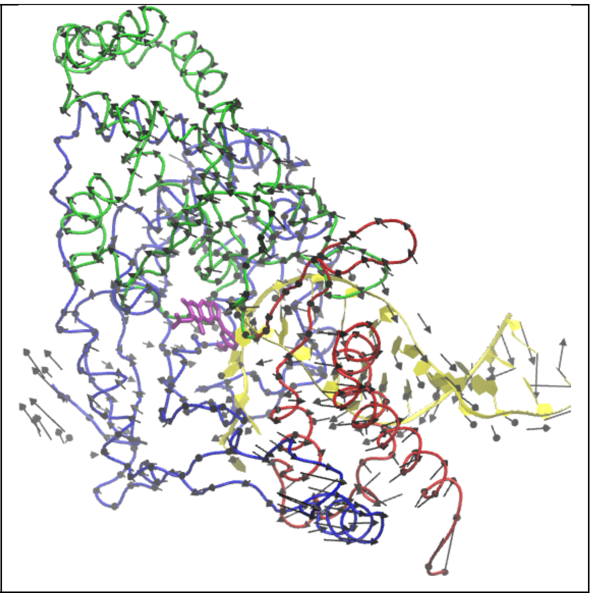
**4**	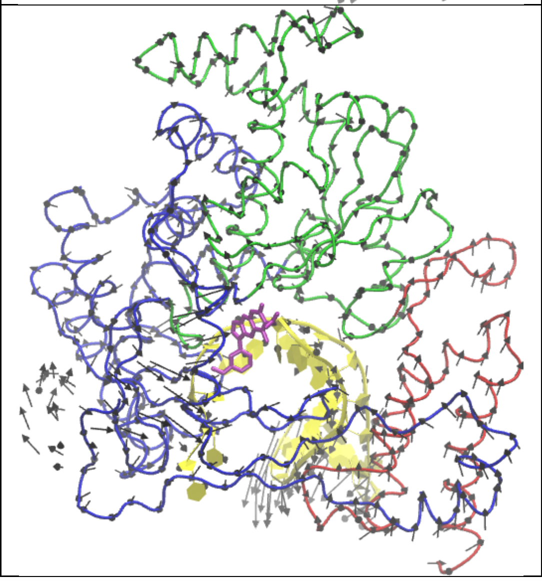	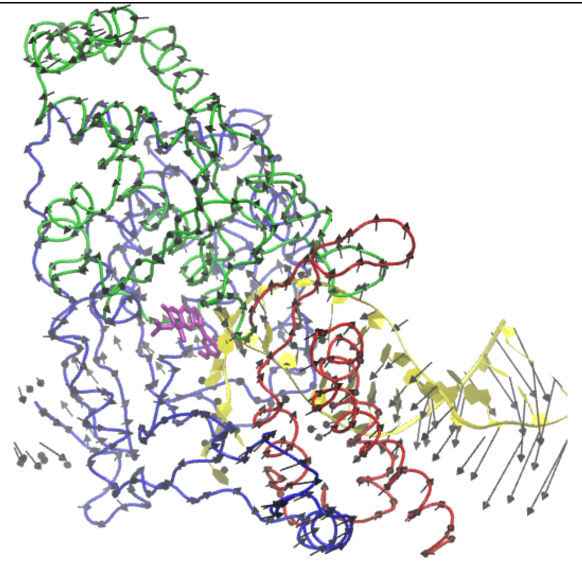

**5**	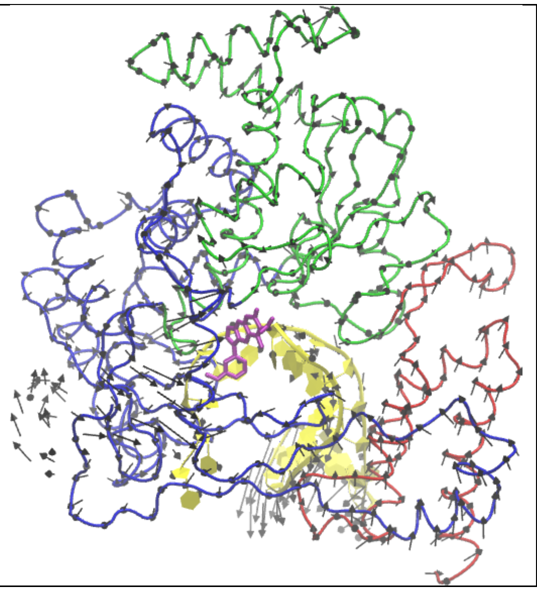	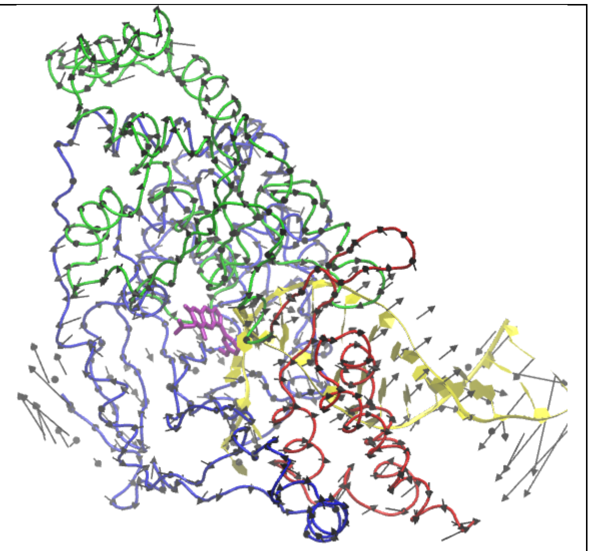

Figure S6c The top five normal modes from the ZINC000002146610 system. RNA (yellow) and Finger domain (Blue), Palm domain (Green) Thumb domain (Red) of RdRp is shown ribbon.

Modes	Top	Side
1	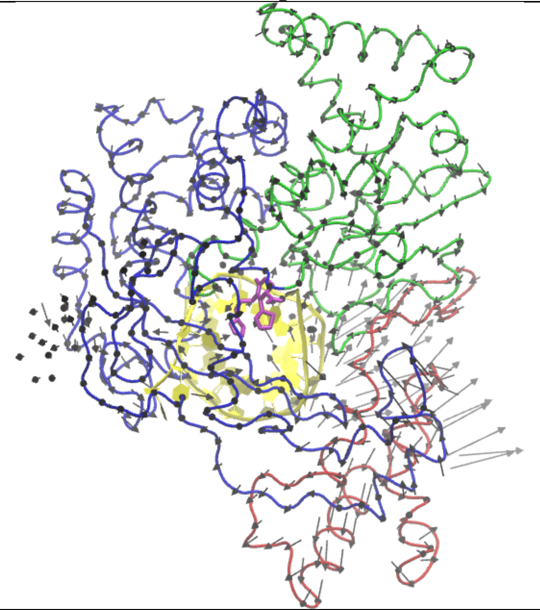	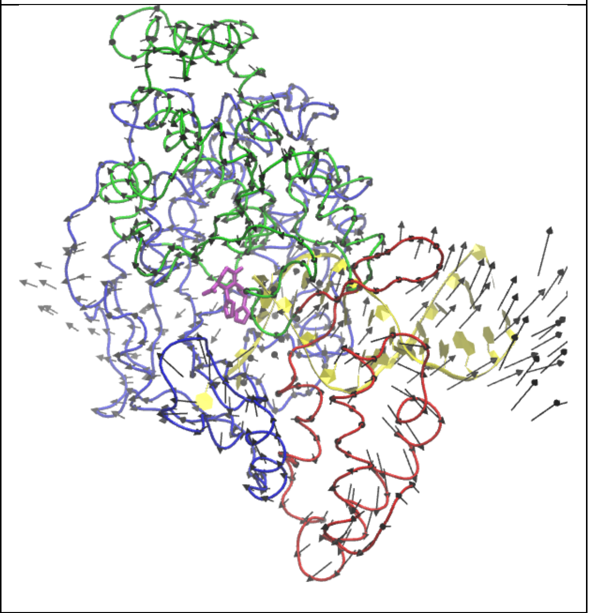
2	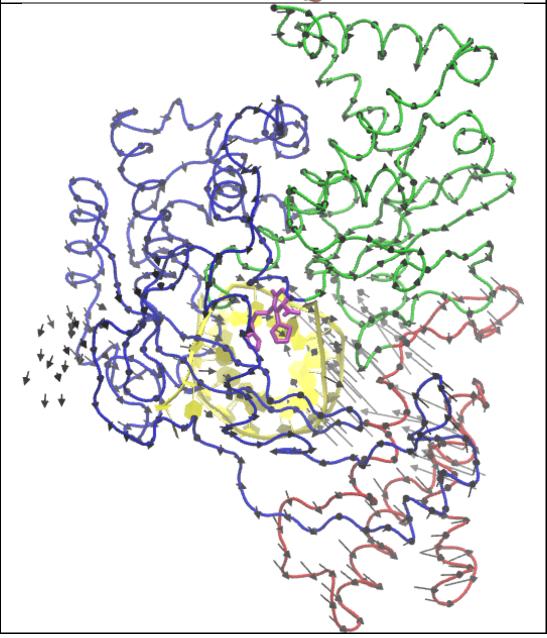	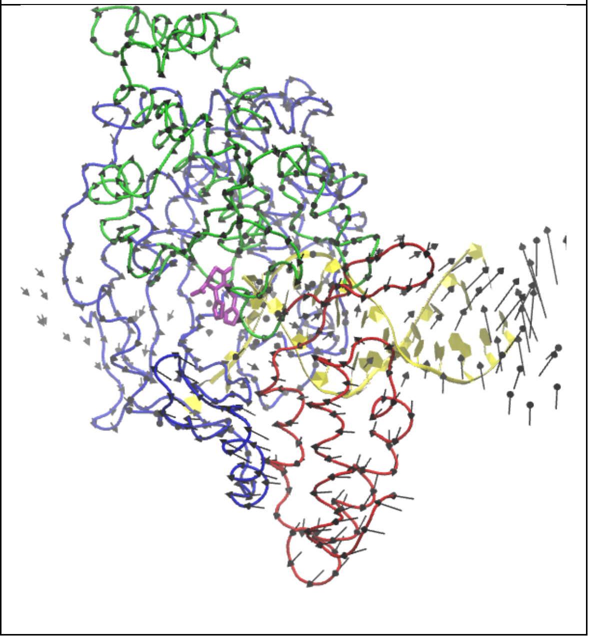

**3**	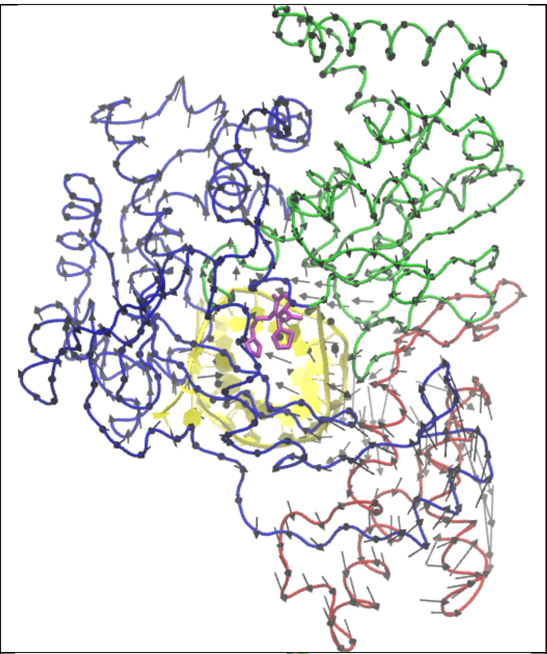	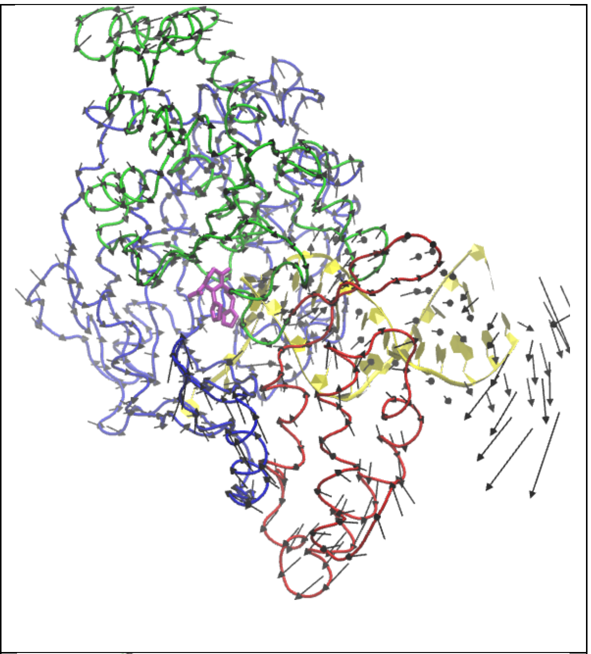
**4**	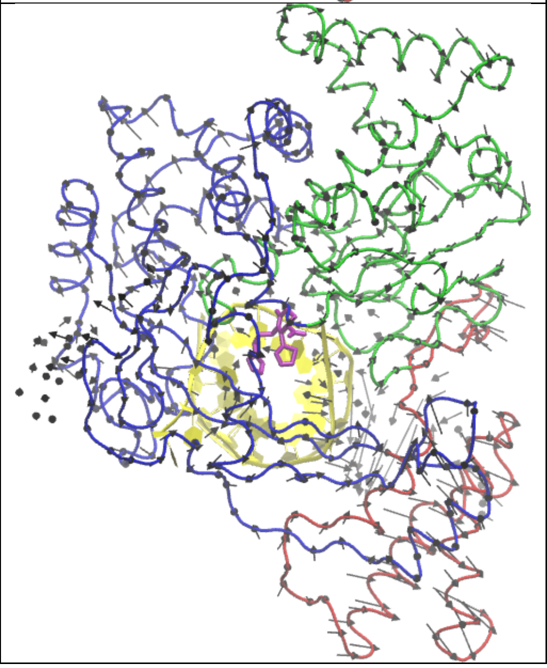	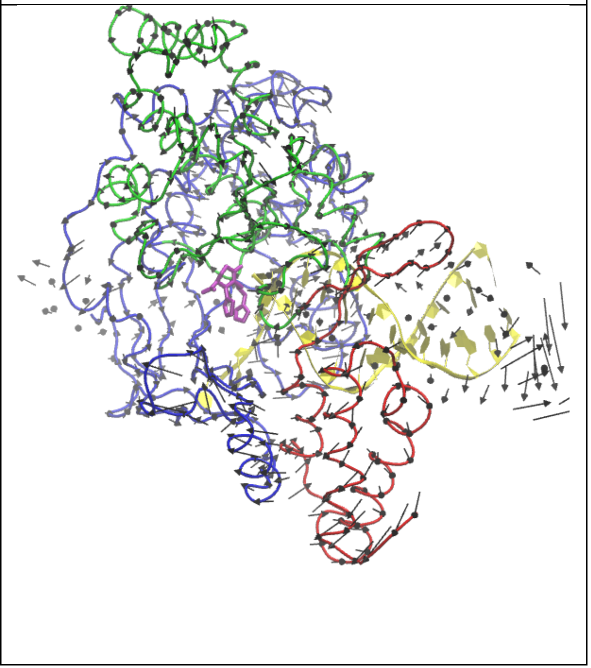

**5**	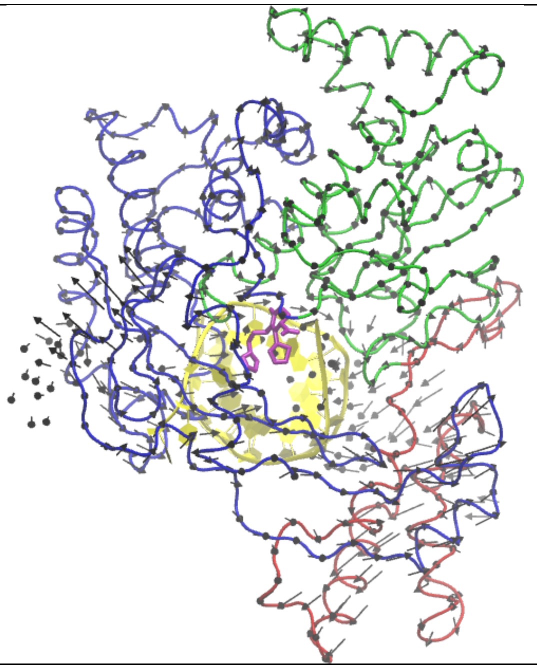	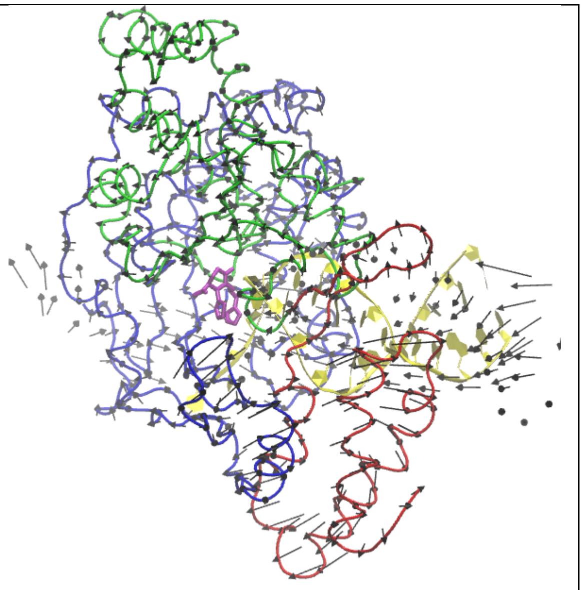

Figure S6d The top five normal modes from the ZINC000069492350 system. RNA (yellow) and Finger domain (Blue), Palm domain (Green) Thumb domain (Red) of RdRp is shown ribbon.

Modes	Top	Side
**1**	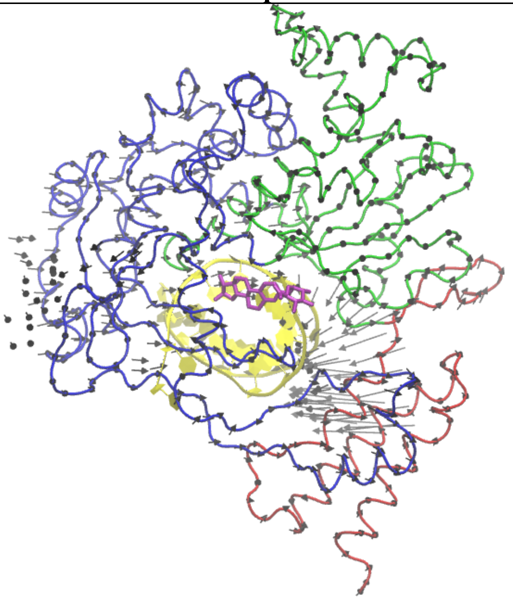	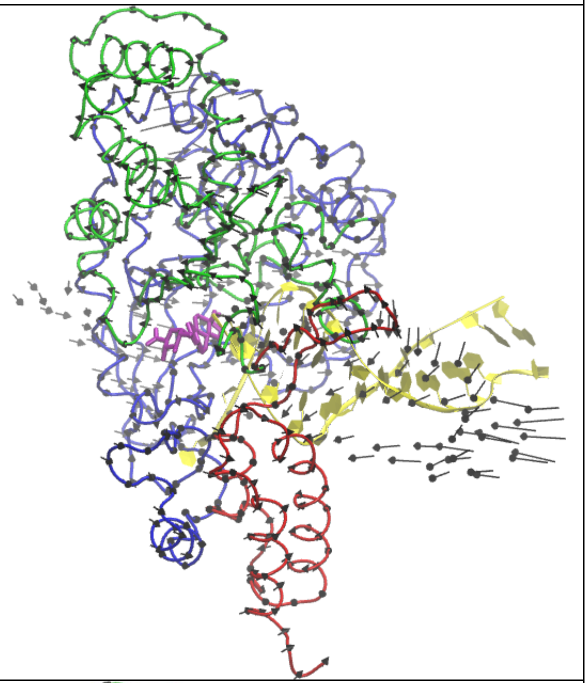
**2**	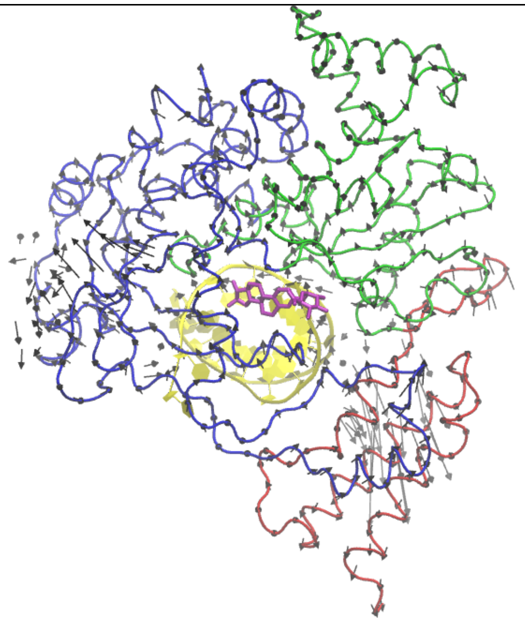	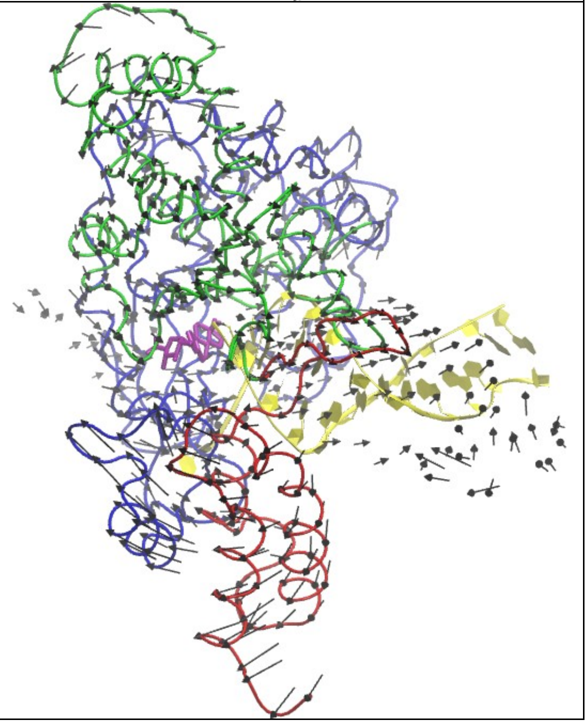

**3**	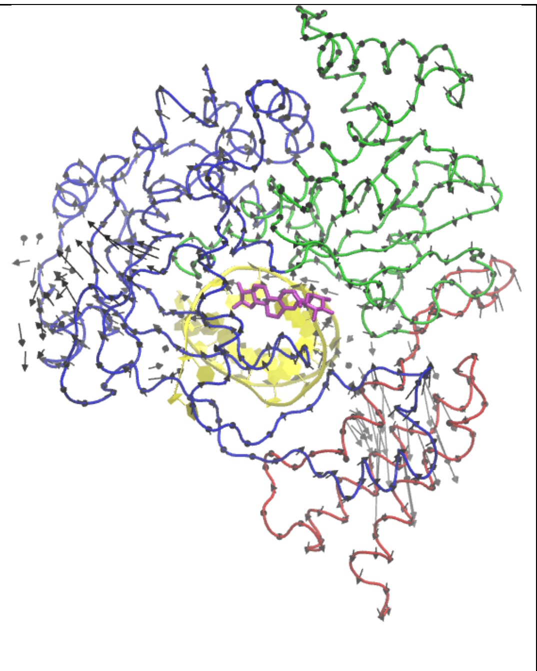	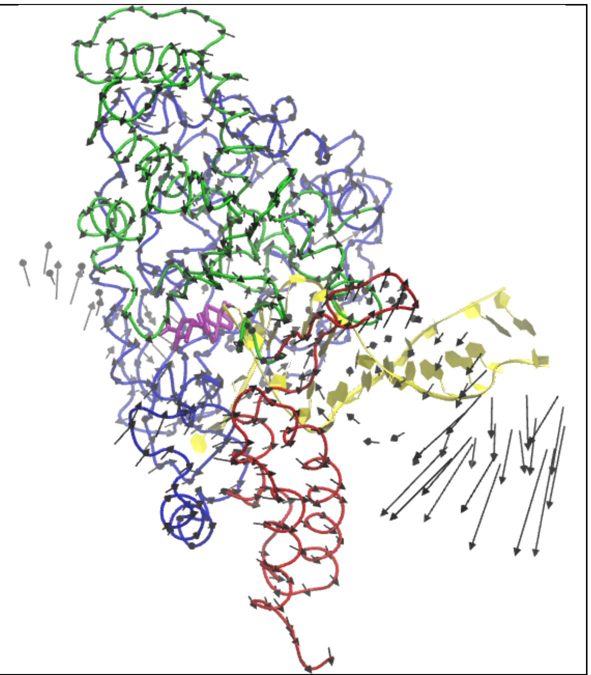
**4**	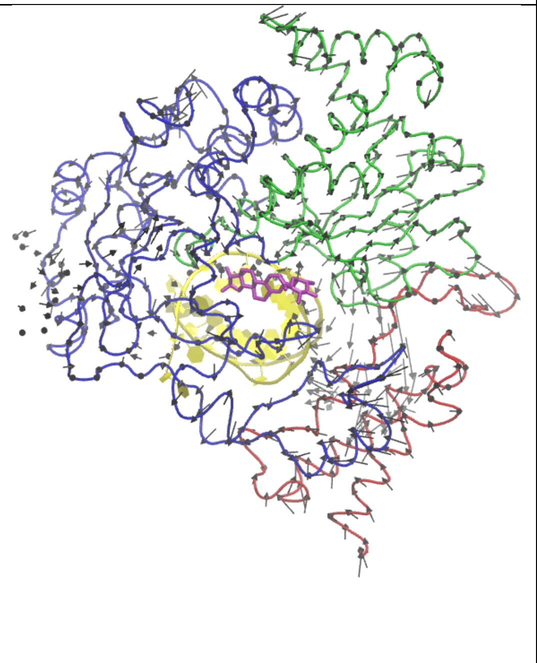	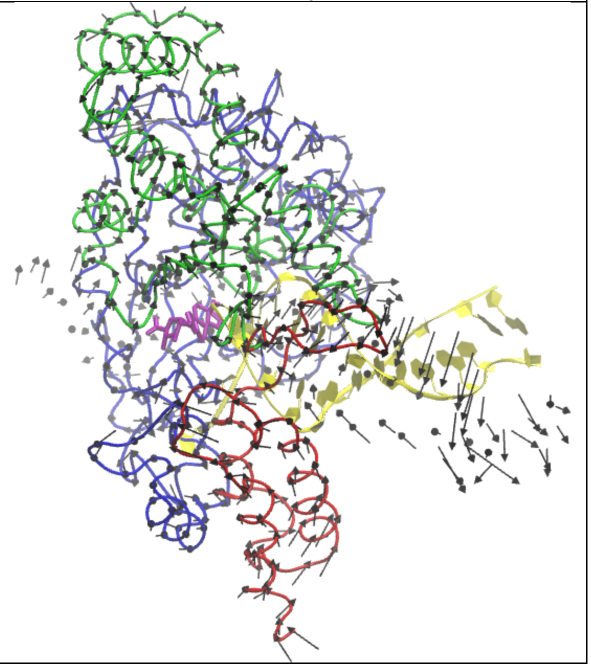

**5**	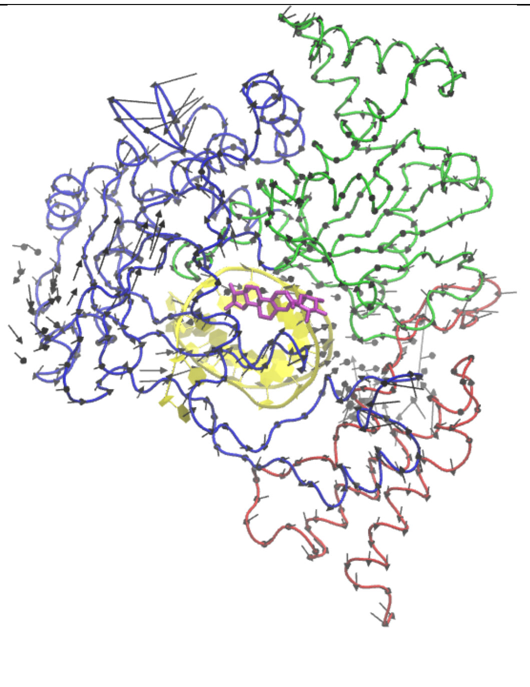	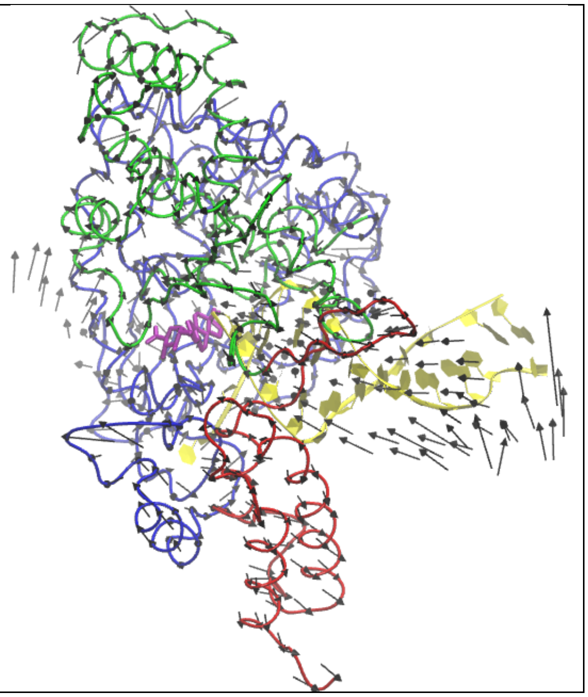

Figure S6e The top five normal modes from the ZINC000097971592 system. RNA (yellow) and Finger domain (Blue), Palm domain (Green) Thumb domain (Red) of RdRp is shown ribbon.

Modes	Top	Side
**1**	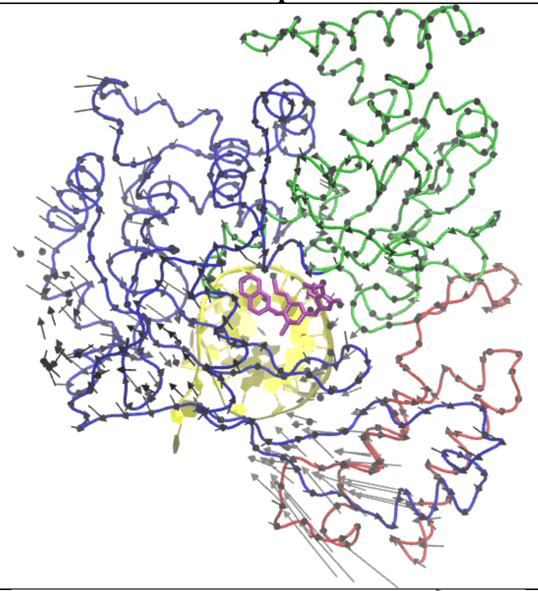	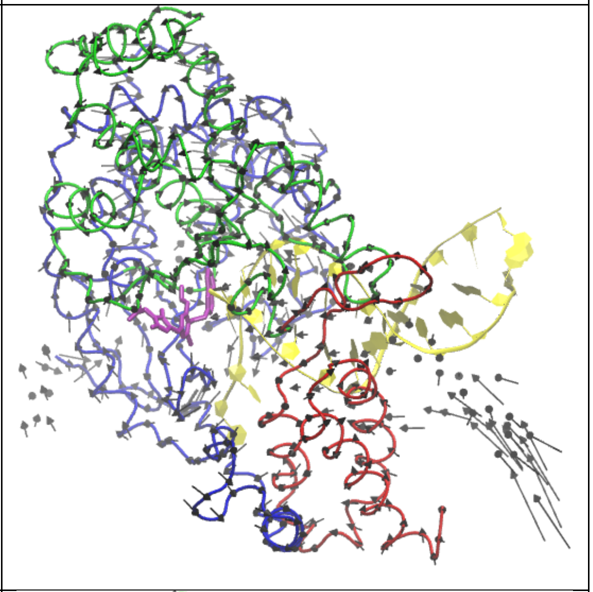
**2**	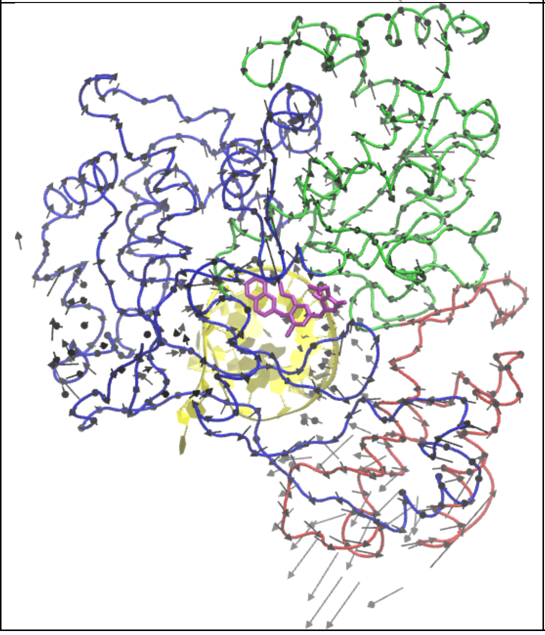	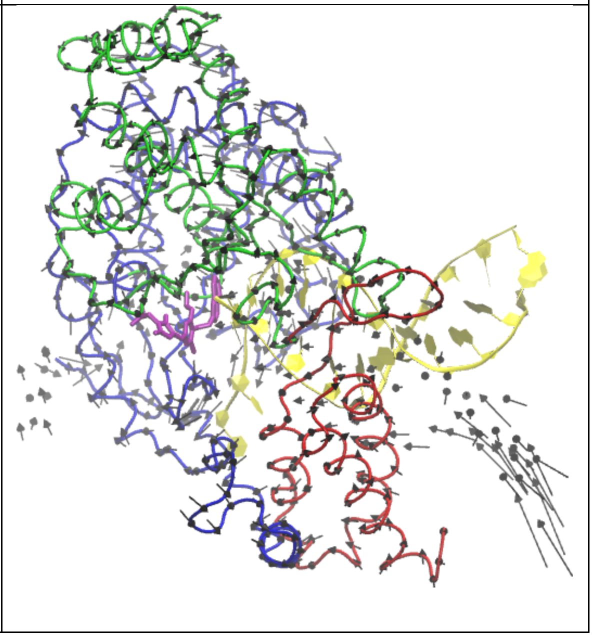

**3**	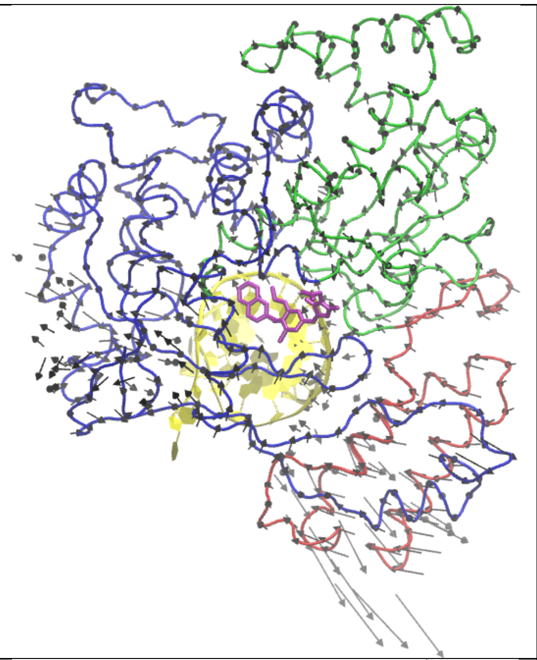	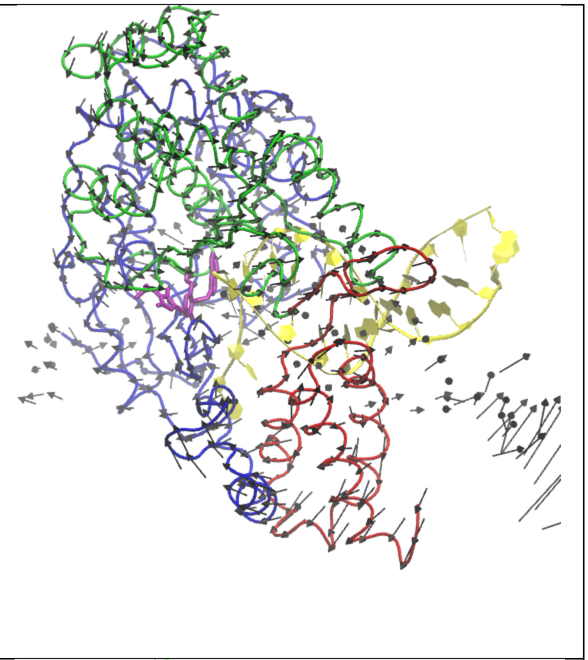
**4**	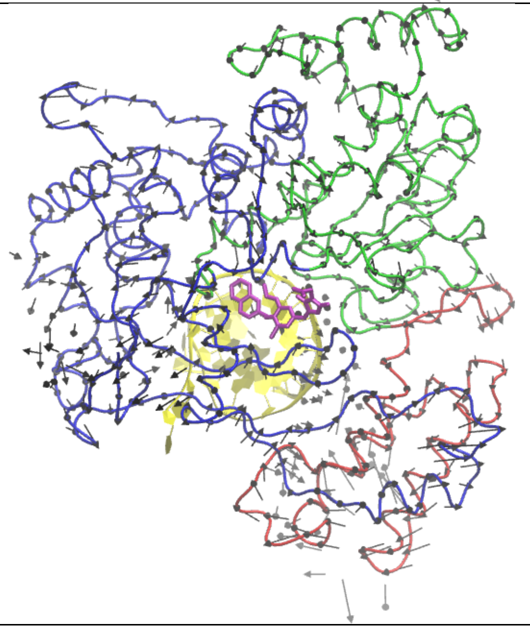	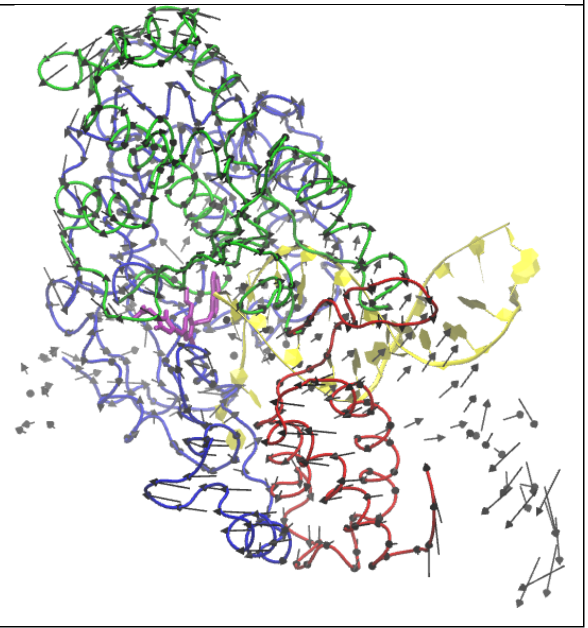

**5** /	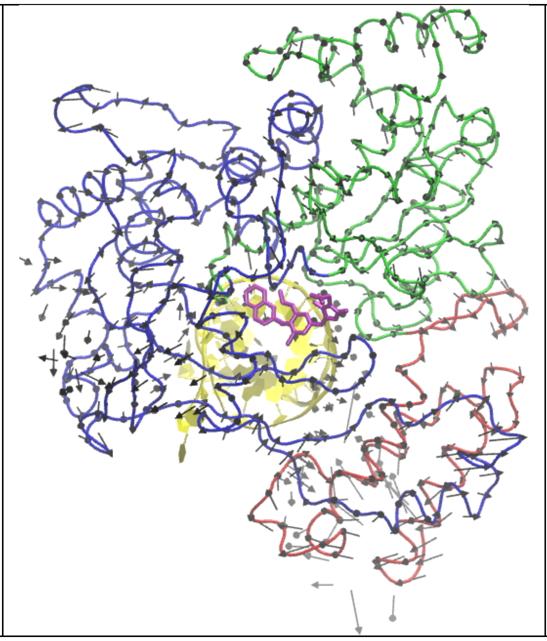	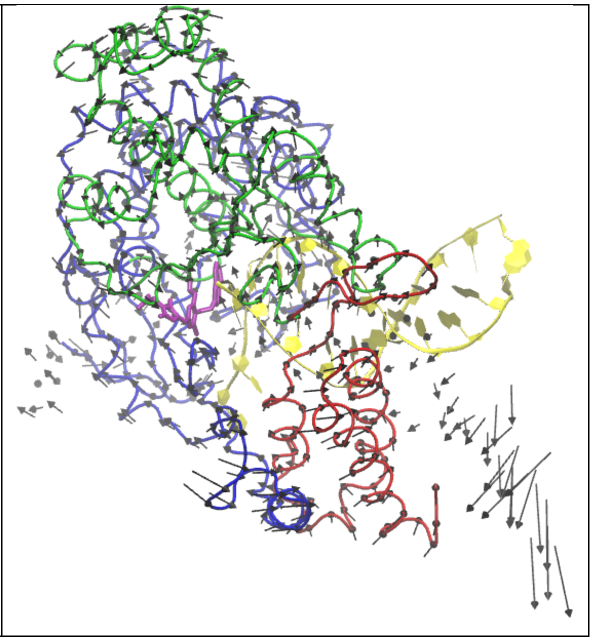

Figure S6f The top five normal modes from the ZINC000408592119 system. RNA (yellow) and Finger domain (Blue), Palm domain (Green) Thumb domain (Red) of RdRp is shown ribbon.

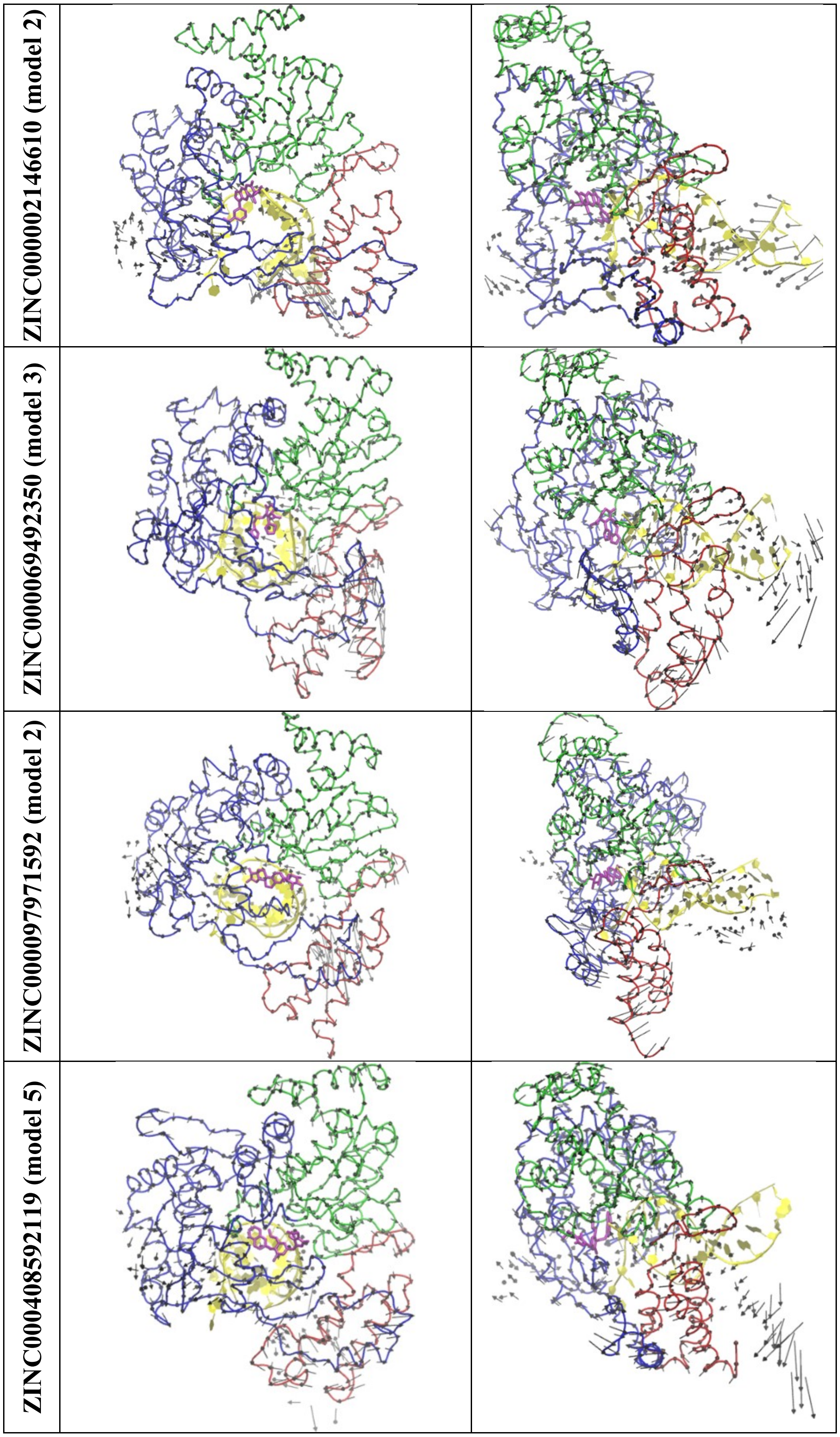

Figure S7 The top view (left) and the side view (right) of a selected normal mode from the top 4 ZINC systems that is most similar to the mode 5 of ATP system. RNA (yellow) and, Thumb domain (Red), Palm domain (Green), Finger domain (Blue) of RdRp is shown ribbon.

Table S1. Summary of top 14 ZINC compounds with corresponding Smiles ID and 2D structure.

Molecule	Smiles ID Code	
ZINC000002146610	COc1cccc(-c2coc3cc4oc(=O)c(CC(=O)O)c(C)c4cc23)c1	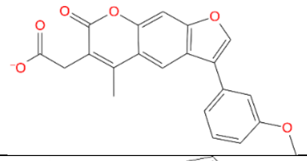
ZINC000014651456	N[C@H](Cc1ccccc1)C(=O)N[C@H](Cc1c[nH]c2ccccc12)C(=O)O	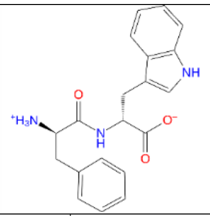
ZINC000016040970	Cc1cc(OCC(=O)N[C@H](CC(=O)O)C(=O)O)c2c3c(c(=O)oc2c1)CCC3	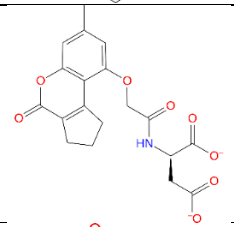
ZINC000065742965	CN(C)CC/C=C1/c2ccccc2COc2ccc([C@@H](O)C(=O)O)cc21	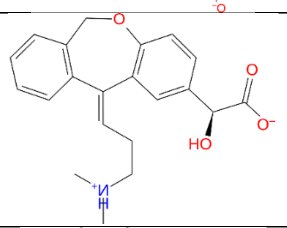
ZINC000067790716	O=C(O)c1c(S(=O)(=O)NCc2cccc3ccccc23)sc2c1CCNC2	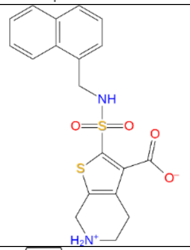
ZINC000069492350	O=C(O)[C@H]1CCC(=O)N(CCCn2cccn2)[C@H]1c1cccs1	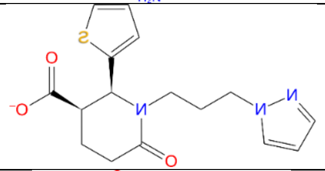
ZINC000084651559	C[C@@H](NC(=O)c1c(O)cc(F)cc1F)c1ccc(N2CCOC2=O)cc1	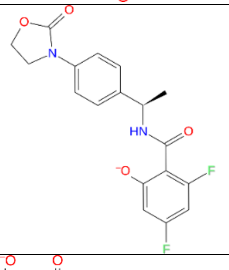
ZINC000089920955	O=C(NC[C@H]1CC(=O)N(Cc2ccccc2)C1)c1ccc(F)cc1O	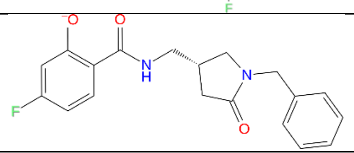

ZINC000097971592	C[C@]12CC[C@@H]3c4ccc(O[C@H]5O[C@H](C(=O)O)[C@@H](O)[C@H](O)[C@H]5O)cc4CC[C@H]3[C@@H]1C[C@@H](O)[C@@H]2O	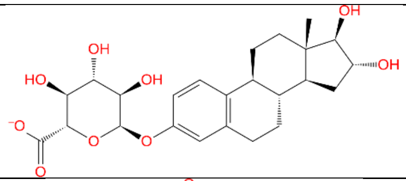
ZINC000237948681	O=C(NC1(C(=O)O)CC1)[C@H]1CC(=O)N(c2ccc(Cl)cc2)C1	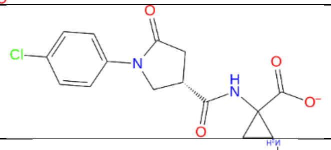
ZINC000238950253	NC(=O)c1ccc[n+]([C@@H]2O[C@H](CO[P@](=O)(O)O[P@](=O)(O)OC[C@@H]3O[C@H](n4cnc5c(N)ncnc54)[C@H](OP(=O)(O)O)[C@@H]3O)[C@@H](O)[C@H]2O)c1	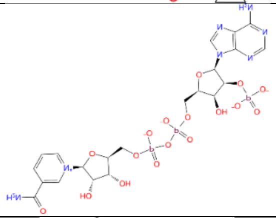
ZINC000257306096	O=C(O)c1ccccc1-c1ccc(Cn2ncccc2=O)nc1	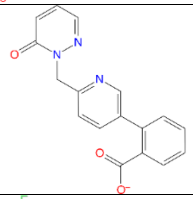
ZINC000299798705	CN(C)[C@@H]1C[C@@H](C(=O)O)N(C(=O)c2cc(-c3ccccc3F)n[nH]2)C1	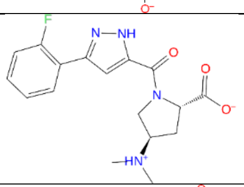
ZINC000408592119	CCOc1cc(/C=C2/NC(=O)N(CC(=O)O)C2=O)cc(Cl)c1OCc1ccc2ccccc2c1	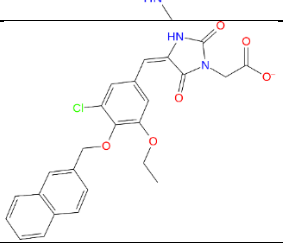

Table S2. 7BV2 RdRp-RNA-ligand complexes of ZINC compounds and zoomed perspective of the ligand docking pose with RNA and magnesium ions (left). Detailed 2D protein-ligand interaction diagram (right).

ZINC000002146610	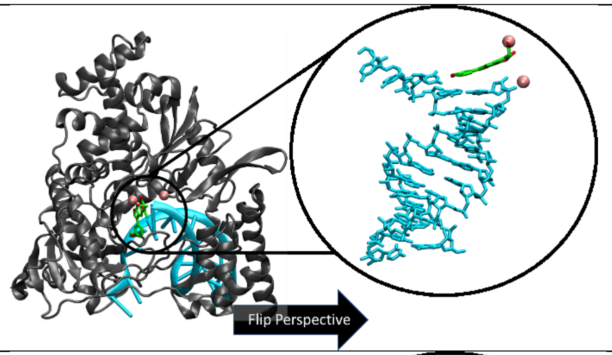	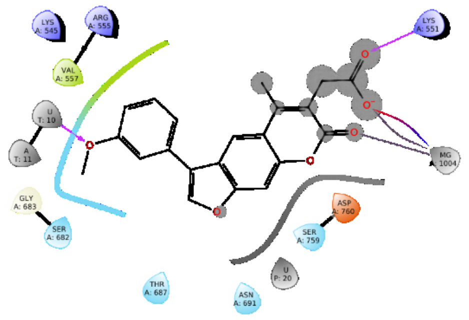
ZINC000014651456	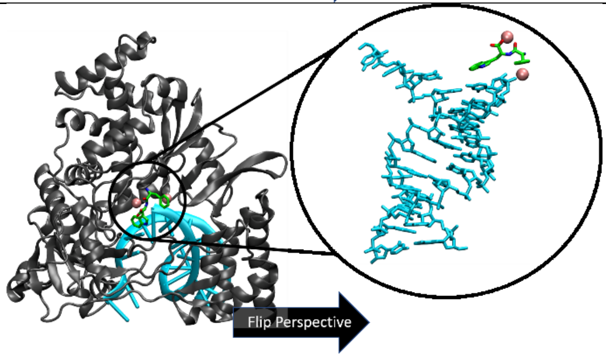	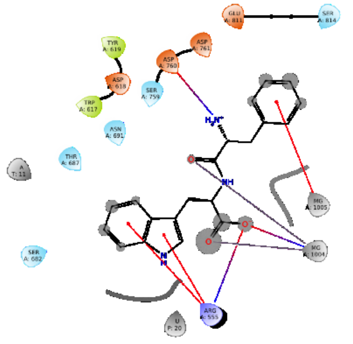
ZINC000016040970	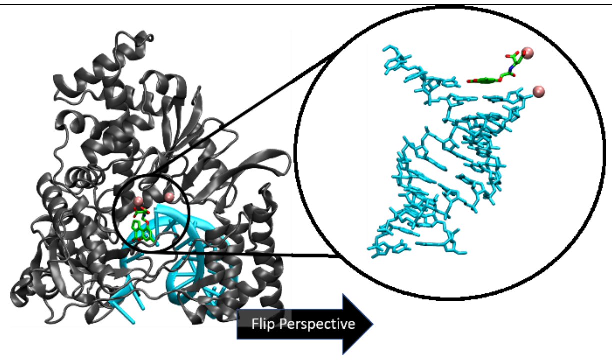	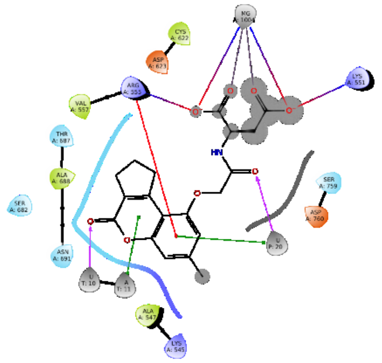
ZINC000065742965	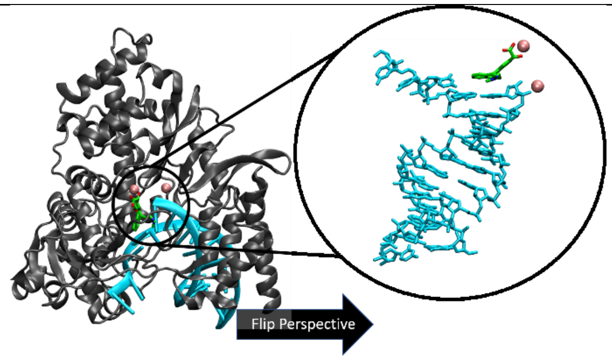	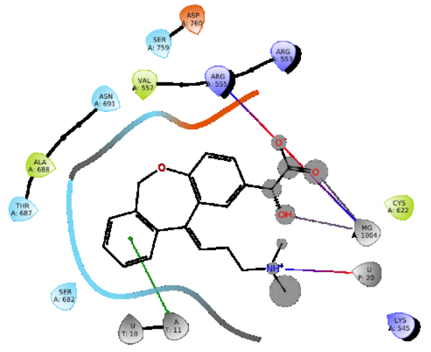

ZINC000067790716	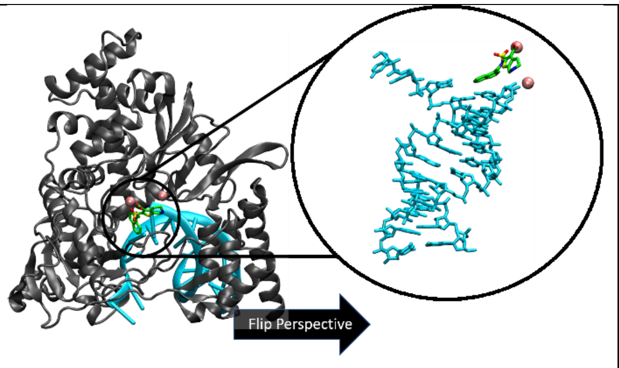	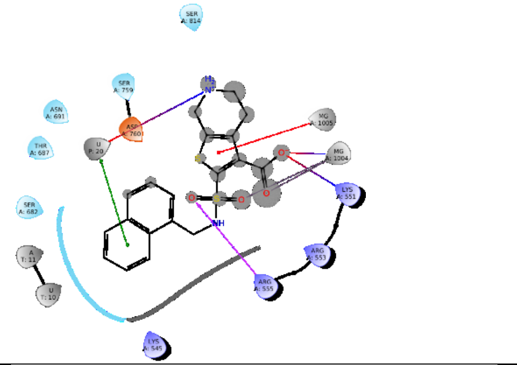
ZINC000069492350	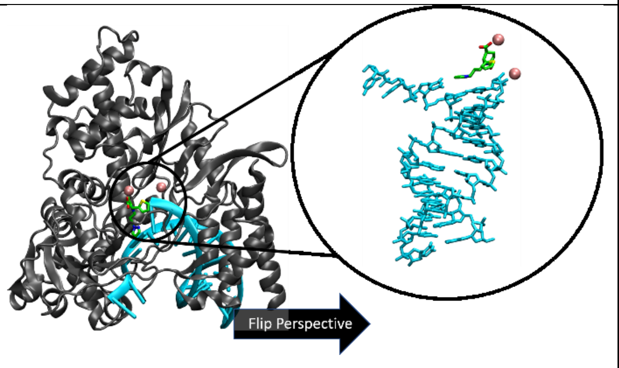	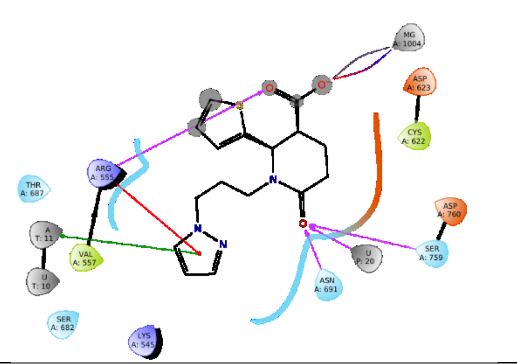
ZINC000084651559	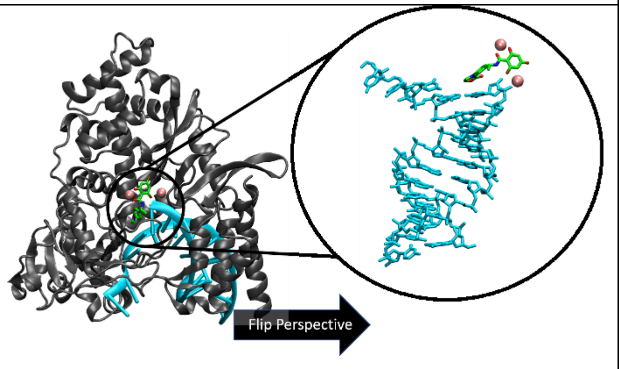	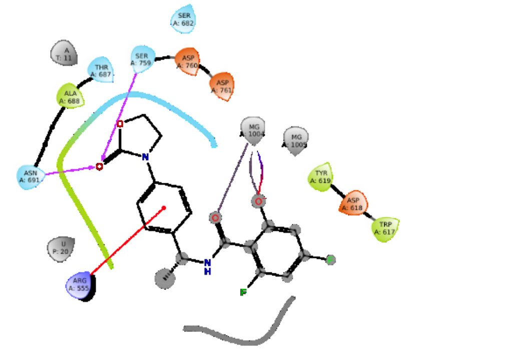
ZINC000089920955	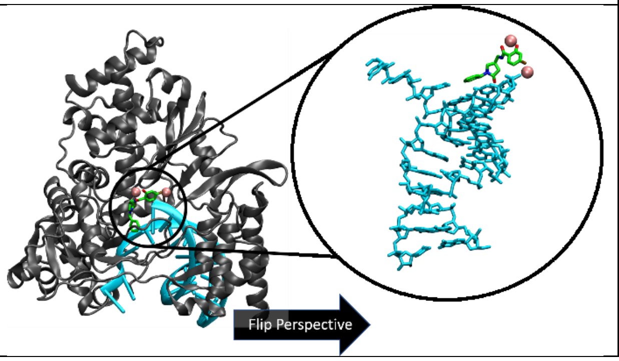	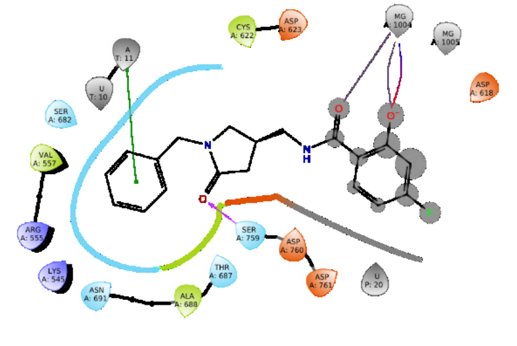

<table>
<tr><td>ZINC000097971592</td><td>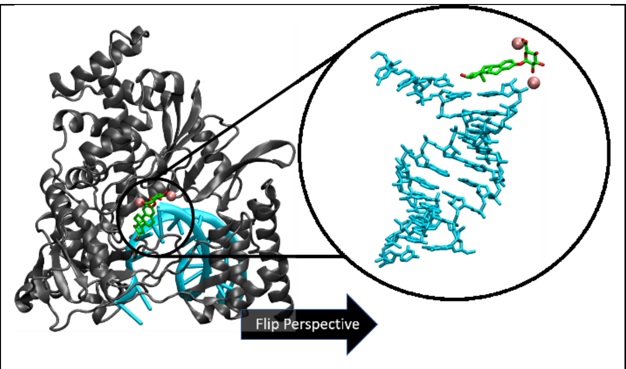
</td><td>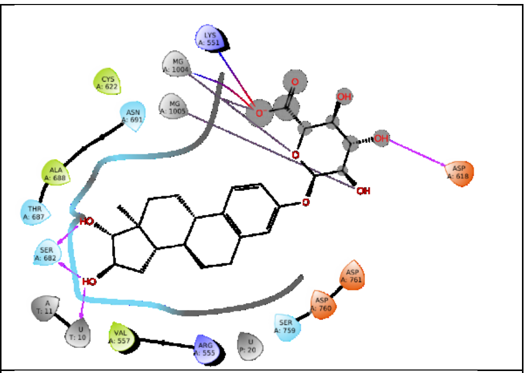</td></tr>
<tr><td>ZINC000237948681</td><td>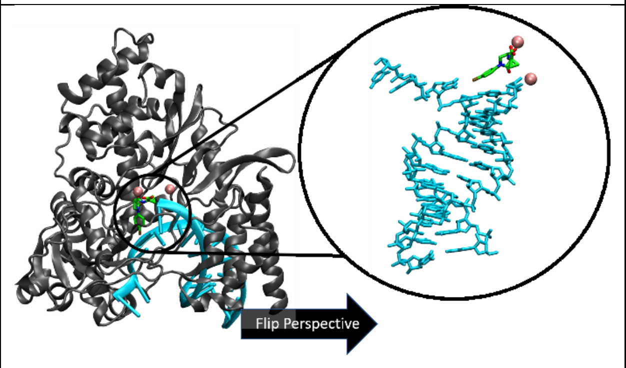
</td><td>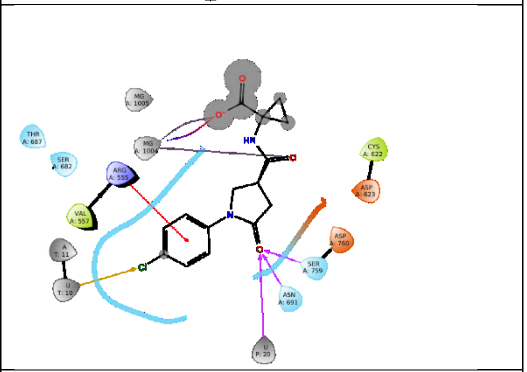</td></tr>
<tr><td>ZINC000238950253</td><td>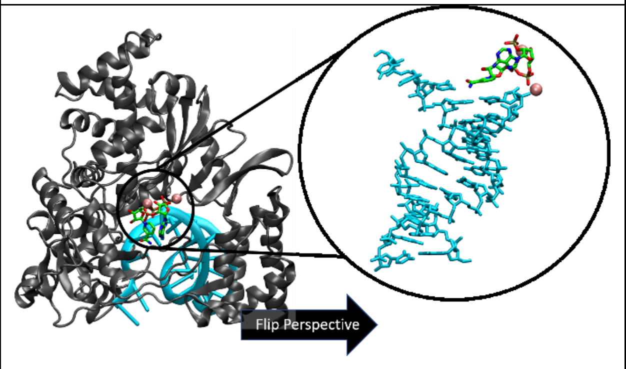
</td><td>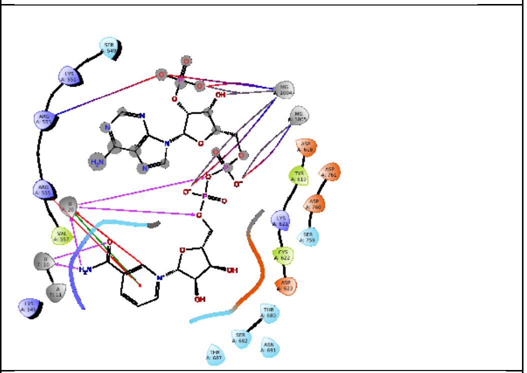</td></tr>
<tr><td>ZINC000257306096</td><td>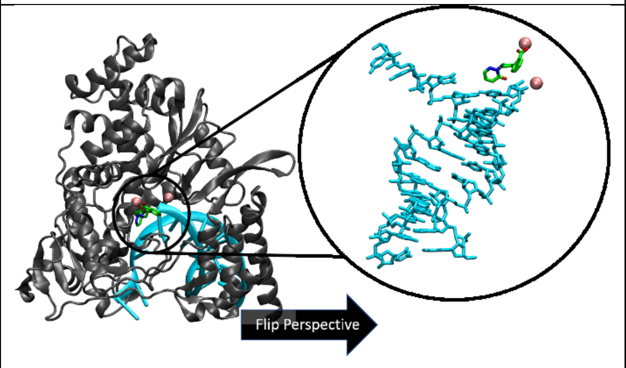
</td><td>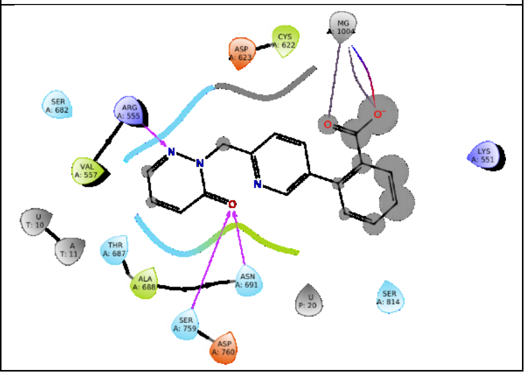</td></tr>
</table>

ZINC000299798705	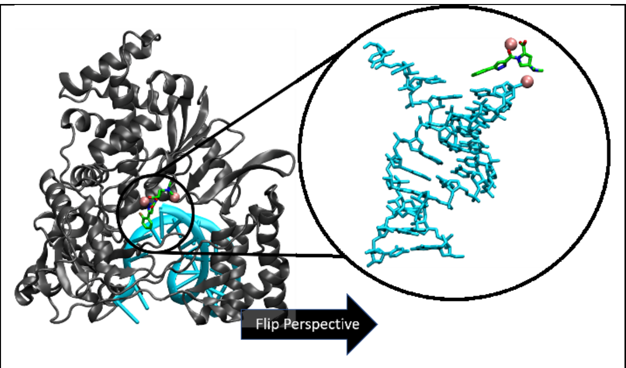	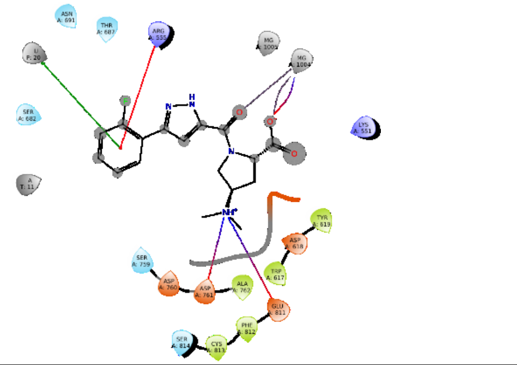
ZINC000408592119	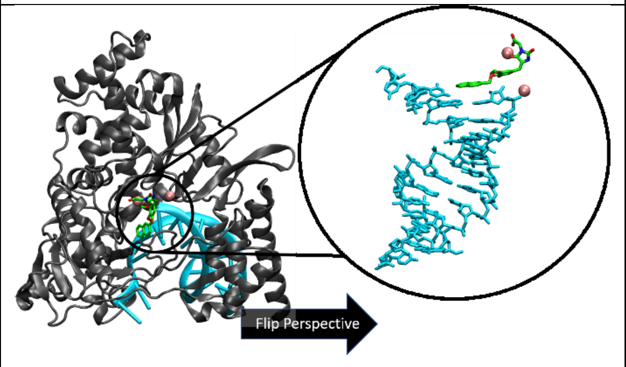	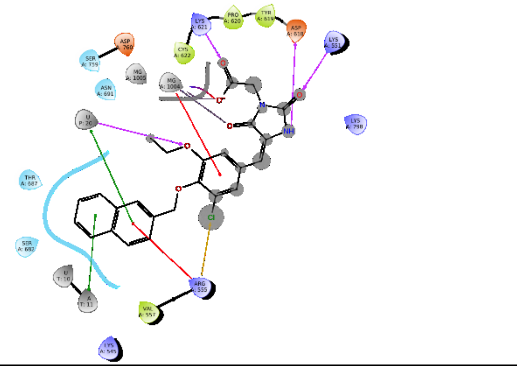

Table S3. Summary of MMGBSA calculations with standard deviations and MD simulation results.

**Ligand**	**MM-GBSA (kcal/mol)**	**Receptor RMSD (Å)**	**Ligand RMSD (Å)**
ATP	-35.9 ± 3.1	2.4	4.5
RTP	-21.3 ± 5.9	1.9	0.9
**ZINC000002146610**	**-32.4 ± 5.1**	**3.2**	**1.2**
ZINC000014651456	-23.7 ± 5.4	3.4	2.5
ZINC000016040970	-22.6 ± 13.4	2.9	3.0
ZINC000065742965	-27.3 ± 6.9	3.5	4.0
ZINC000067790716	-23.5 ± 5.2	3.3	3.6
**ZINC000069492350**	**-43.8 ± 4.1**	**4.6**	**3.7**
ZINC000084651559	-14.4 ± 6.6	3.4	3.6
ZINC000089920955	-16.4 ± 7.2	2.9	2.0
**ZINC000097971592**	**-37.4 ± 8.1**	**3.6**	**3.0**
ZINC000237948681	-28 ± 5.5	3.6	3.1
ZINC000238950253	-12.9 ± 23.9	3.1	3.0
ZINC000257306096	-23.4 ± 6.4	3.0	3.1
ZINC000299798705	-13.2 ± 7.5	2.7	5.3
**ZINC000408592119**	**-31.4 ± 4.9**	**4.3**	**3.5**

The simulation for the four bold systems has been extended from 200 ns to 1000ns.

Table S4. Receptor and ligand RMSD for top 14 ZINC candidate systems.

ZINC000002146610<br>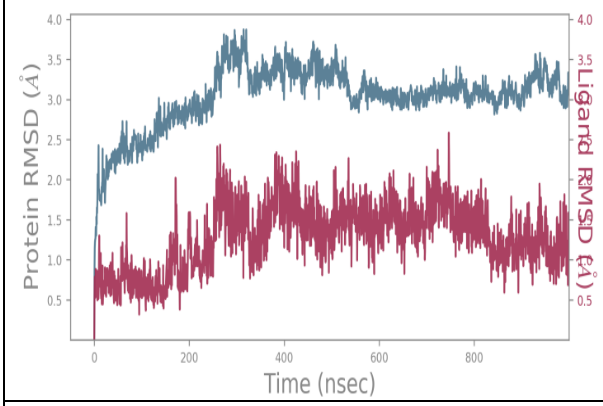	ZINC000014651456<br>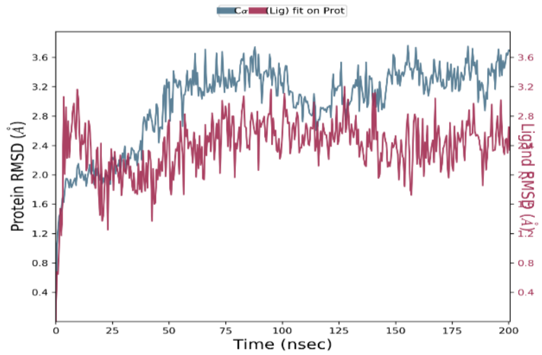
ZINC000016040970<br>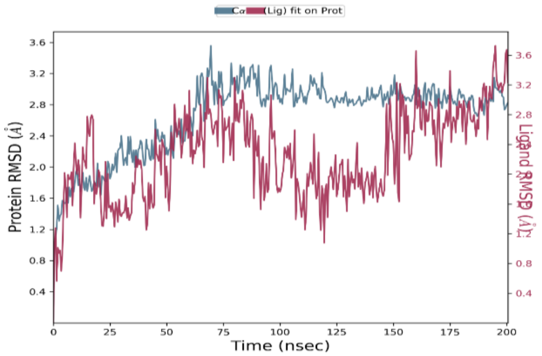	ZINC000065742965<br>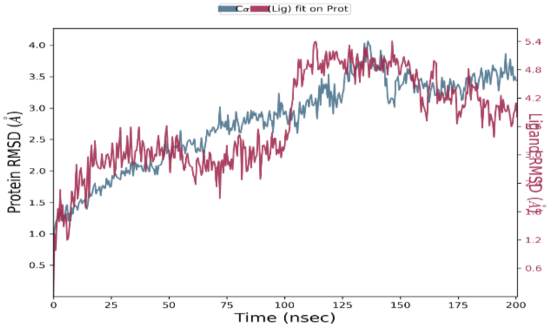
ZINC000067790716<br>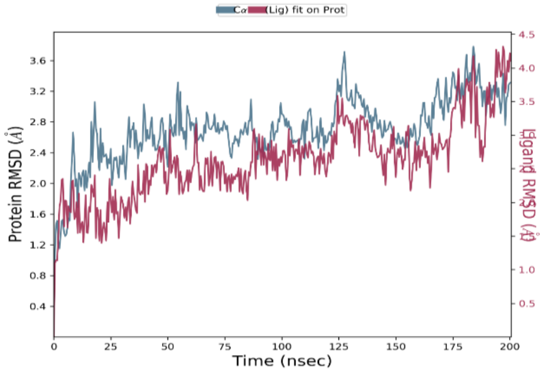	ZINC000069492350<br>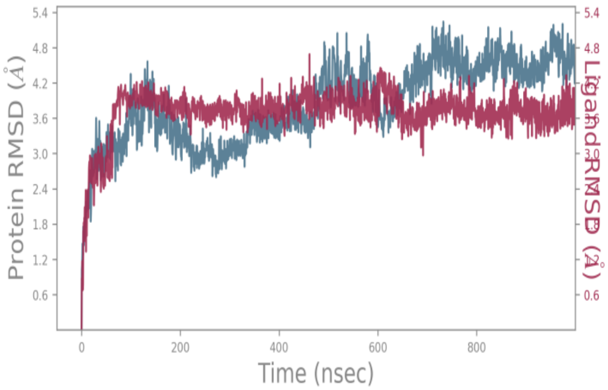
ZINC000084651559<br>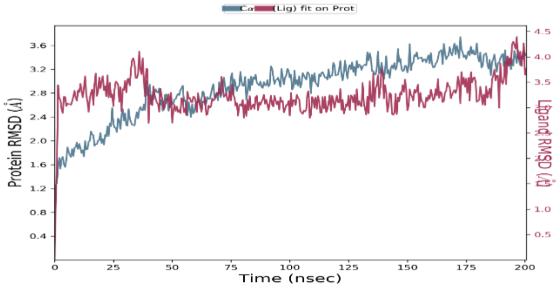	ZINC000089920955<br>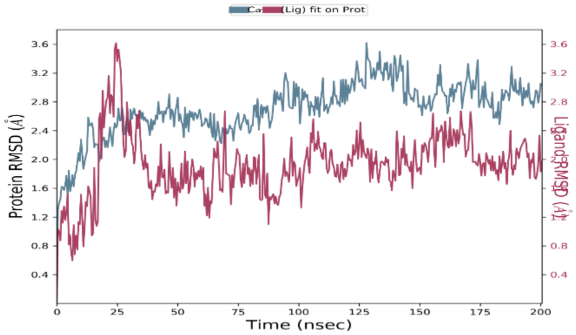

ZINC000097971592

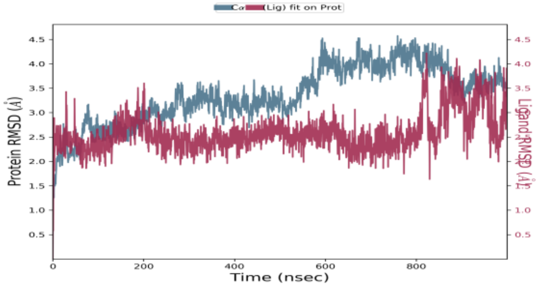

ZINC000237948681

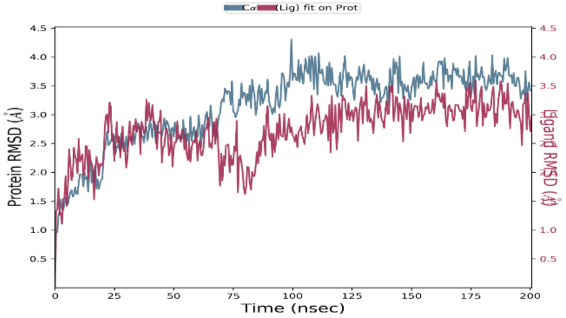

ZINC000238950253

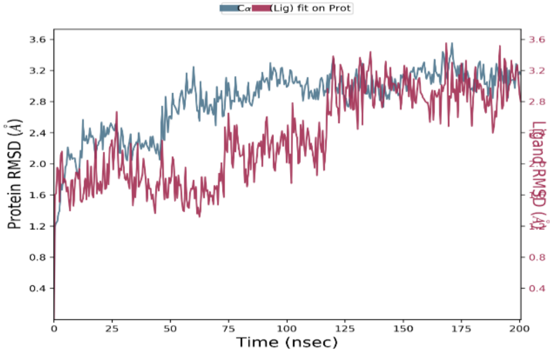

ZINC000257306096

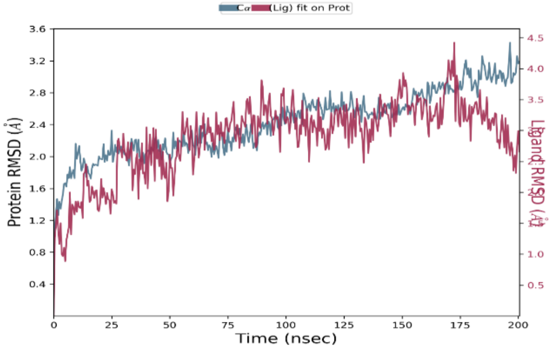

ZINC000299798705

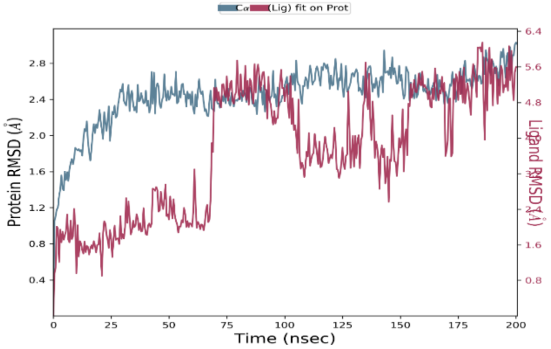

ZINC000408592119

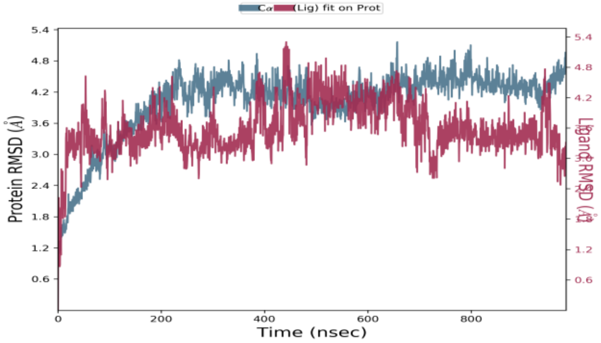

Table S5. Protein (left) and ligand (right) RMSF for top 14 ZINC candidate systems over full 200 ns simulation time.

ZINC000002146610

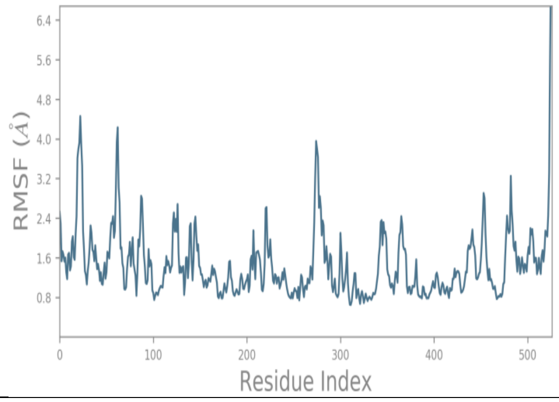

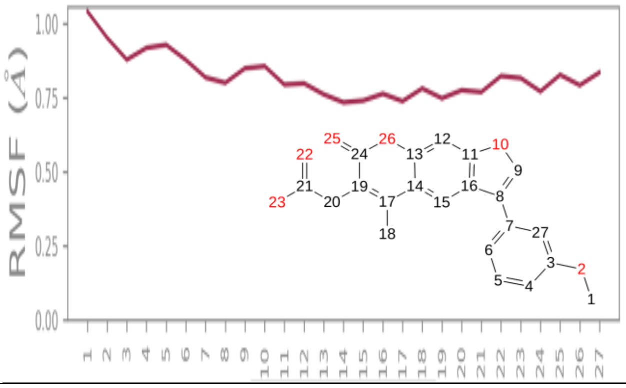

ZINC000014651456

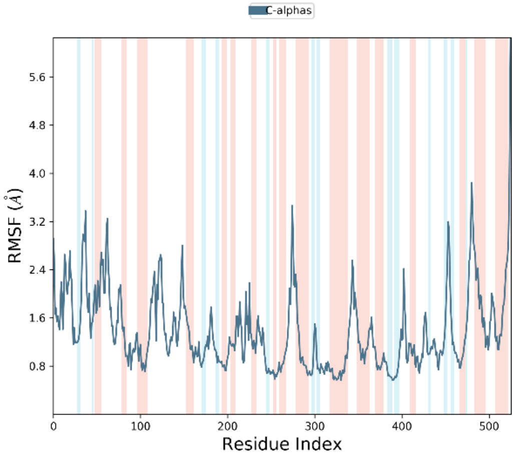

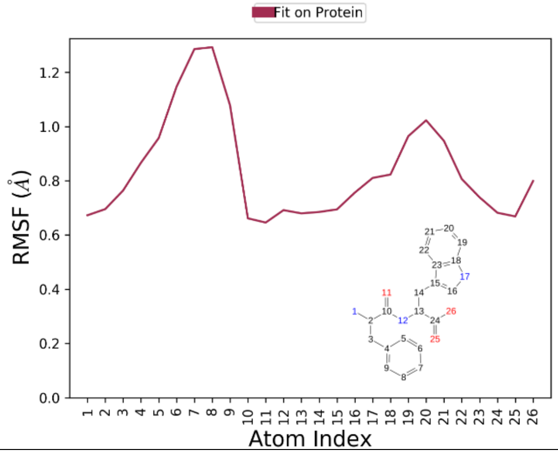

ZINC000016040970

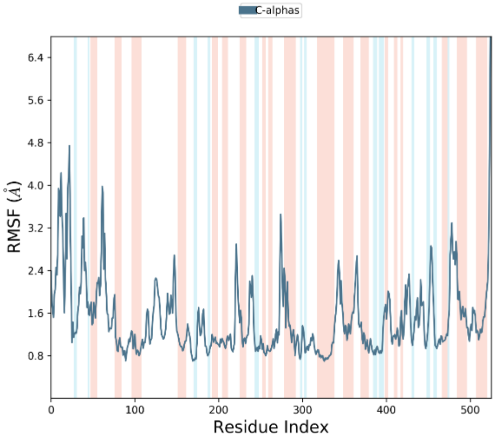

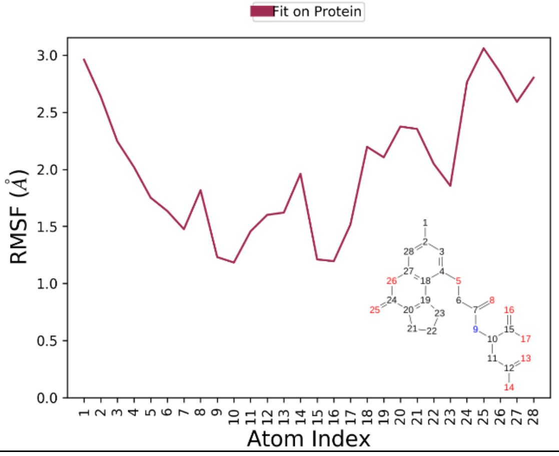

ZINC000065742965

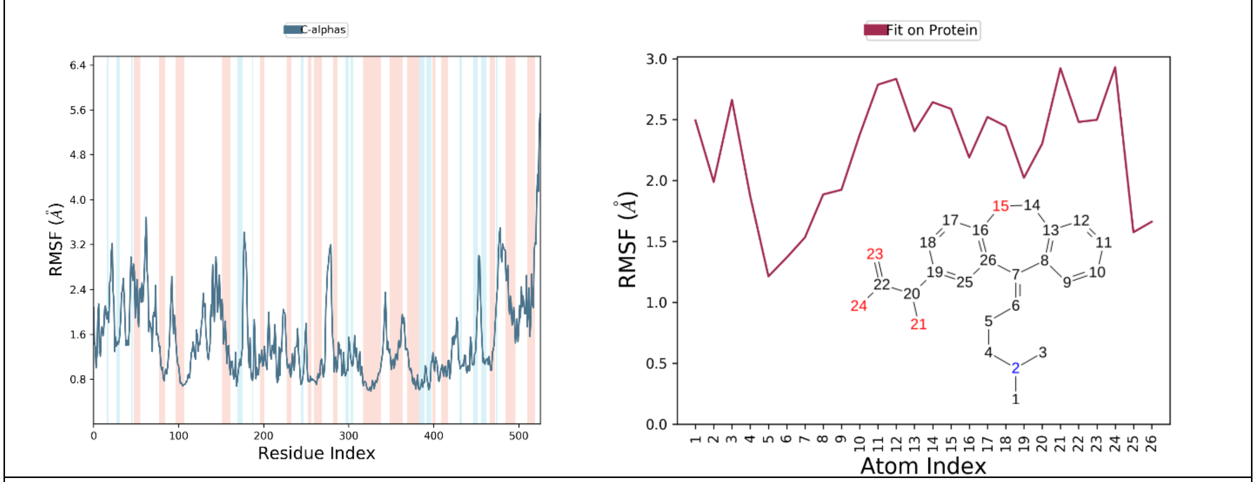

ZINC000067790716

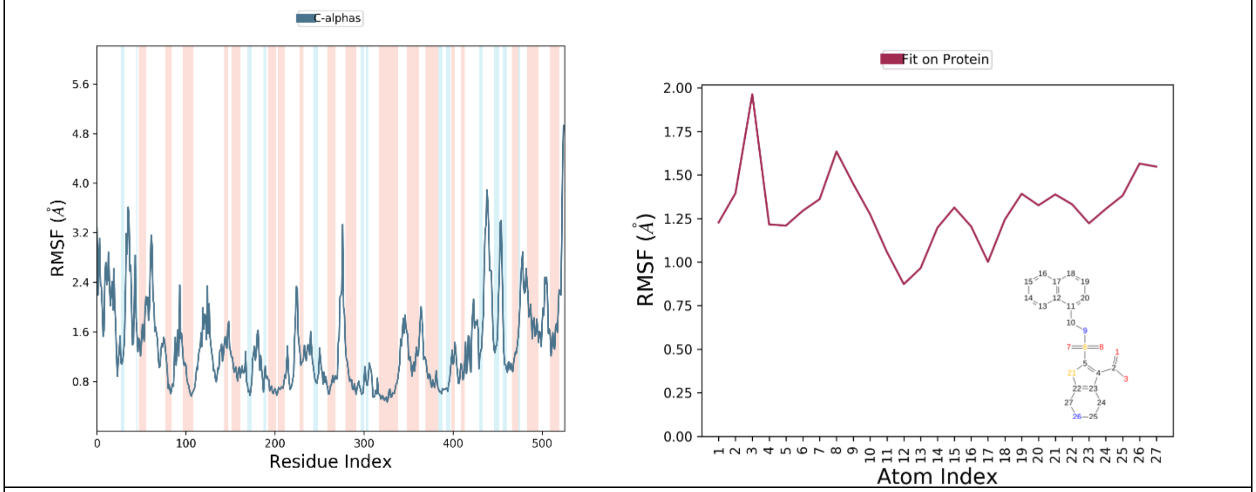

ZINC000069492350

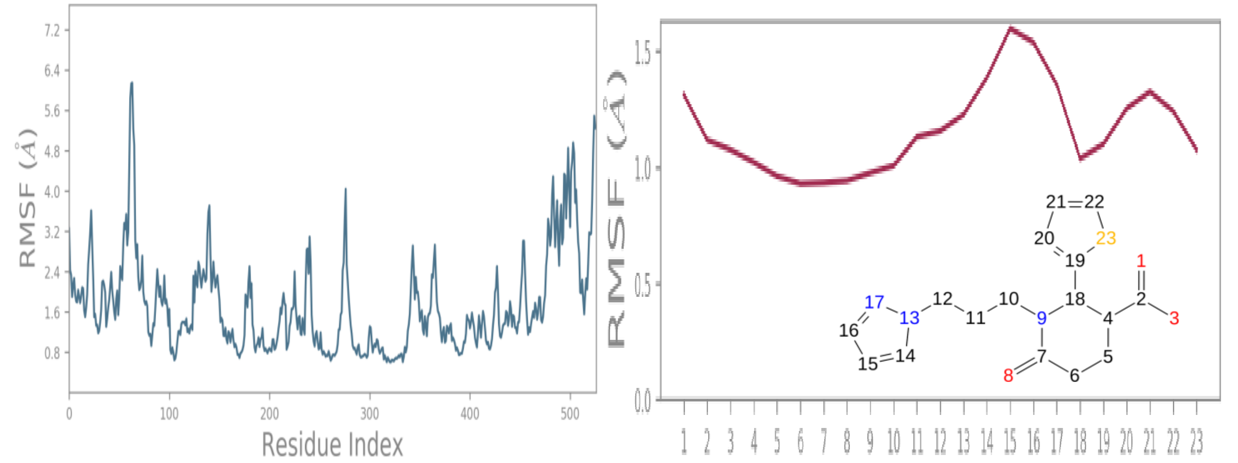

ZINC000084651559

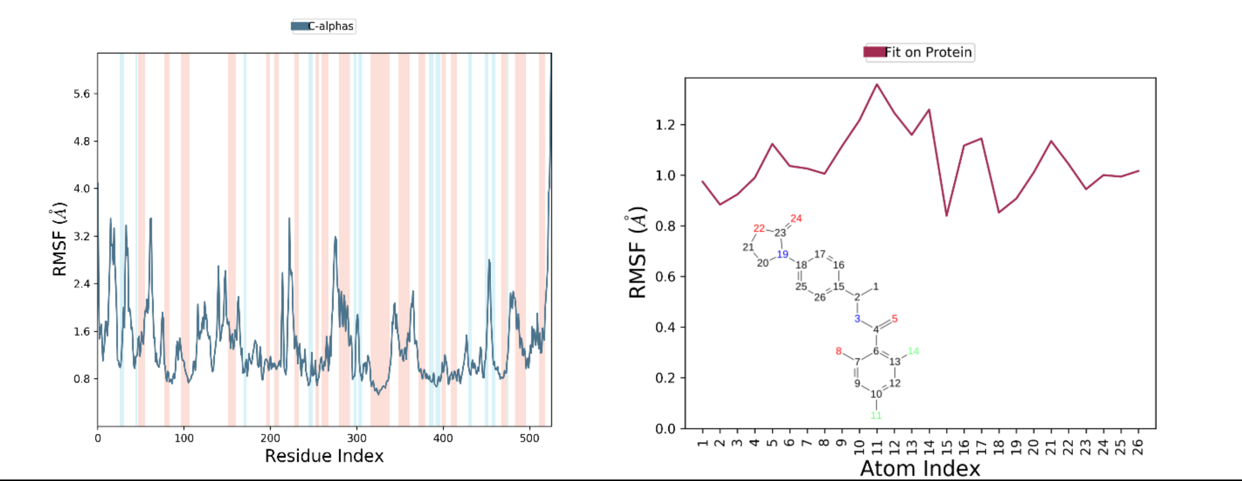

ZINC000089920955

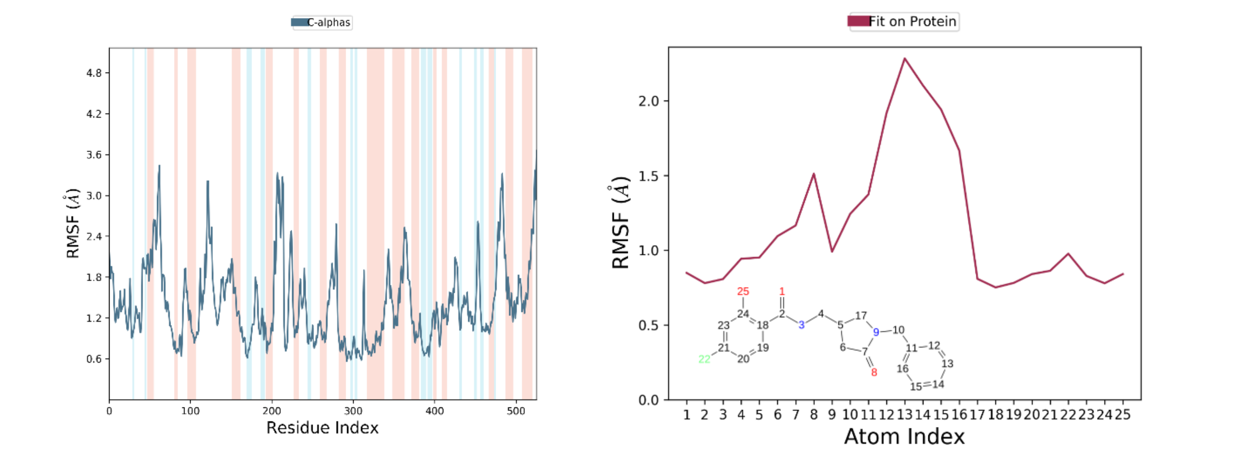

ZINC000097971592

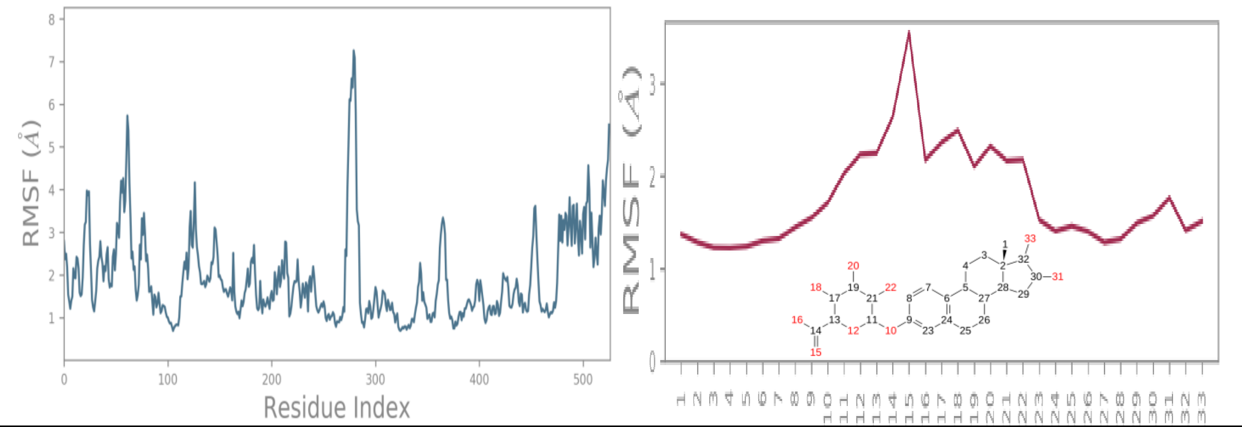

ZINC000237948681

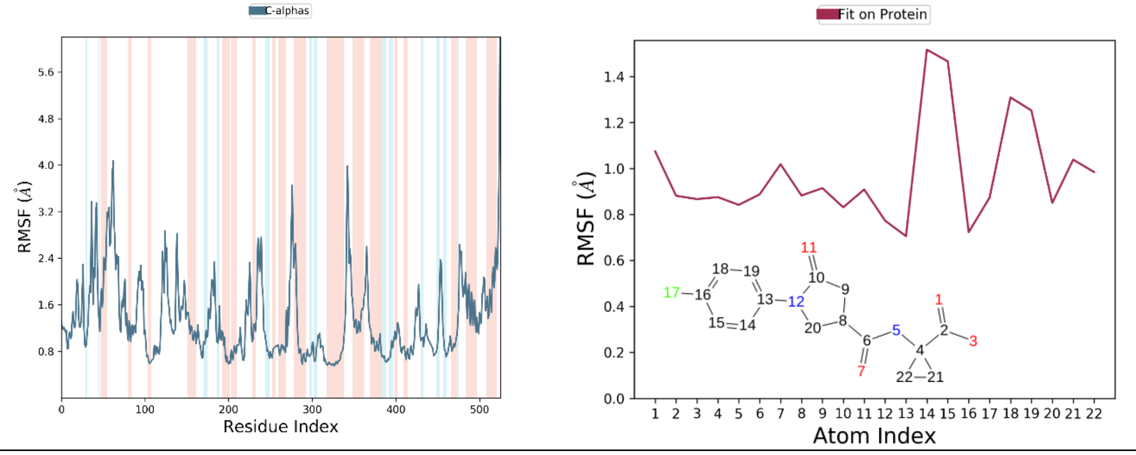

ZINC000238950253

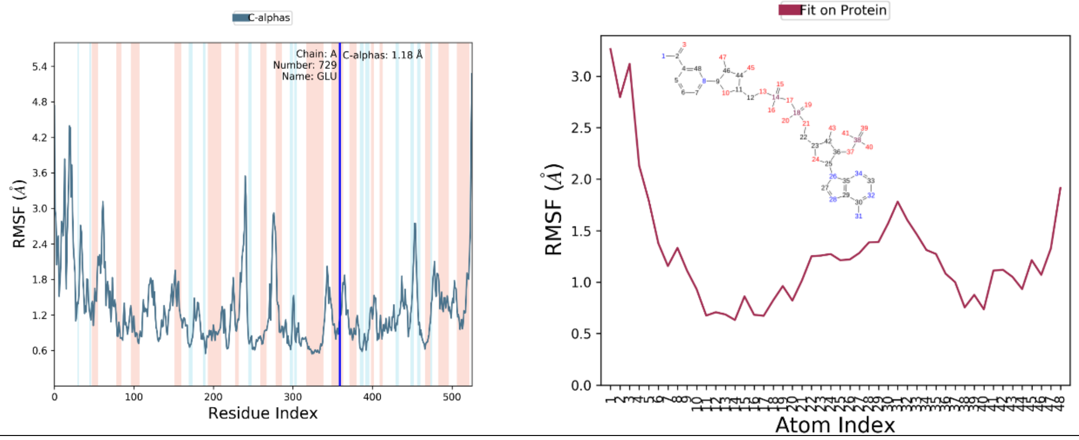

ZINC000257306096

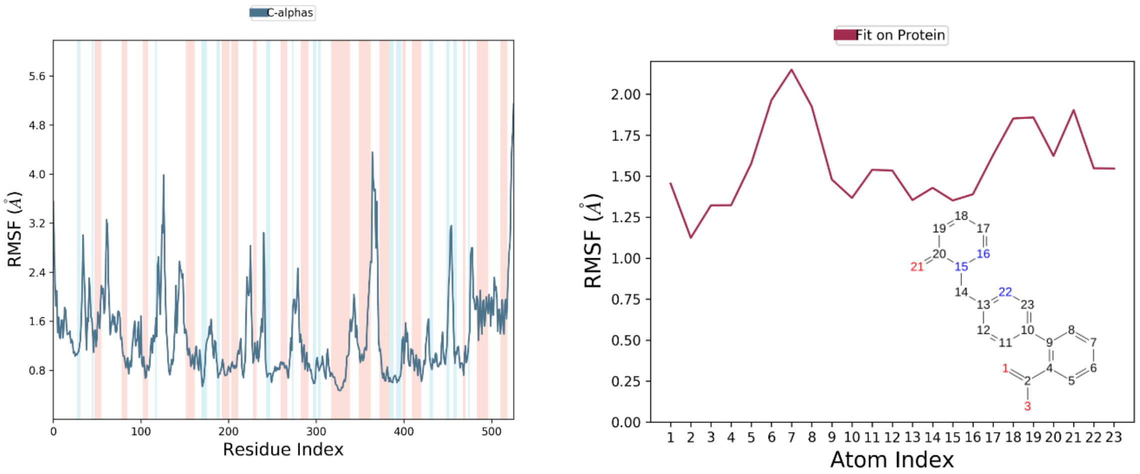

ZINC000299798705

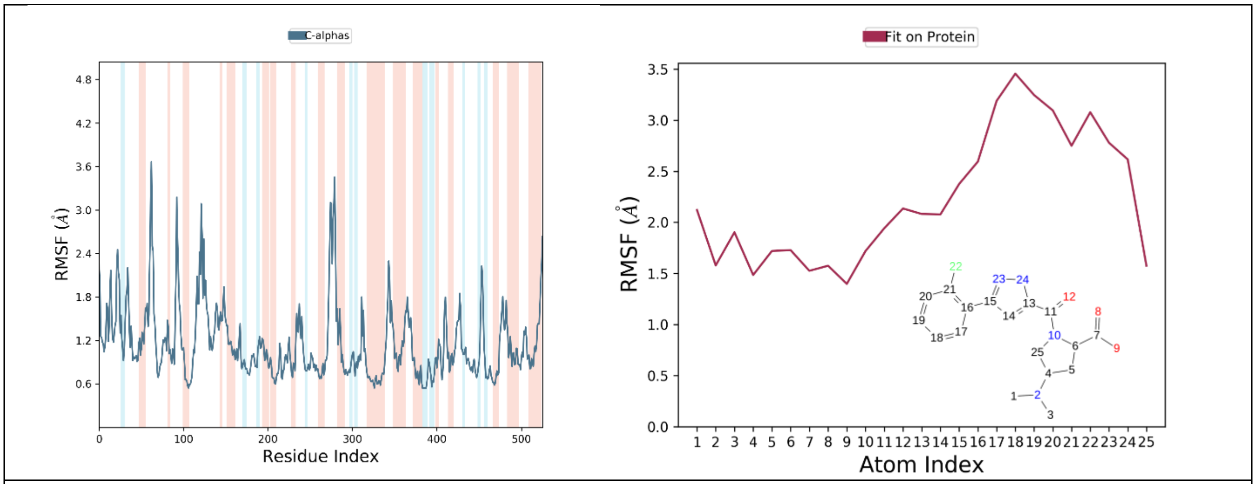

ZINC000408592119

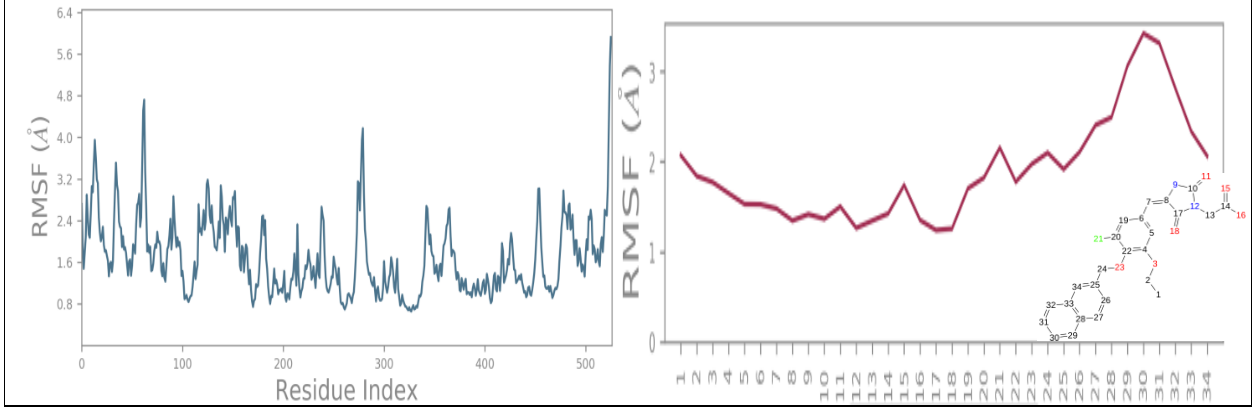

Table S6. Protein-ligand contact histogram of the top 14 candidate systems with ATP and RTP systems.

<table>
<tr><td>ATP</td><td>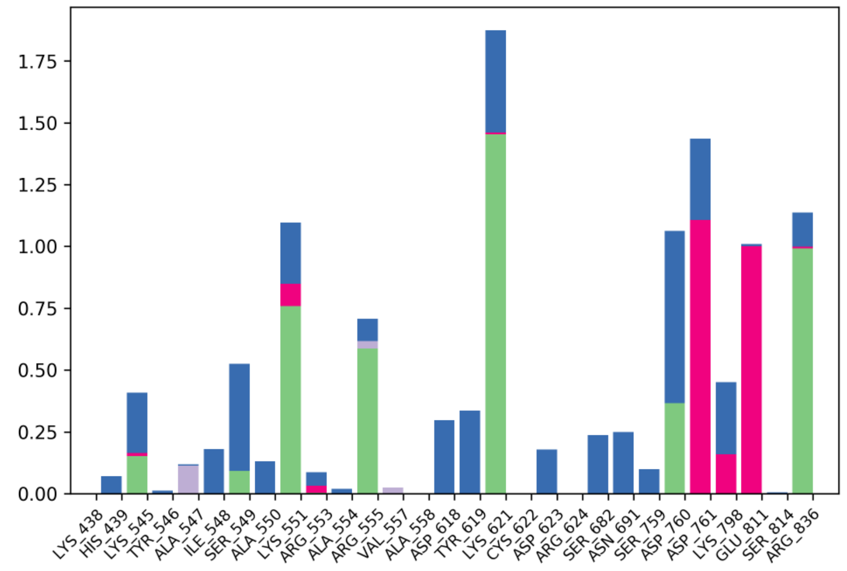
</td></tr>
<tr><td>RTP</td><td>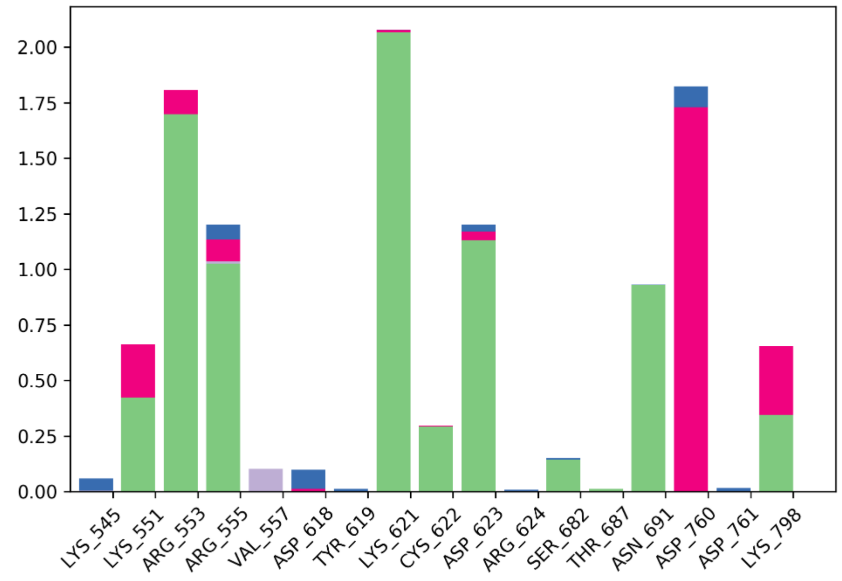
</td></tr>
</table>

ZINC000002146610	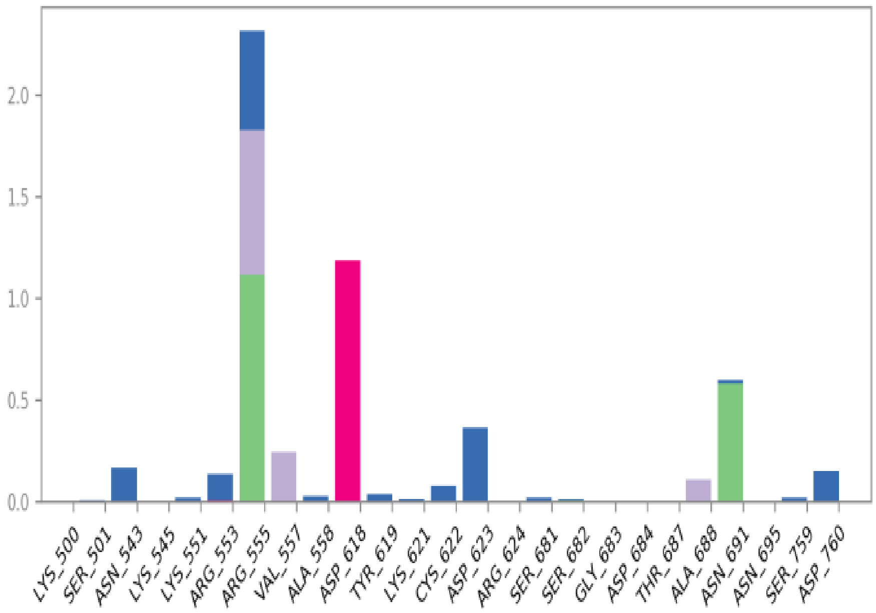
ZINC000014651456	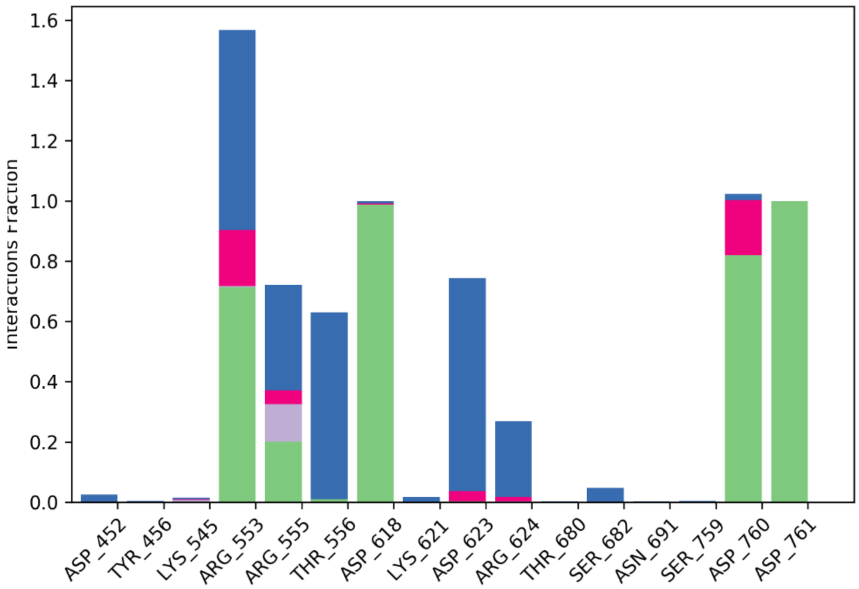

ZINC000016040970	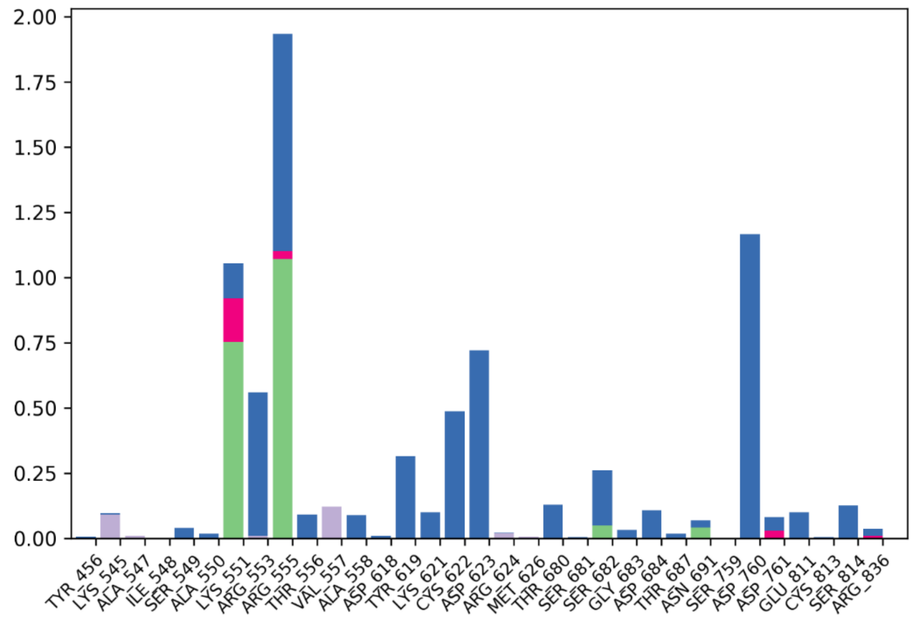
ZINC000065742965	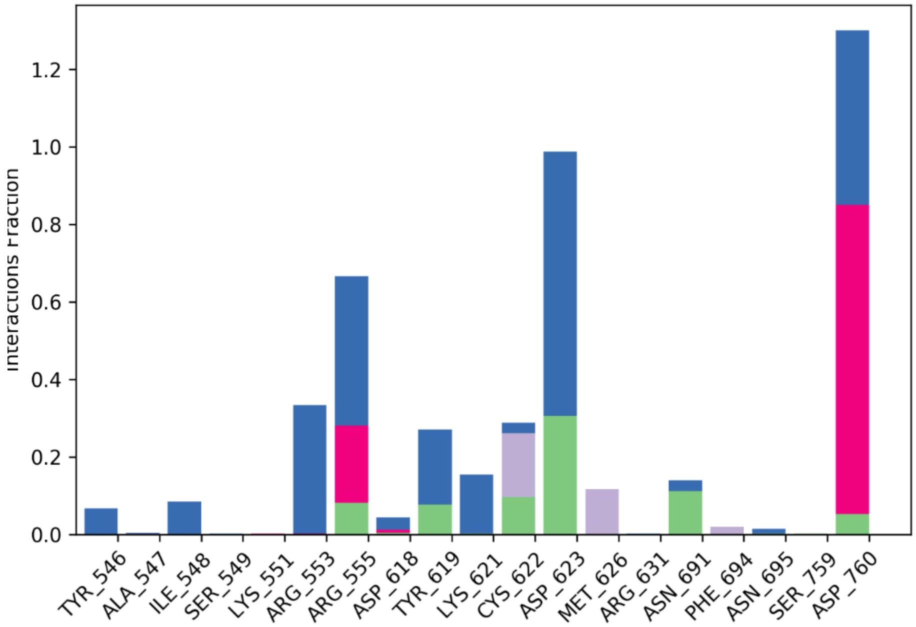

ZINC000067790716

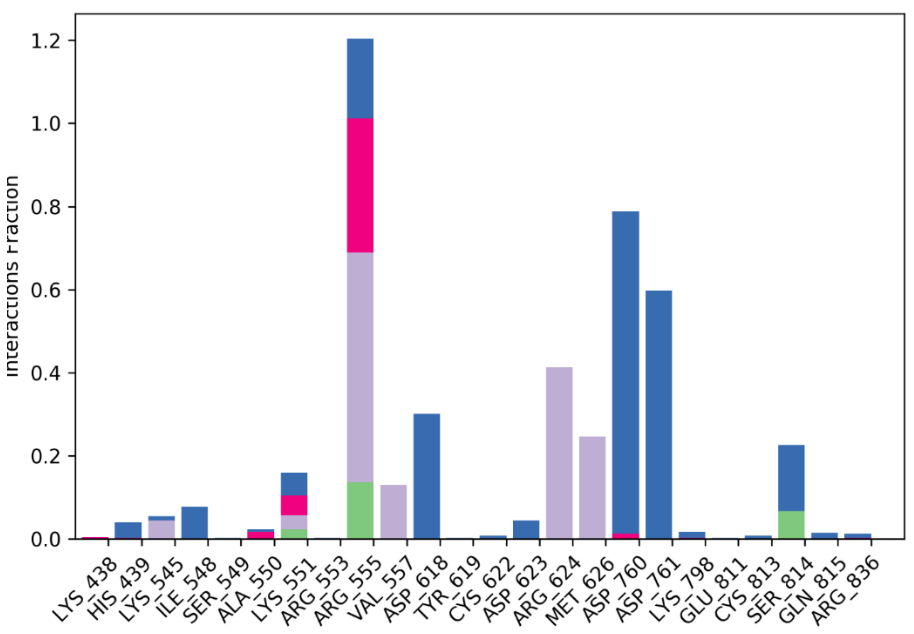

ZINC000069492350

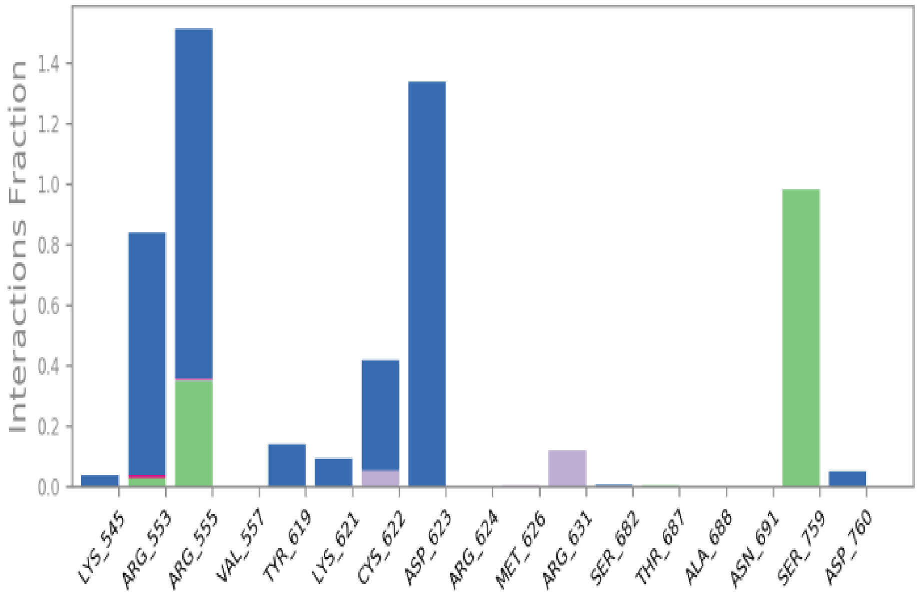

ZINC000084651559

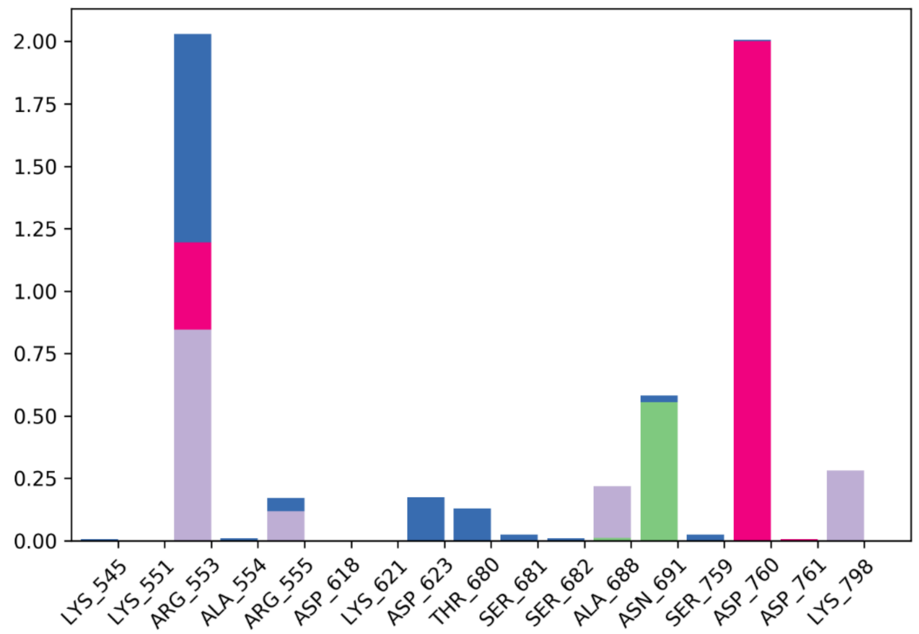

ZINC000089920955

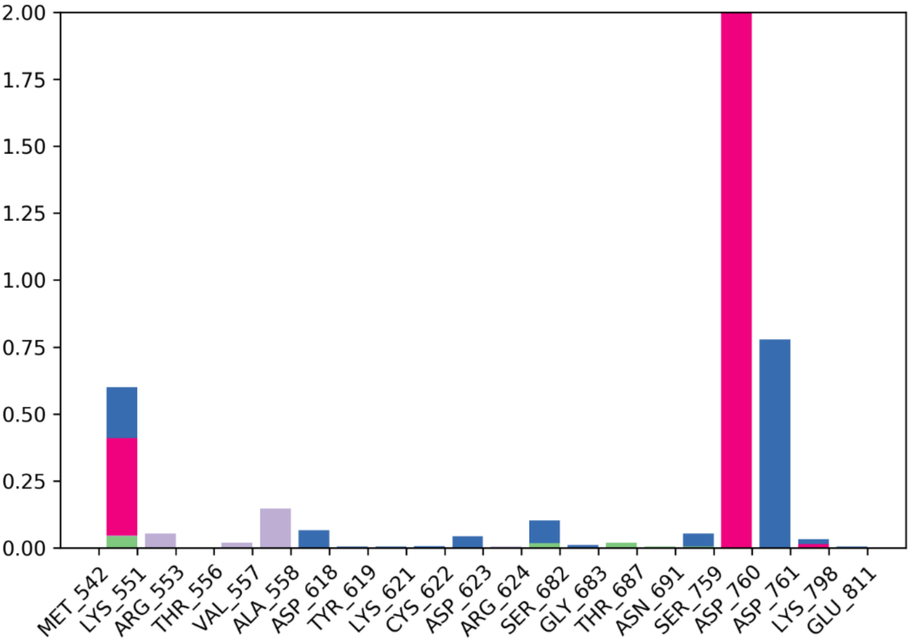

ZINC000097971592

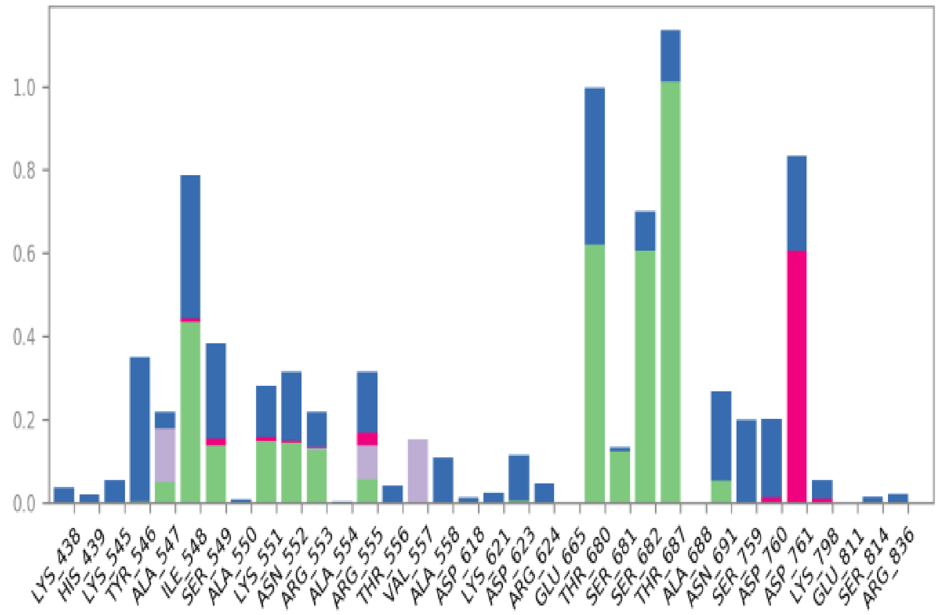

ZINC000237948681

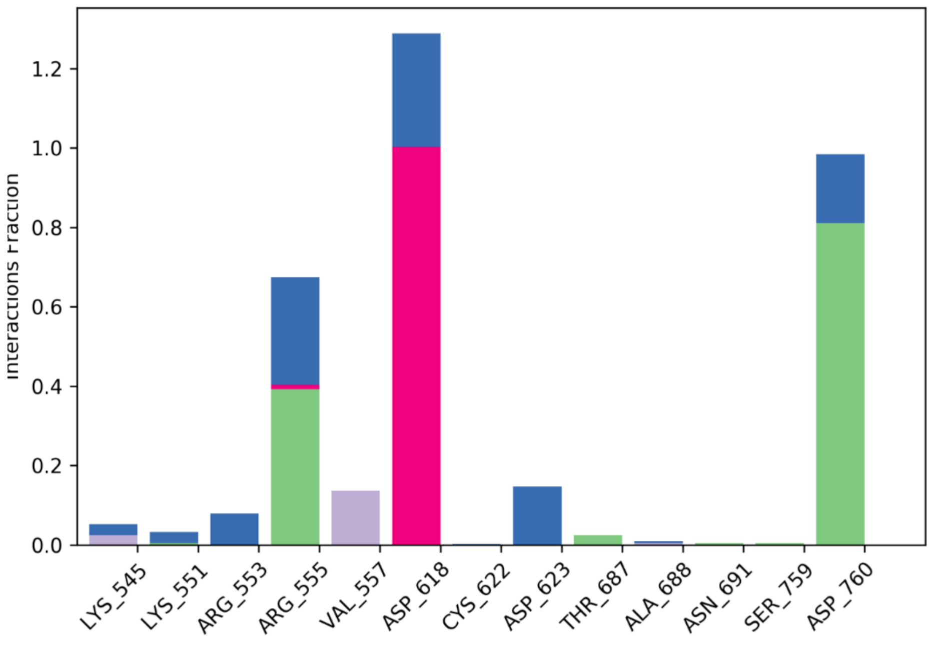

ZINC000238950253

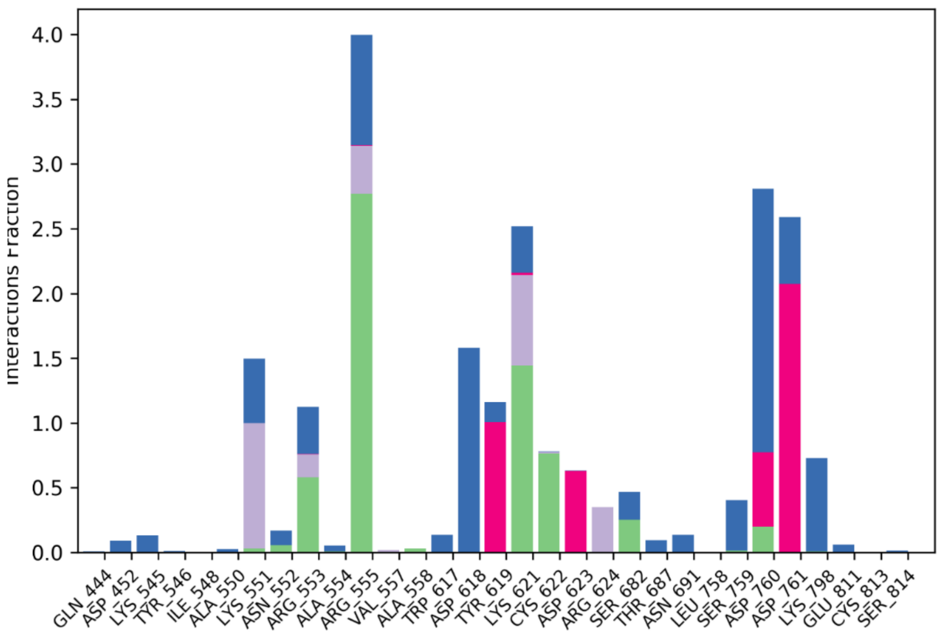

ZINC000257306096

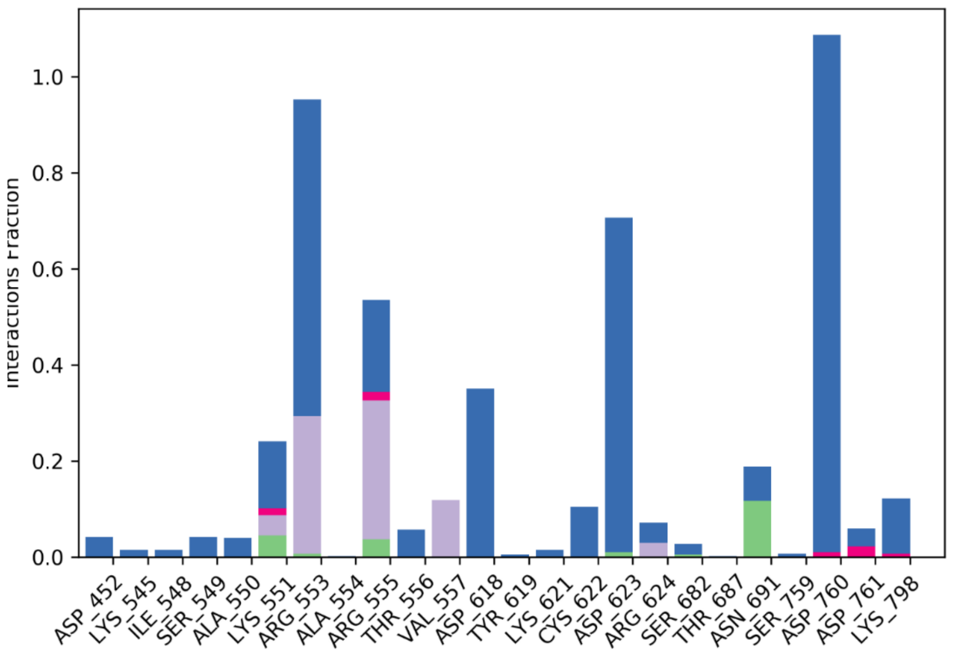

ZINC000299798705

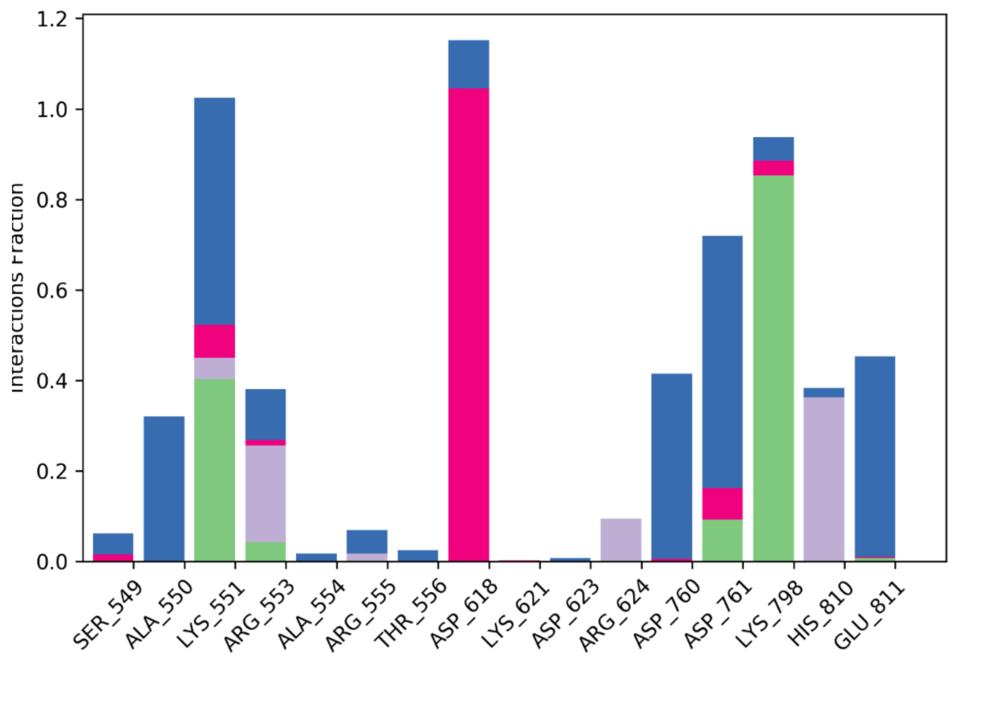

ZINC000408592119

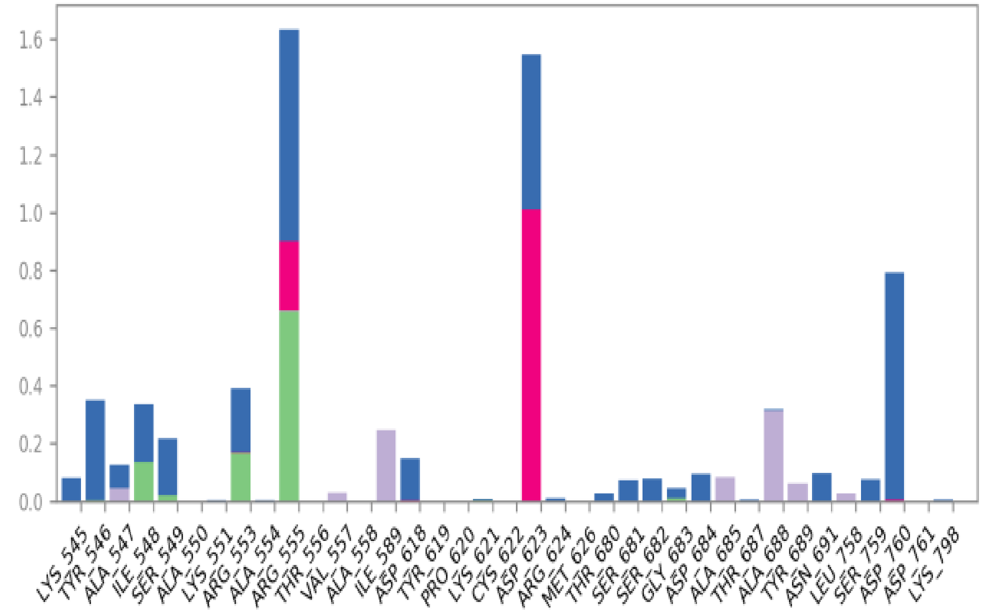

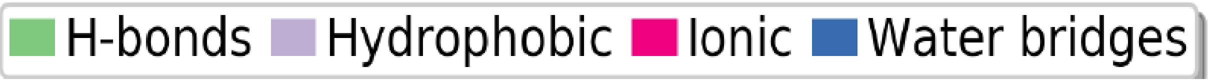

Table S7. Detailed diagram showing interactions between ligand atoms and protein residues during the simulation. Ligand-protein contacts shown occur 30% or more of the trajectory.

ATP

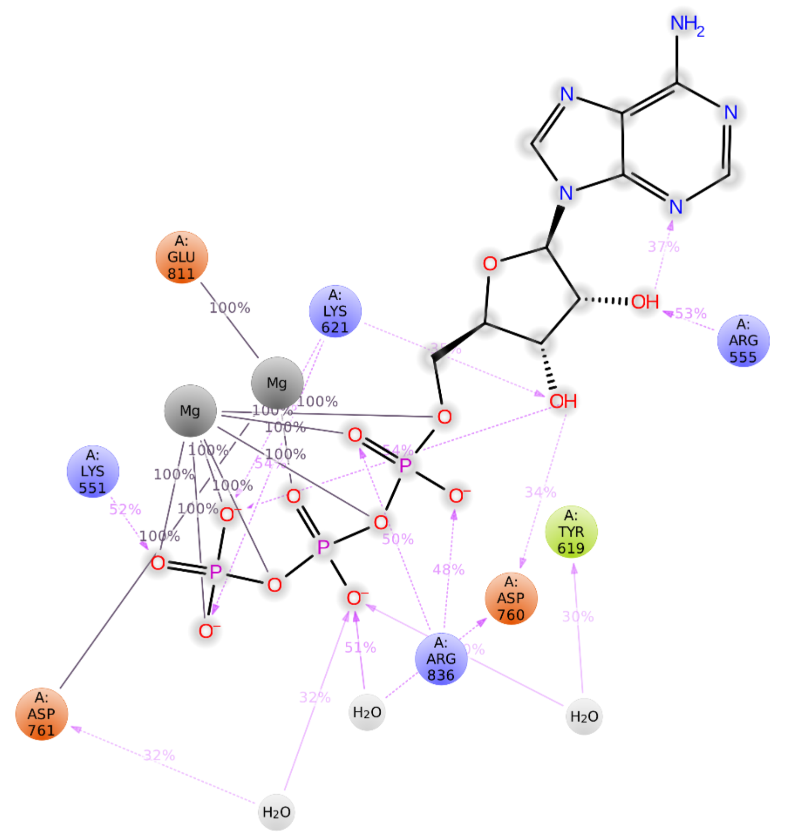

RTP

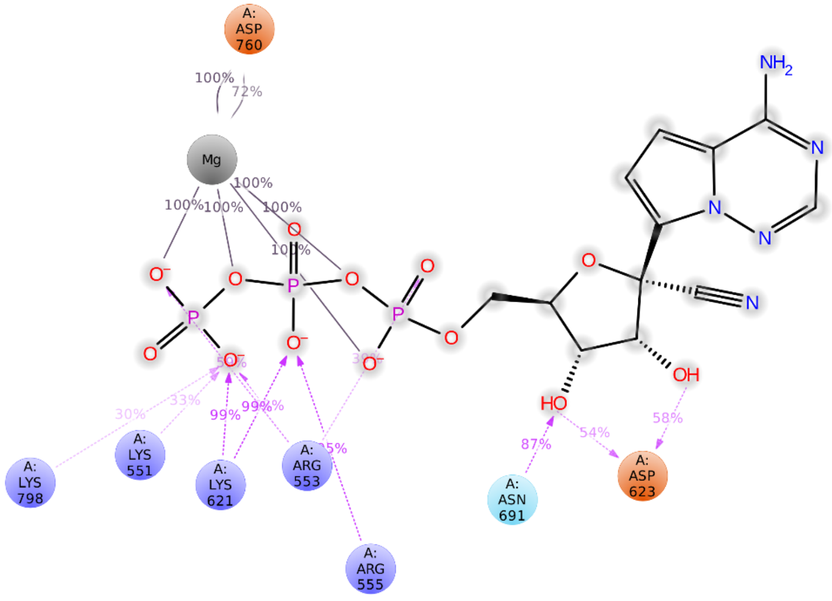

ZINC000002146610_2

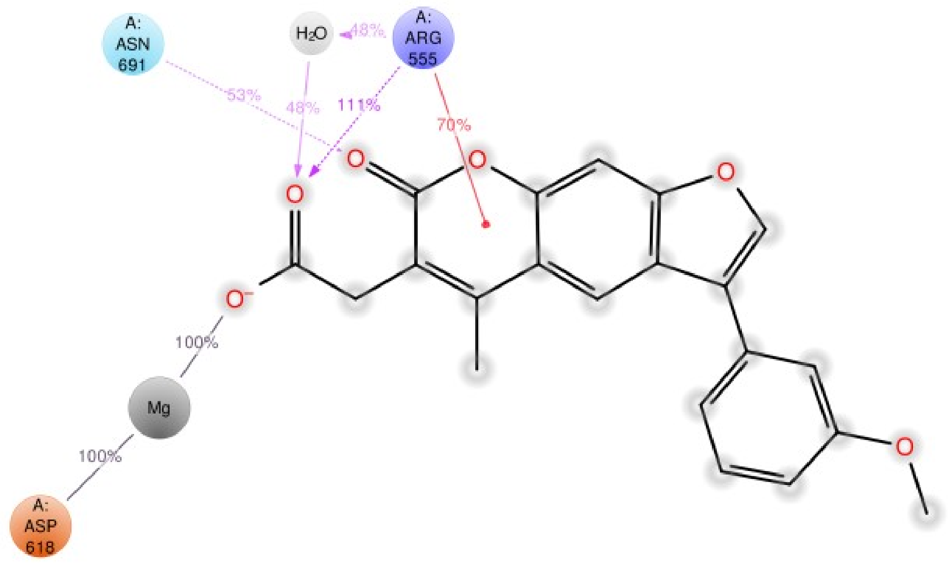

ZINC000014651456

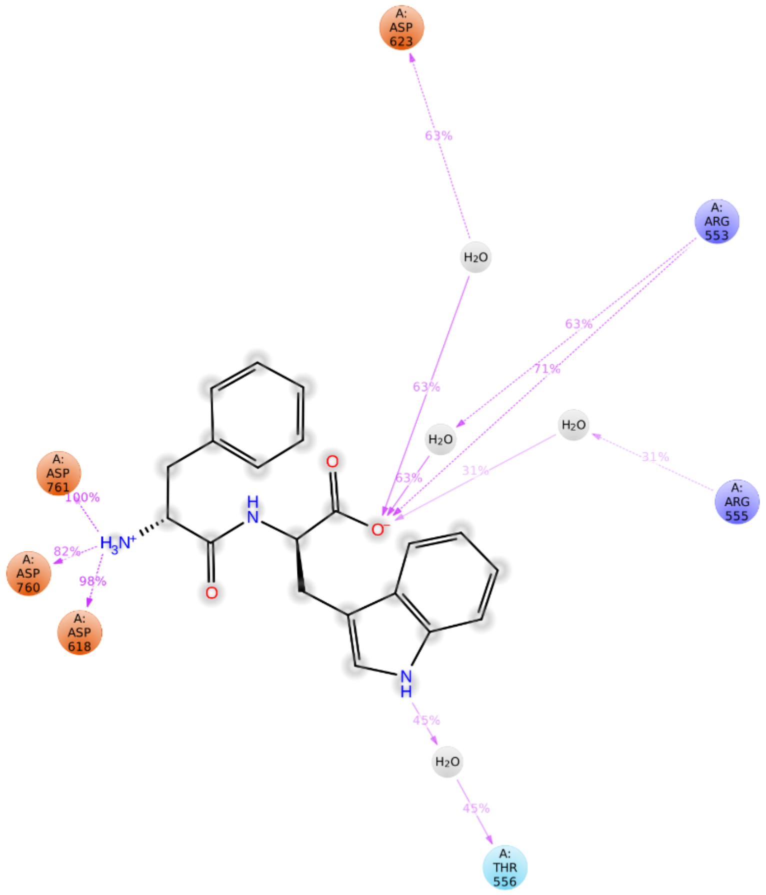

ZINC000016040970

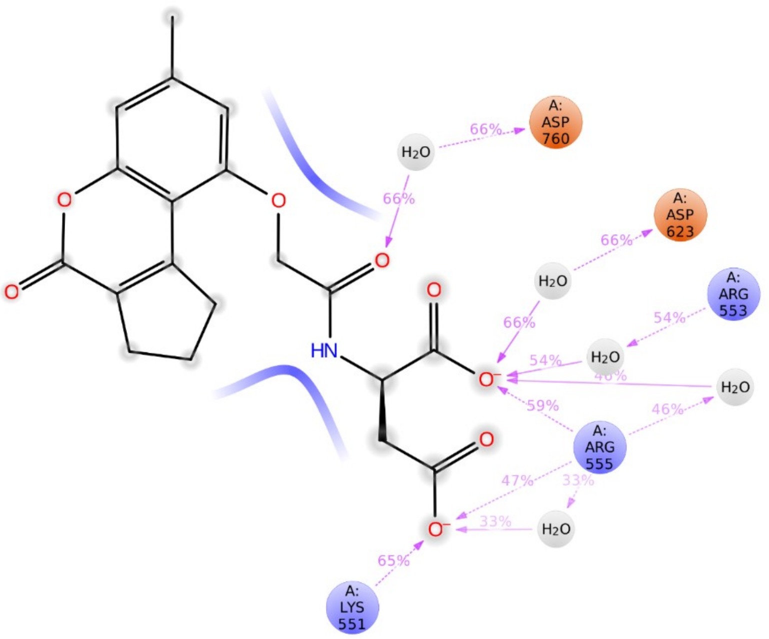

ZINC000065742965

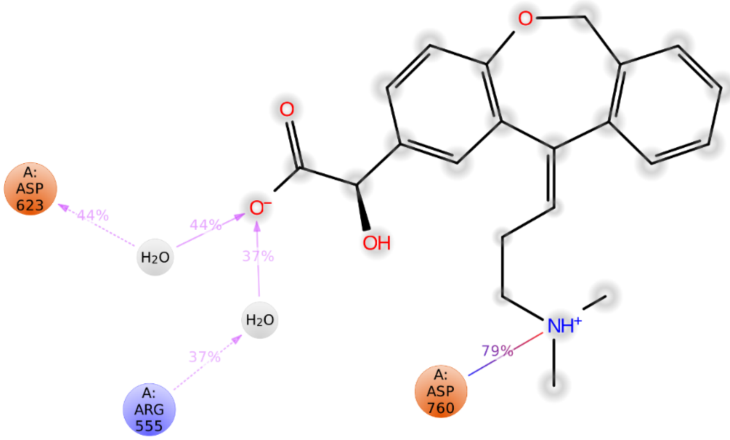

ZINC000067790716	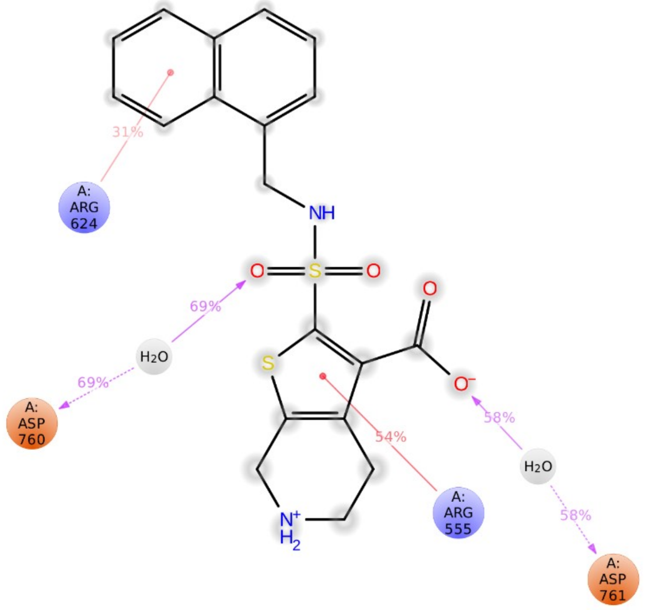
ZINC000069492350_2	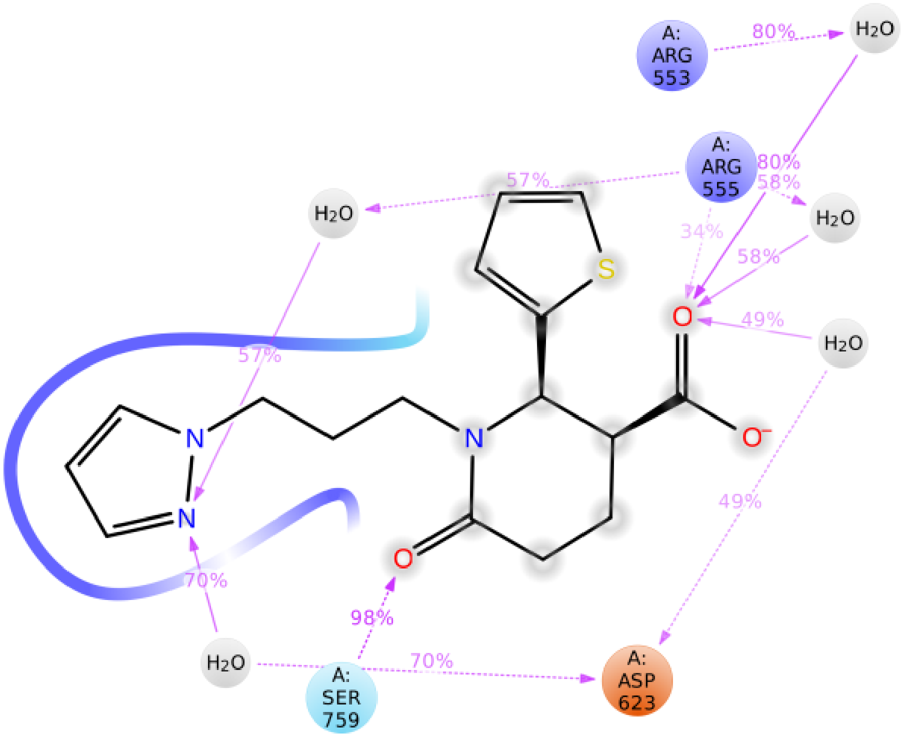

ZINC000084651559	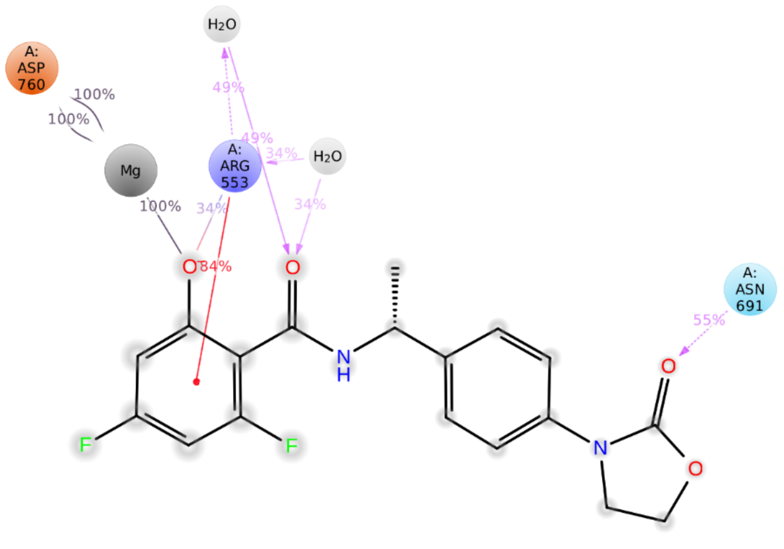
ZINC000089920955	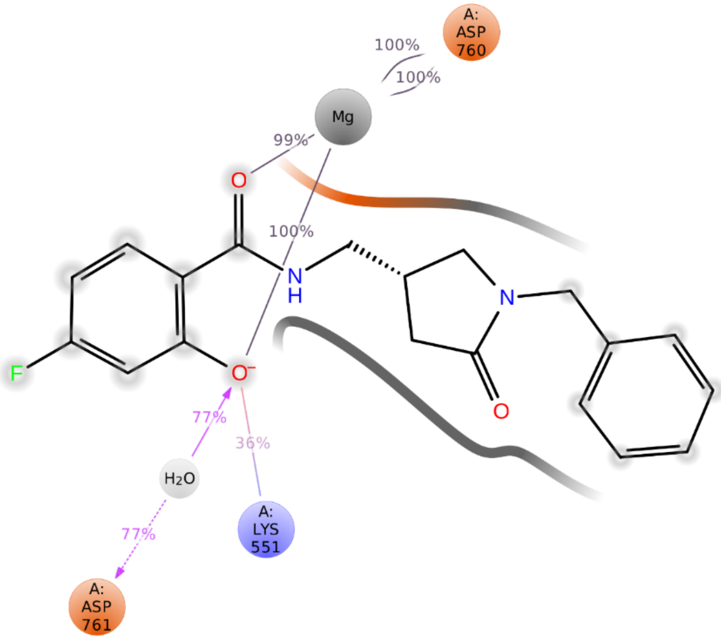

ZINC000097971592_2

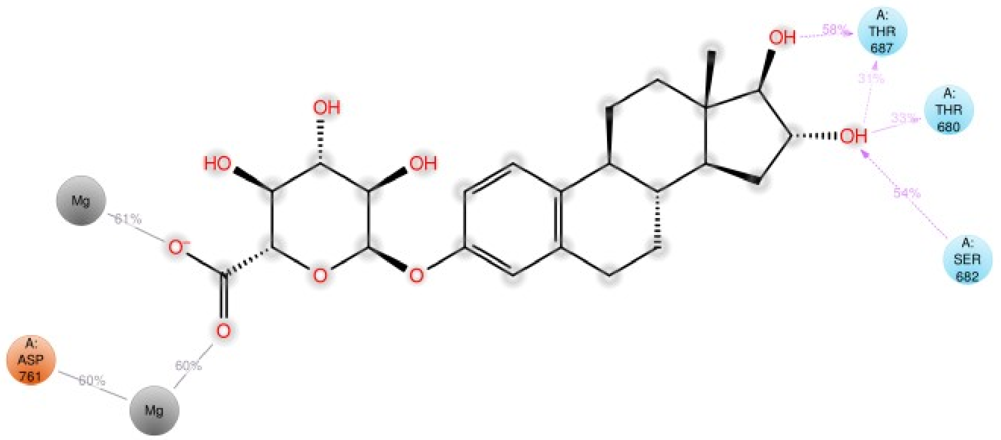

ZINC000237948681

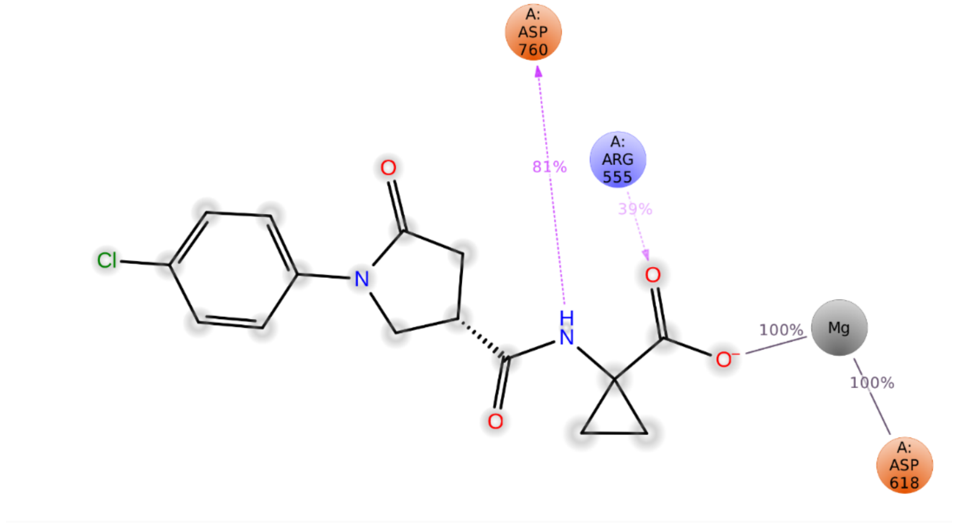

ZINC000238950253	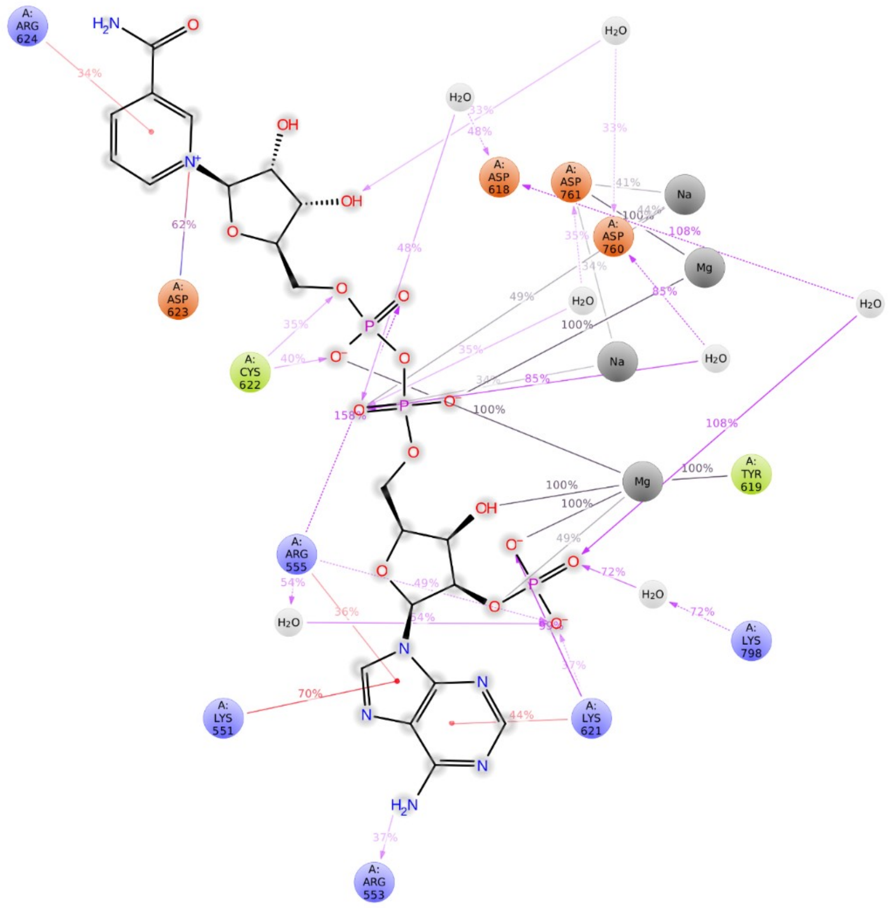
ZINC000257306096	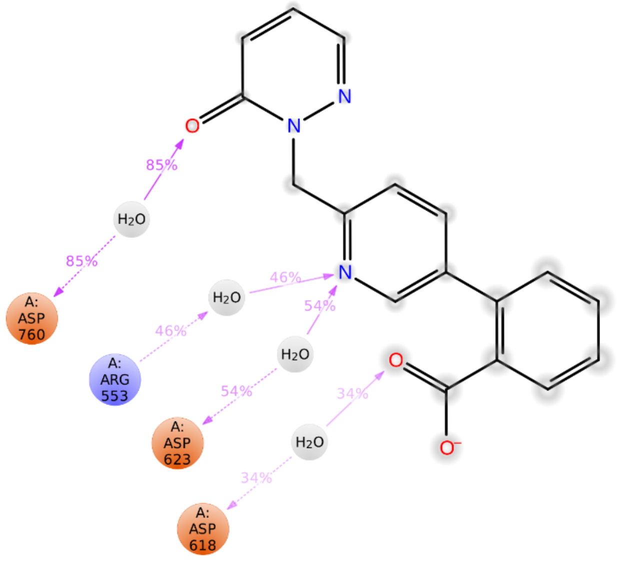

ZINC000299798705	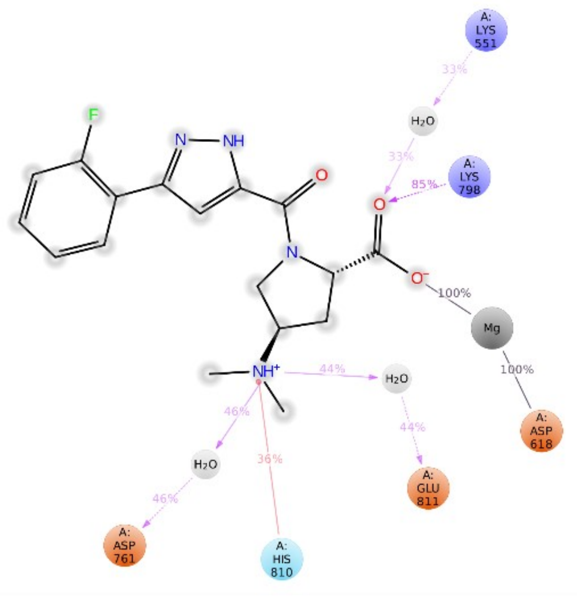
ZINC000408592119_2	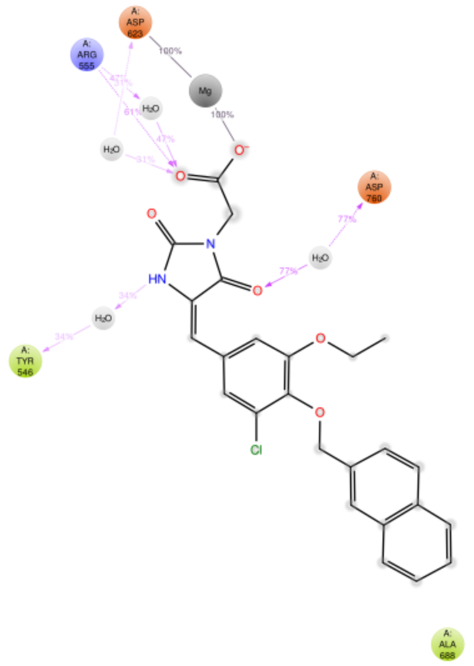

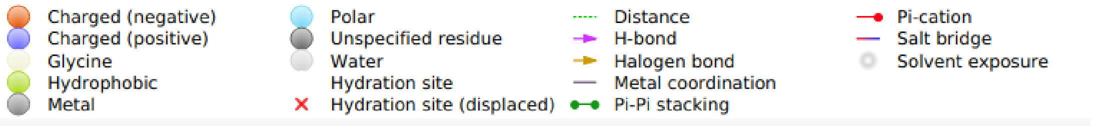

Table S8. Secondary structure elements (SSE) of RdRp protein of each ZINC containing system.

ZINC000002146610<br>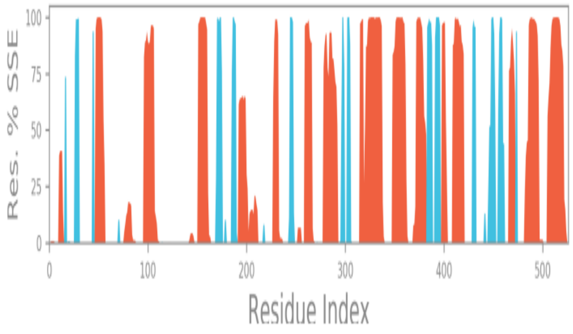	ZINC000014651456<br>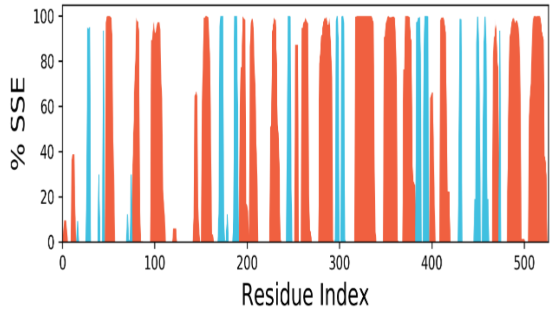
ZINC000016040970<br>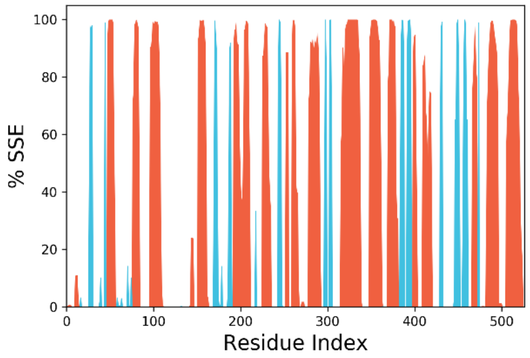	ZINC000065742965<br>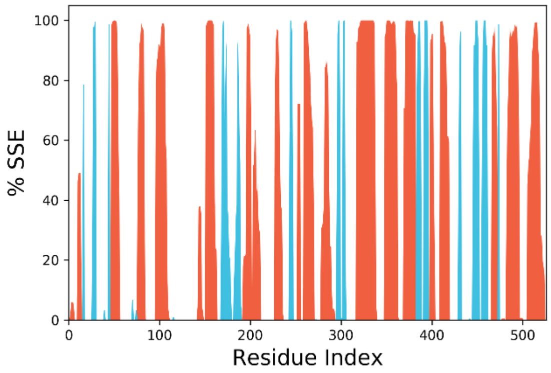
ZINC000067790716<br>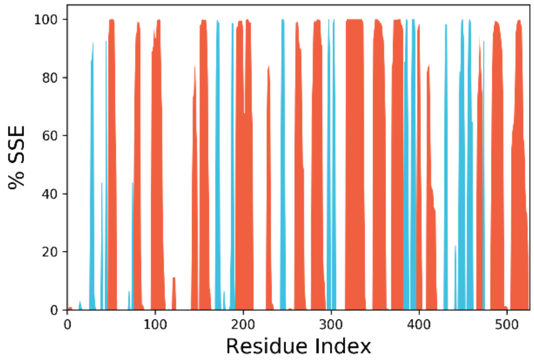	ZINC000069492350<br>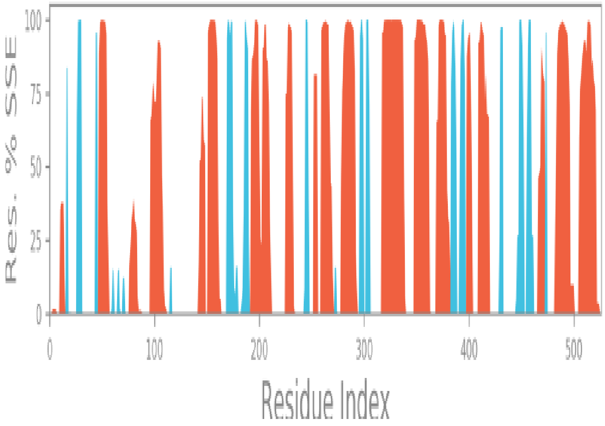
ZINC000084651559	ZINC000089920955

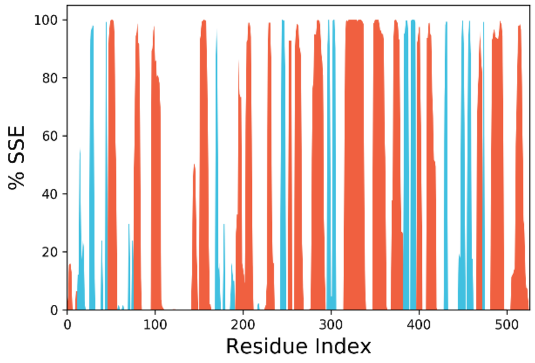

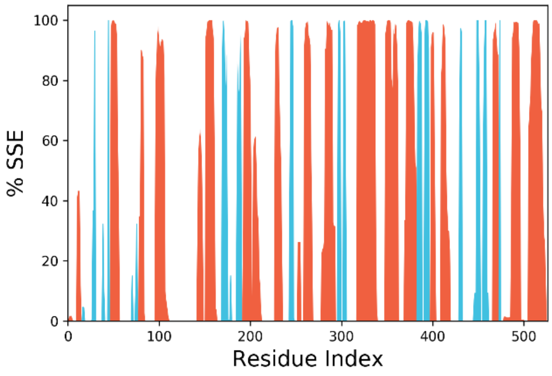

ZINC000097971592

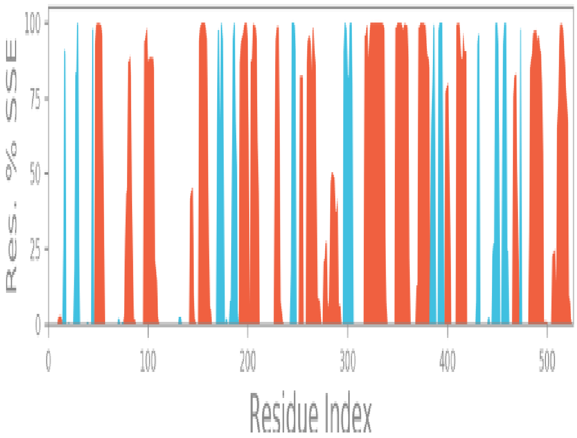

ZINC000237948681

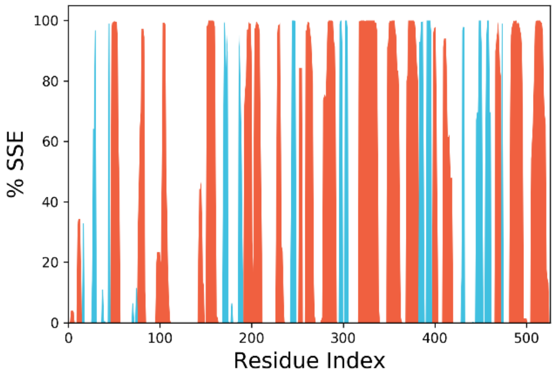

ZINC000238950253

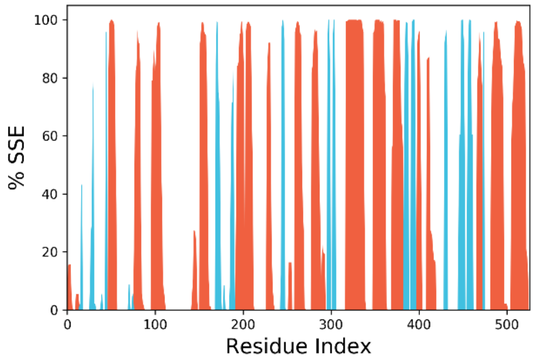

ZINC000257306096

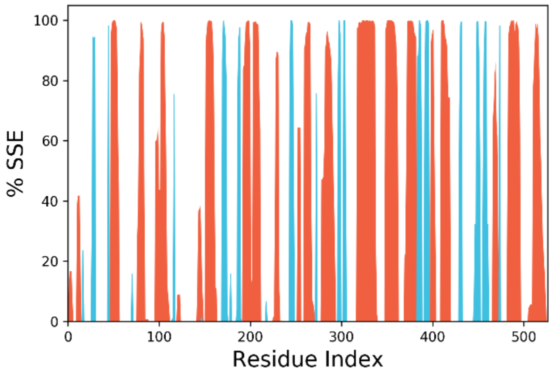

ZINC000299798705

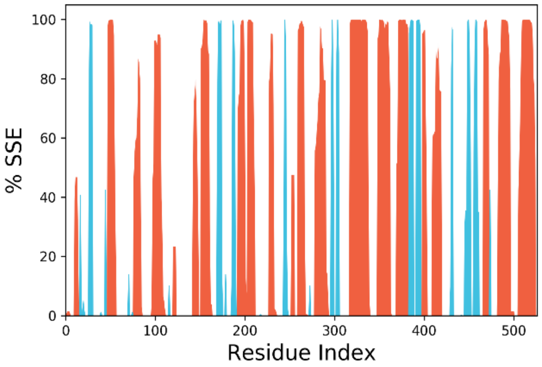

ZINC000408592119

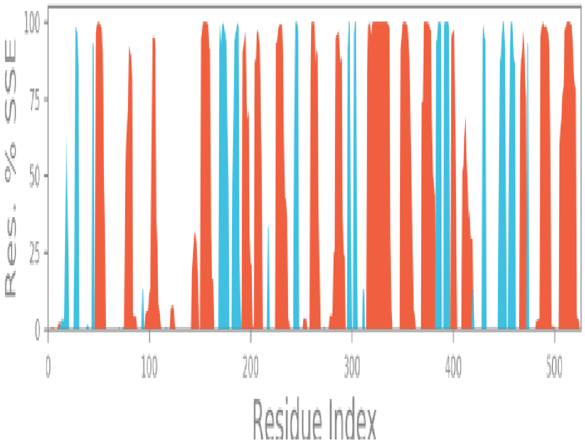

Table S9. Most abundant conformation of each ZINC complex from simulation with zoomed perspective of the most abundant ligand pose with RNA and magnesium ions (left). Detailed 2D protein-ligand contacts diagram of most abundant conformational pose (right).

ZINC000014651456	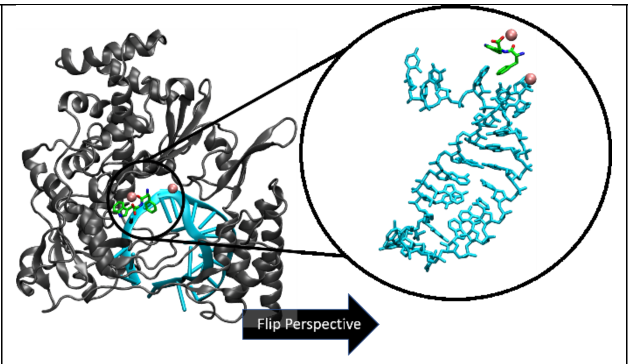	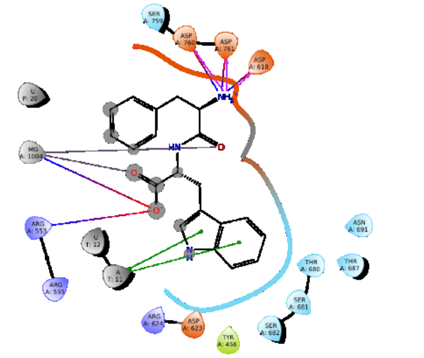
ZINC000016040970	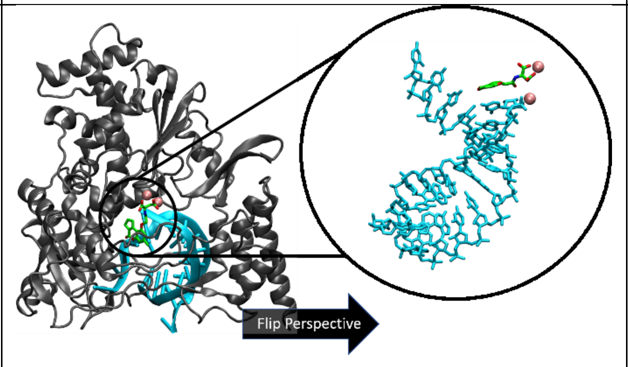	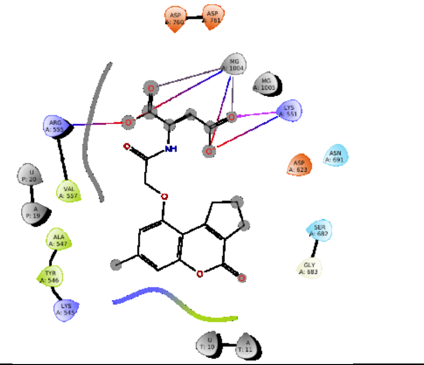
ZINC000065742965	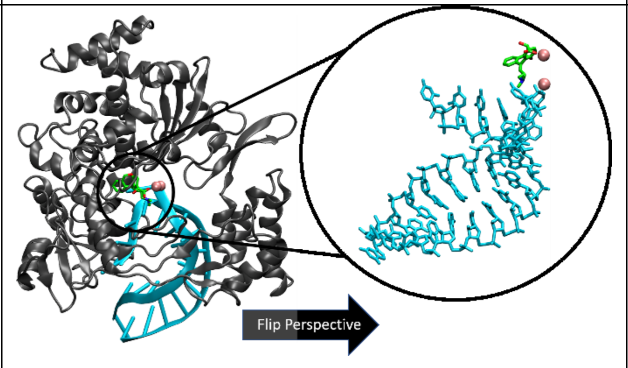	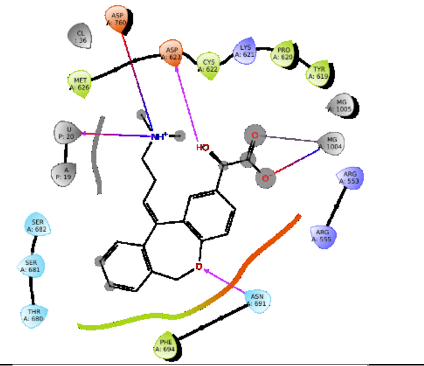
ZINC000067790716	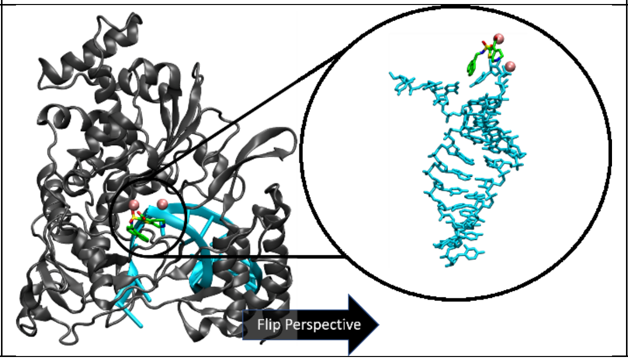	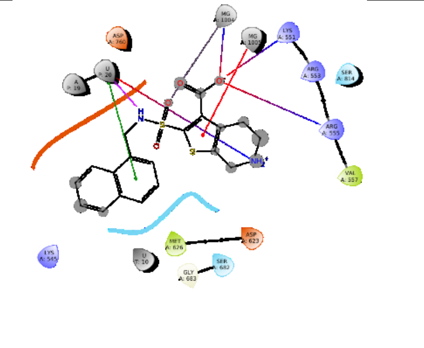

ZINC000238950253	ZINC000237948681	ZINC000089920955	ZINC000084651559
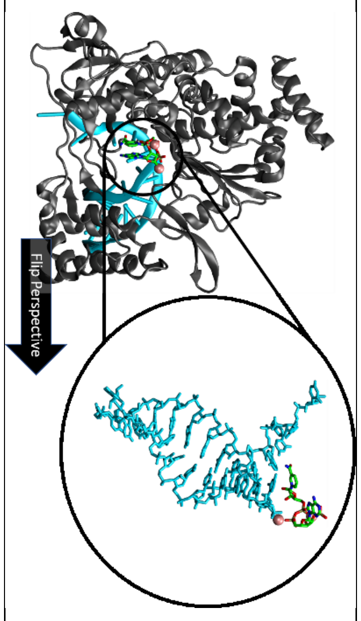	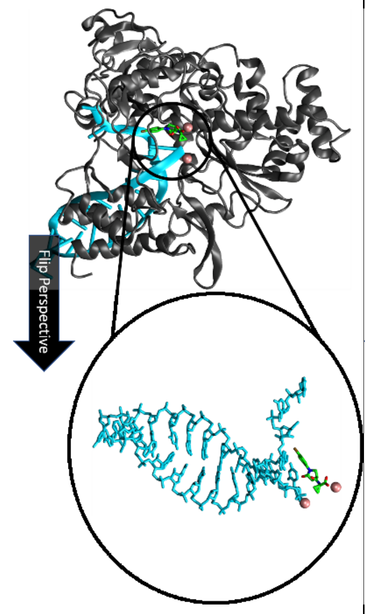	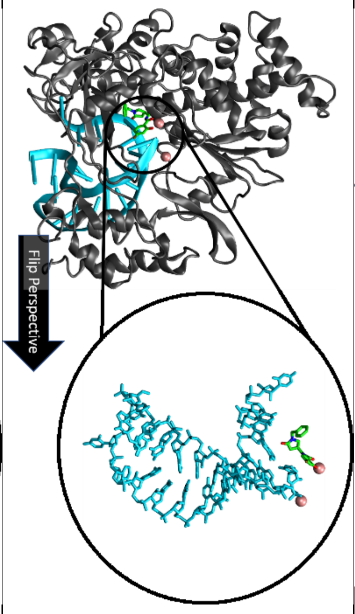	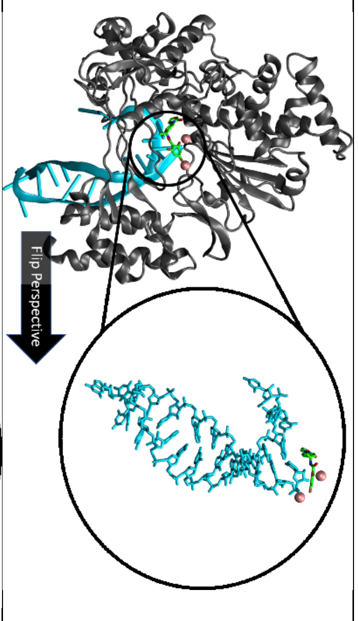
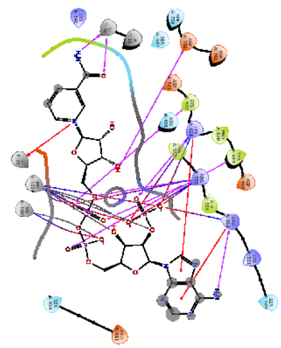	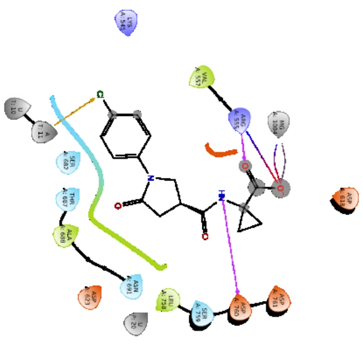	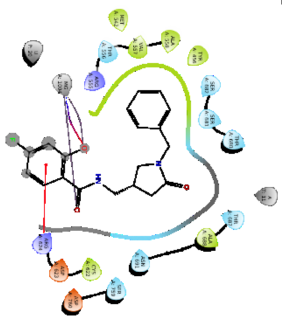	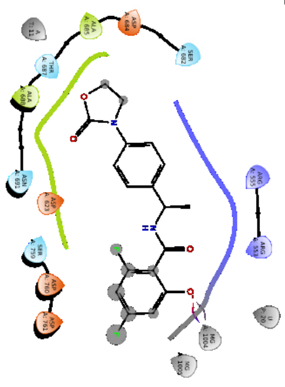

ZINC000257306096	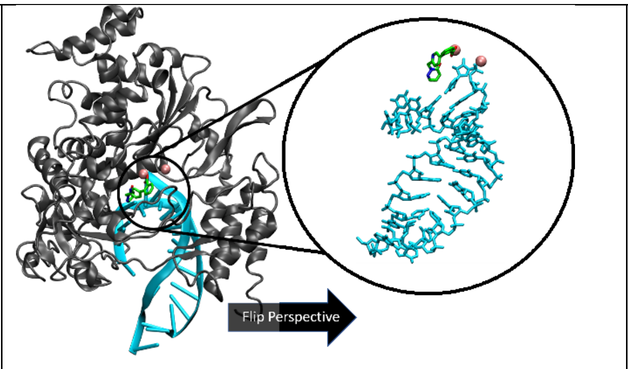	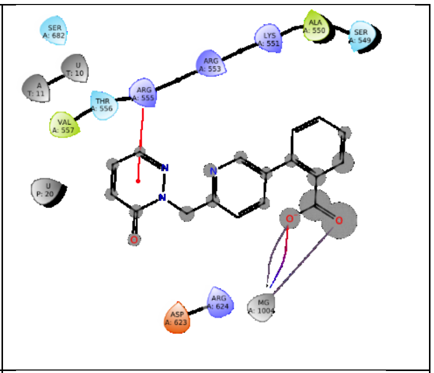
ZINC000299798705	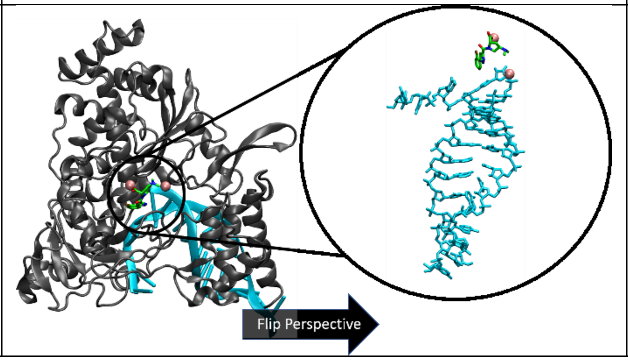	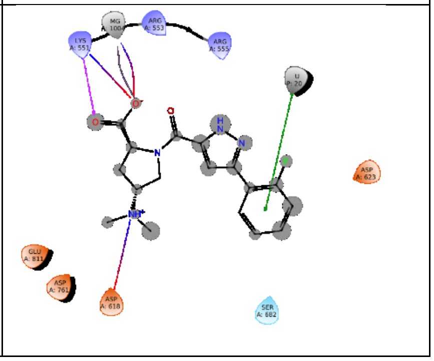

Table S10. Most abundant conformational pose of RdRp in complex with ZINC compounds.

ZINC000002146610 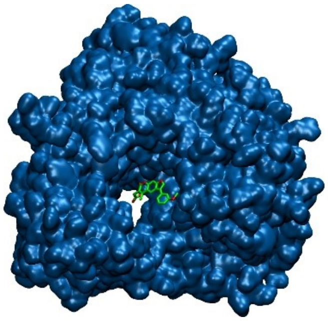	ZINC000014651456 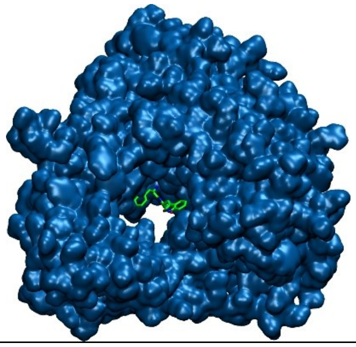
ZINC000016040970 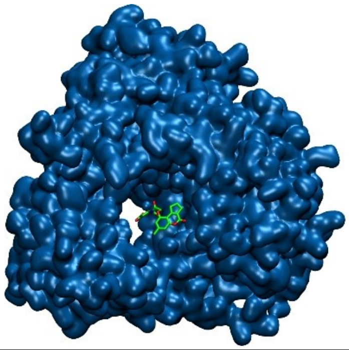	ZINC000065742965 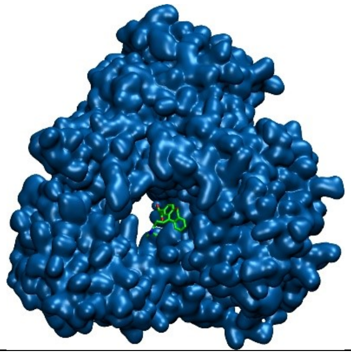
ZINC000067790716 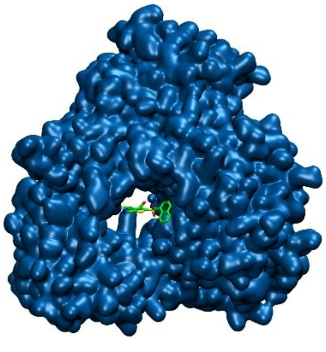	ZINC000069492350 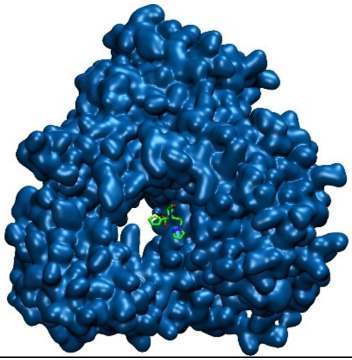
ZINC000084651559 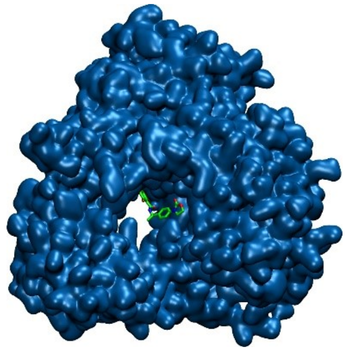	ZINC000089920955 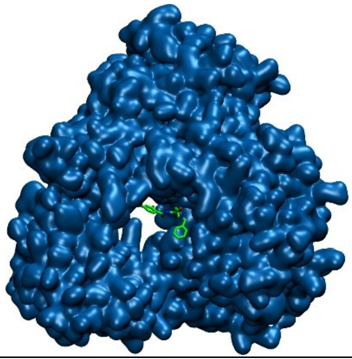
ZINC000097971592	ZINC000237948681

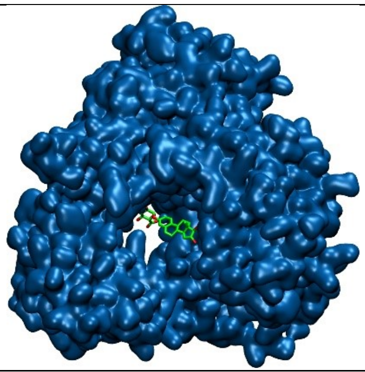	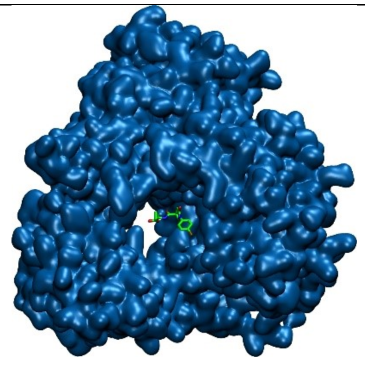
ZINC000238950253	ZINC000257306096
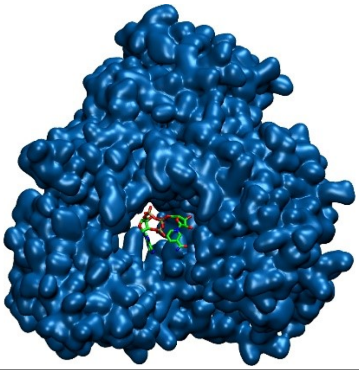	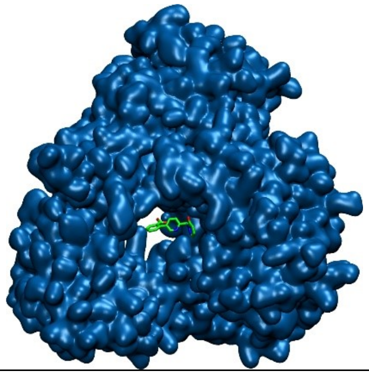
ZINC000299798705	ZINC000408592119
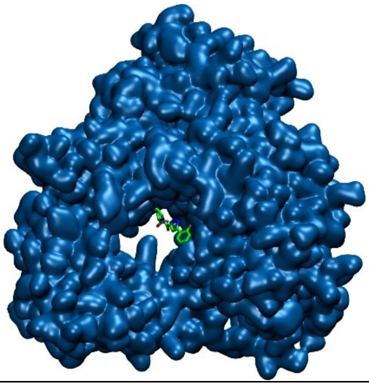	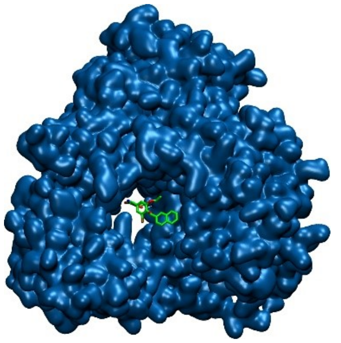

Table S11. Total Protein-Ligand Contacts heatmap summarizing H-bonds, hydrophobic, ionic and water bridge interactions with corresponding individual protein residues.

ATP

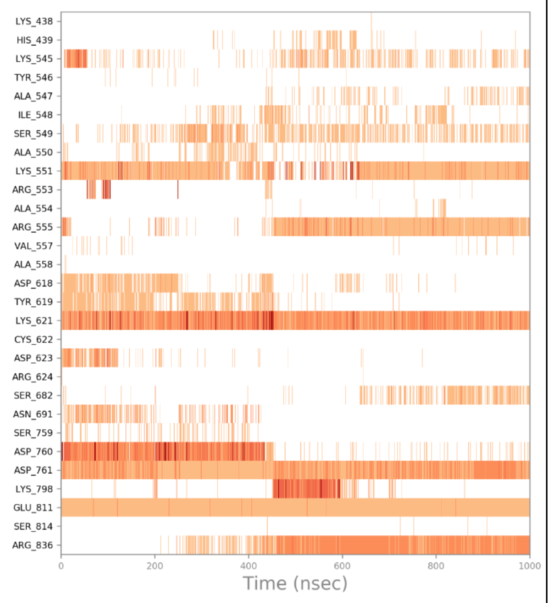

RTP

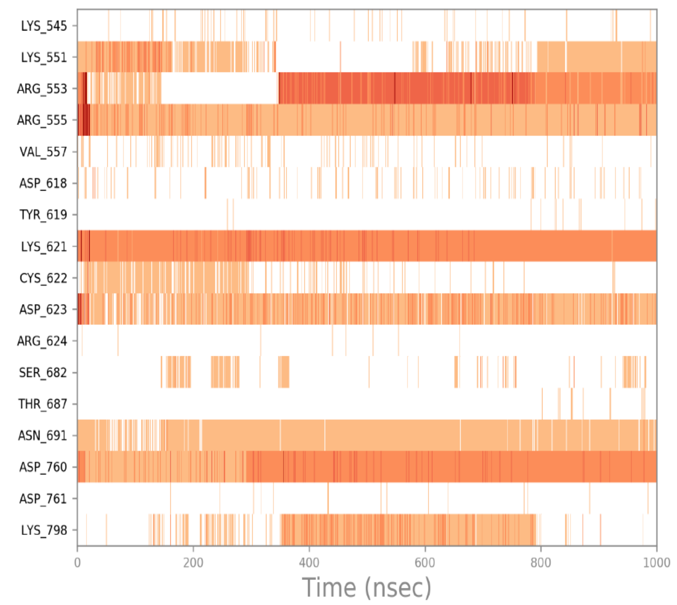

ZINC097971592

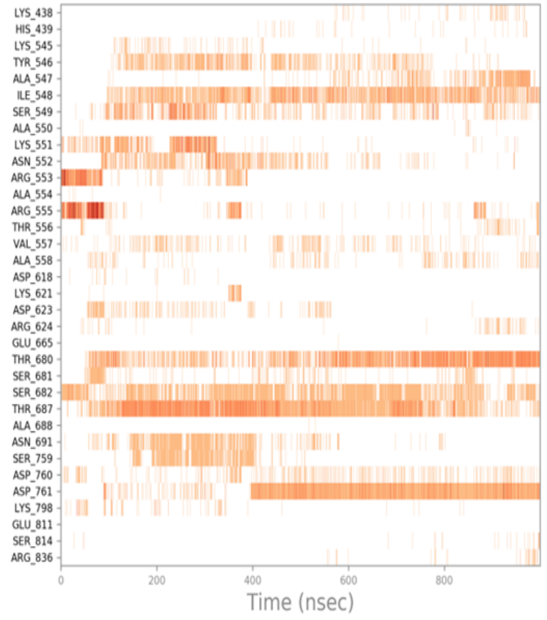

ZINC002146610

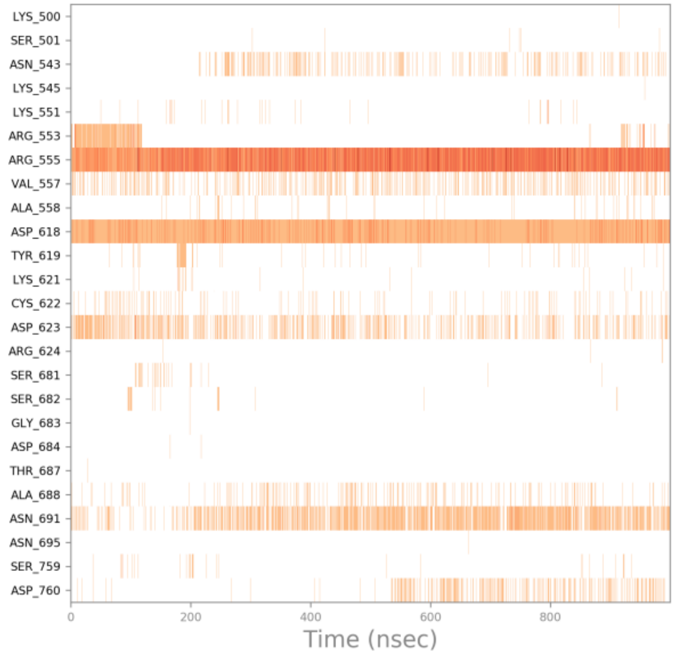

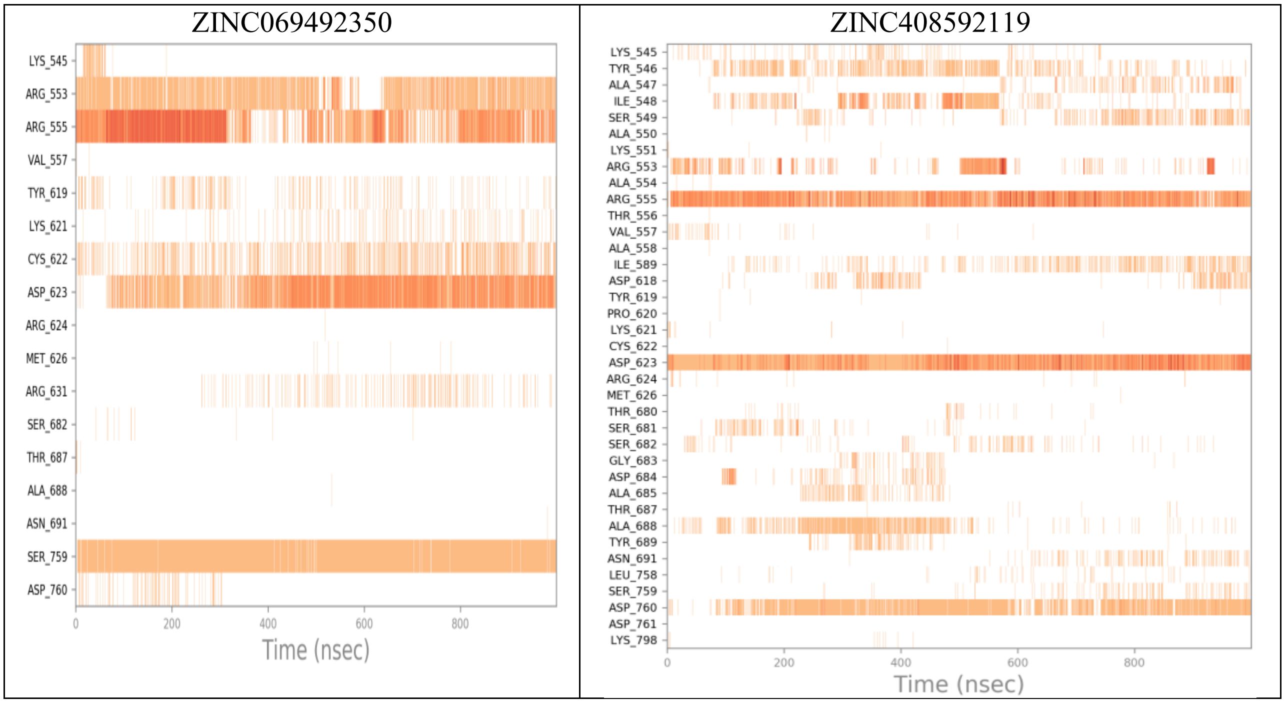
ZINC069492350
LYS_545
ARG_553
ARG_555
VAL_557
TYR_619
LYS_621
CYS_622
ASP_623
ARG_624
MET_626
ARG_631
SER_682
THR_687
ALA_688
ASN_691
SER_759
ASP_760
0
200
400
600
800
Time (nsec)
ZINC408592119
LYS_545
TYR_546
ALA_547
ILE_548
SER_549
ALA_550
LYS_551
ARG_553
ALA_554
ARG_555
THR_556
VAL_557
ALA_558
ILE_589
ASP_618
TYR_619
PRO_620
LYS_621
CYS_622
ASP_623
ARG_624
MET_626
THR_680
SER_681
SER_682
GLY_683
ASP_684
ALA_685
THR_687
ALA_688
TYR_689
ASN_691
LEU_758
SER_759
ASP_760
ASP_761
LYS_798
0
200
400
600
800
Time (nsec)

Table S12. The ADME properties of prodrug remdesivir predicted by SwissADME webserver.

Molecule 1

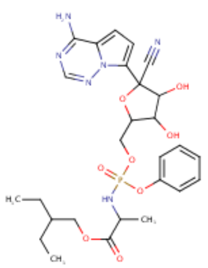

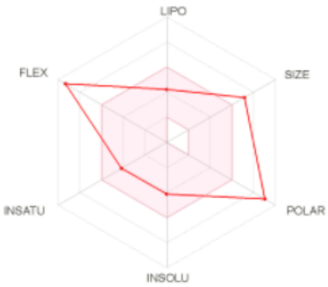

SMILES CCC(COC(=O)C(NP(=O)(Oc1ccccc1)OCC1OC(C(C1O)O)(C#N)c1ccc2n1ncnc2N)C)CC

Physicochemical Properties	
Formula	C27H35N6O8P
Molecular weight	602.58 g/mol
Num. heavy atoms	42
Num. arom. heavy atoms	15
Fraction Csp3	0.48
Num. rotatable bonds	14
Num. H-bond acceptors	12
Num. H-bond donors	4
Molar Refractivity	150.43
TPSA	213.36 Å²

Lipophilicity	
Log $P_{o/w}$ (iLOGP)	3.24
Log $P_{o/w}$ (XLOGP3)	1.91
Log $P_{o/w}$ (WLOGP)	2.21
Log $P_{o/w}$ (MLOGP)	0.18
Log $P_{o/w}$ (SILICOS-IT)	-0.05
Consensus Log $P_{o/w}$	1.50

Water Solubility	
Log *S* (ESOL)	-4.12
Solubility	4.58e-02 mg/ml ; 7.59e-05 mol/l
Class	Moderately soluble
Log *S* (Ali)	-6.01
Solubility	5.84e-04 mg/ml ; 9.69e-07 mol/l
Class	Poorly soluble
Log *S* (SILICOS-IT)	-4.77
Solubility	1.03e-02 mg/ml ; 1.71e-05 mol/l
Class	Moderately soluble

Pharmacokinetics	
GI absorption	Low
BBB permeant	No
P-gp substrate	Yes
CYP1A2 inhibitor	No
CYP2C19 inhibitor	No
CYP2C9 inhibitor	No
CYP2D6 inhibitor	No
CYP3A4 inhibitor	Yes
Log K_p (skin permeation)	-8.62 cm/s

Druglikeness	
Lipinski	No; 2 violations: MW>500, NorO>10
Ghose	No; 3 violations: MW>480, MR>130, #atoms>70
Veber	No; 2 violations: Rotors>10, TPSA>140
Egan	No; 1 violation: TPSA>131.6
Muegge	No; 3 violations: MW>600, TPSA>150, H-acc>10
Bioavailability Score	0.17

Medicinal Chemistry	
PAINS	0 alert
Brenk	1 alert: phosphor
Leadlikeness	No; 2 violations: MW>350, Rotors>7
Synthetic accessibility	6.33

Table S13. The ADME properties of ATP predicted by SwissADME webserver.

Molecule 3

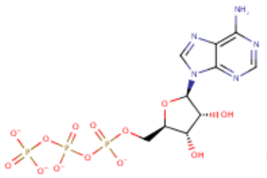

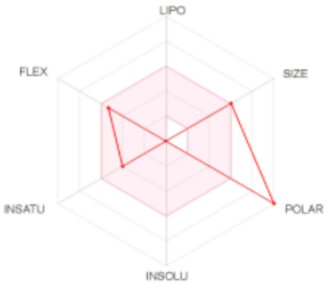

SMILES O[C@@H]1[C@@H](COP(=O)(OP(=O)(OP(=O)([O-])[O-])[O-])[O-])O[C@H]([C@@H]1O)n1cnc2c1ncnc2N

Physicochemical Properties	
Formula	C10H12N5O13P3
Molecular weight	503.15 g/mol
Num. heavy atoms	31
Num. arom. heavy atoms	9
Fraction Csp3	0.50
Num. rotatable bonds	8
Num. H-bond acceptors	16
Num. H-bond donors	3
Molar Refractivity	89.28
TPSA	319.88 Å²

Lipophilicity	
Log $P_{o/w}$ (iLOGP)	-1.17
Log $P_{o/w}$ (XLOGP3)	-5.71
Log $P_{o/w}$ (WLOGP)	-0.19
Log $P_{o/w}$ (MLOGP)	-4.38
Log $P_{o/w}$ (SILICOS-IT)	-5.68
Consensus Log $P_{o/w}$	-3.43

Water Solubility	
Log *S* (ESOL)	0.95
Solubility	4.49e+03 mg/ml ; 8.93e+00 mol/l
Class	Highly soluble
Log *S* (Ali)	-0.34
Solubility	2.28e+02 mg/ml ; 4.54e-01 mol/l
Class	Very soluble
Log *S* (SILICOS-IT)	3.05
Solubility	5.59e+05 mg/ml ; 1.11e+03 mol/l
Class	Soluble

Pharmacokinetics	
GI absorption	Low
BBB permeant	No
P-gp substrate	Yes
CYP1A2 inhibitor	No
CYP2C19 inhibitor	No
CYP2C9 inhibitor	No
CYP2D6 inhibitor	No
CYP3A4 inhibitor	No
Log K_p (skin permeation)	-13.42 cm/s

Druglikeness	
Lipinski	No; 2 violations: MW>500, NorO>10
Ghose	No; 1 violation: MW>480
Veber	No; 1 violation: TPSA>140
Egan	No; 1 violation: TPSA>131.6
Muegge	No; 3 violations: XLOGP3<-2, TPSA>150, H-acc>10
Bioavailability Score	0.11

Medicinal Chemistry	
PAINS	0 alert
Brenk	1 alert: phosphor
Leadlikeness	No; 2 violations: MW>350, Rotors>7
Synthetic accessibility	4.74

Table S14. The ADME properties of RTP predicted by SwissADME webserver.

Molecule 2

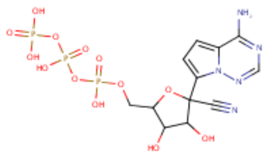

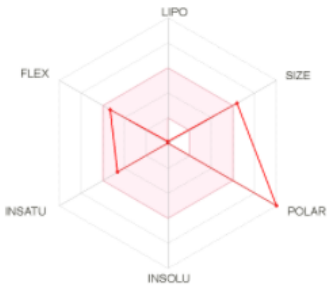

SMILES N#CC1(OC(C(C1O)O)COP(=O)(OP(=O)(OP(=O)(O)O)O)O)c1ccc2n1ncnc2N

Physicochemical Properties	
Formula	C12H16N5O13P3
Molecular weight	531.20 g/mol
Num. heavy atoms	33
Num. arom. heavy atoms	9
Fraction Csp3	0.42
Num. rotatable bonds	8
Num. H-bond acceptors	16
Num. H-bond donors	7
Molar Refractivity	101.21
TPSA	318.94 Å²

Lipophilicity	
Log $P_{o/w}$ (iLOGP)	-0.99
Log $P_{o/w}$ (XLOGP3)	-5.25
Log $P_{o/w}$ (WLOGP)	-1.61
Log $P_{o/w}$ (MLOGP)	-4.29
Log $P_{o/w}$ (SILICOS-IT)	-5.27
Consensus Log $P_{o/w}$	-3.48

Water Solubility	
Log *S* (ESOL)	0.50
Solubility	1.68e+03 mg/ml ; 3.16e+00 mol/l
Class	Highly soluble
Log *S* (Ali)	-0.80
Solubility	8.40e+01 mg/ml ; 1.58e-01 mol/l
Class	Very soluble
Log *S* (SILICOS-IT)	2.40
Solubility	1.33e+05 mg/ml ; 2.49e+02 mol/l
Class	Soluble

Pharmacokinetics	
GI absorption	Low
BBB permeant	No
P-gp substrate	No
CYP1A2 inhibitor	No
CYP2C19 inhibitor	No
CYP2C9 inhibitor	No
CYP2D6 inhibitor	No
CYP3A4 inhibitor	No
Log K_p (skin permeation)	-13.27 cm/s

Druglikeness	
Lipinski	No; 3 violations: MW>500, NorO>10, NHorOH>5
Ghose	No; 2 violations: MW>480, WLOGP<-0.4
Veber	No; 1 violation: TPSA>140
Egan	No; 1 violation: TPSA>131.6
Muegge	No; 4 violations: XLOGP3<-2, TPSA>150, H-acc>10, H-don>5
Bioavailability Score	0.11

Medicinal Chemistry	
PAINS	0 alert
Brenk	1 alert: phosphor
Leadlikeness	No; 2 violations: MW>350, Rotors>7
Synthetic accessibility	4.82

Table S15. The ADME properties of ZINC000002146610 predicted by SwissADME webserver.

Molecule 4

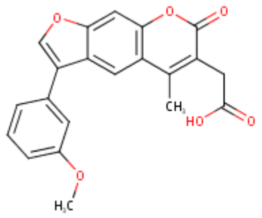

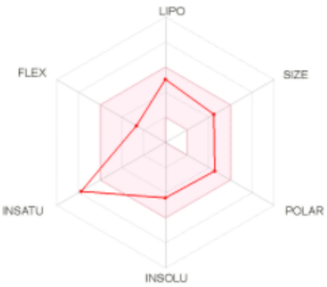

SMILES COc1cccc(c1)c1coc2c1cc1c(c2)oc(=O)c(c1C)CC(=O)O

Physicochemical Properties	
Formula	C21H16O6
Molecular weight	364.35 g/mol
Num. heavy atoms	27
Num. arom. heavy atoms	19
Fraction Csp3	0.14
Num. rotatable bonds	4
Num. H-bond acceptors	6
Num. H-bond donors	1
Molar Refractivity	100.69
TPSA	89.88 Å²

Lipophilicity	
Log $P_{o/w}$ (iLOGP)	2.73
Log $P_{o/w}$ (XLOGP3)	3.30
Log $P_{o/w}$ (WLOGP)	4.15
Log $P_{o/w}$ (MLOGP)	2.26
Log $P_{o/w}$ (SILICOS-IT)	4.75
Consensus Log $P_{o/w}$	3.44

Water Solubility	
Log *S* (ESOL)	-4.43
Solubility	1.34e-02 mg/ml ; 3.68e-05 mol/l
Class	Moderately soluble
Log *S* (Ali)	-4.86
Solubility	4.99e-03 mg/ml ; 1.37e-05 mol/l
Class	Moderately soluble
Log *S* (SILICOS-IT)	-7.26
Solubility	1.99e-05 mg/ml ; 5.47e-08 mol/l
Class	Poorly soluble

Pharmacokinetics	
GI absorption	High
BBB permeant	No
P-gp substrate	No
CYP1A2 inhibitor	Yes
CYP2C19 inhibitor	No
CYP2C9 inhibitor	Yes
CYP2D6 inhibitor	No
CYP3A4 inhibitor	No
Log K_p (skin permeation)	-6.18 cm/s

Druglikeness	
Lipinski	Yes; 0 violation
Ghose	Yes
Veber	Yes
Egan	Yes
Muegge	Yes
Bioavailability Score	0.56

Medicinal Chemistry	
PAINS	0 alert
Brenk	1 alert: cumarine
Leadlikeness	No; 1 violation: MW>350
Synthetic accessibility	3.54

Table S16. The ADME properties of ZINC000014651456 predicted by SwissADME webserver.

Molecule 6

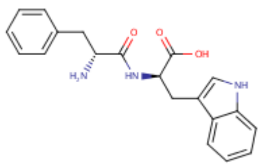

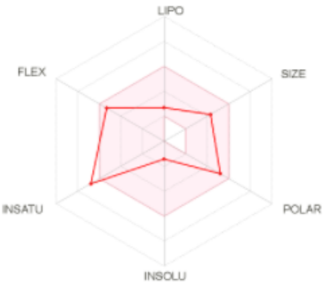

SMILES N[C@@H](C(=O)N[C@@H](C(=O)O)Cc1c[nH]c2c1cccc2)Cc1ccccc1

Physicochemical Properties	
Formula	C20H21N3O3
Molecular weight	351.40 g/mol
Num. heavy atoms	26
Num. arom. heavy atoms	15
Fraction Csp3	0.20
Num. rotatable bonds	8
Num. H-bond acceptors	4
Num. H-bond donors	4
Molar Refractivity	99.27
TPSA	108.21 Å²

Lipophilicity	
Log $P_{o/w}$ (iLOGP)	1.72
Log $P_{o/w}$ (XLOGP3)	-0.79
Log $P_{o/w}$ (WLOGP)	1.85
Log $P_{o/w}$ (MLOGP)	1.27
Log $P_{o/w}$ (SILICOS-IT)	2.80
Consensus Log $P_{o/w}$	1.37

Water Solubility	
Log *S* (ESOL)	-1.42
Solubility	1.34e+01 mg/ml ; 3.80e-02 mol/l
Class	Very soluble
Log *S* (Ali)	-1.00
Solubility	3.48e+01 mg/ml ; 9.91e-02 mol/l
Class	Very soluble
Log *S* (SILICOS-IT)	-5.68
Solubility	7.31e-04 mg/ml ; 2.08e-06 mol/l
Class	Moderately soluble

Pharmacokinetics	
GI absorption	High
BBB permeant	No
P-gp substrate	Yes
CYP1A2 inhibitor	No
CYP2C19 inhibitor	No
CYP2C9 inhibitor	No
CYP2D6 inhibitor	Yes
CYP3A4 inhibitor	No
Log K_p (skin permeation)	-9.00 cm/s

Druglikeness	
Lipinski	Yes; 0 violation
Ghose	Yes
Veber	Yes
Egan	Yes
Muegge	Yes
Bioavailability Score	0.55

Medicinal Chemistry	
PAINS	0 alert
Brenk	0 alert
Leadlikeness	No; 2 violations: MW>350, Rotors>7
Synthetic accessibility	3.16

Table S17. The ADME properties of ZINC000016040970 predicted by SwissADME webserver.

Molecule 7

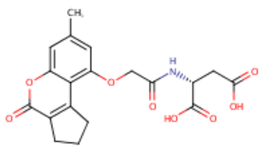

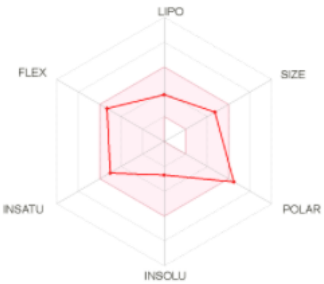

SMILES O=C(N[C@@H](C(=O)O)CC(=O)O)COc1cc(C)cc2c1c1CCCc1c(=O)o2

Physicochemical Properties	
Formula	C19H19NO8
Molecular weight	389.36 g/mol
Num. heavy atoms	28
Num. arom. heavy atoms	10
Fraction Csp3	0.37
Num. rotatable bonds	8
Num. H-bond acceptors	8
Num. H-bond donors	3
Molar Refractivity	97.15
TPSA	143.14 Å²

Lipophilicity	
Log $P_{o/w}$ (iLOGP)	1.73
Log $P_{o/w}$ (XLOGP3)	1.11
Log $P_{o/w}$ (WLOGP)	1.01
Log $P_{o/w}$ (MLOGP)	0.61
Log $P_{o/w}$ (SILICOS-IT)	2.47
Consensus Log $P_{o/w}$	1.39

Water Solubility	
Log *S* (ESOL)	-2.69
Solubility	7.96e-01 mg/ml ; 2.04e-03 mol/l
Class	Soluble
Log *S* (Ali)	-3.71
Solubility	7.61e-02 mg/ml ; 1.95e-04 mol/l
Class	Soluble
Log *S* (SILICOS-IT)	-4.11
Solubility	3.05e-02 mg/ml ; 7.83e-05 mol/l
Class	Moderately soluble

Pharmacokinetics	
GI absorption	Low
BBB permeant	No
P-gp substrate	Yes
CYP1A2 inhibitor	No
CYP2C19 inhibitor	No
CYP2C9 inhibitor	No
CYP2D6 inhibitor	No
CYP3A4 inhibitor	No
Log K_p (skin permeation)	-7.89 cm/s

Druglikeness	
Lipinski	Yes; 0 violation
Ghose	Yes
Veber	No; 1 violation: TPSA>140
Egan	No; 1 violation: TPSA>131.6
Muegge	Yes
Bioavailability Score	0.56

Medicinal Chemistry	
PAINS	0 alert
Brenk	1 alert: cumarine
Leadlikeness	No; 2 violations: MW>350, Rotors>7
Synthetic accessibility	3.96

Table S18. The ADME properties of ZINC000065742965 predicted by SwissADME webserver.

Molecule 8

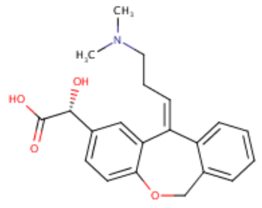

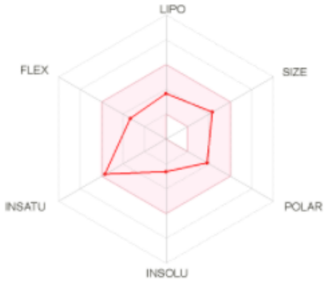

SMILES CN(CC/C=C/1\c2cc(ccc2OCc2c1cccc2)[C@H](C(=O)O)O)C

Physicochemical Properties	
Formula	C21H23NO4
Molecular weight	353.41 g/mol
Num. heavy atoms	26
Num. arom. heavy atoms	12
Fraction Csp3	0.29
Num. rotatable bonds	5
Num. H-bond acceptors	5
Num. H-bond donors	2
Molar Refractivity	100.39
TPSA	70.00 Å²

Lipophilicity	
Log $P_{o/w}$ (iLOGP)	2.72
Log $P_{o/w}$ (XLOGP3)	0.92
Log $P_{o/w}$ (WLOGP)	2.60
Log $P_{o/w}$ (MLOGP)	2.05
Log $P_{o/w}$ (SILICOS-IT)	3.28
Consensus Log $P_{o/w}$	2.32

Water Solubility	
Log *S* (ESOL)	-2.62
Solubility	8.43e-01 mg/ml ; 2.39e-03 mol/l
Class	Soluble
Log *S* (Ali)	-1.98
Solubility	3.74e+00 mg/ml ; 1.06e-02 mol/l
Class	Very soluble
Log *S* (SILICOS-IT)	-4.96
Solubility	3.86e-03 mg/ml ; 1.09e-05 mol/l
Class	Moderately soluble

Pharmacokinetics	
GI absorption	High
BBB permeant	Yes
P-gp substrate	Yes
CYP1A2 inhibitor	No
CYP2C19 inhibitor	No
CYP2C9 inhibitor	No
CYP2D6 inhibitor	Yes
CYP3A4 inhibitor	No
Log K_p (skin permeation)	-7.80 cm/s

Druglikeness	
Lipinski	Yes; 0 violation
Ghose	Yes
Veber	Yes
Egan	Yes
Muegge	Yes
Bioavailability Score	0.55

Medicinal Chemistry	
PAINS	0 alert
Brenk	0 alert
Leadlikeness	No; 1 violation: MW>350
Synthetic accessibility	4.38

Table S19. The ADME properties of ZINC000067790716 predicted by SwissADME webserver.

Molecule 9

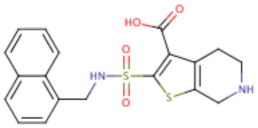

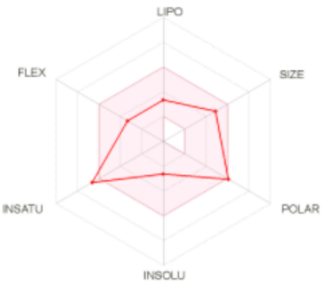

SMILES OC(=O)c1c2CCNCc2sc1S(=O)(=O)NCc1cccc2c1cccc2

Physicochemical Properties	
Formula	C19H18N2O4S2
Molecular weight	402.49 g/mol
Num. heavy atoms	27
Num. arom. heavy atoms	15
Fraction Csp3	0.21
Num. rotatable bonds	5
Num. H-bond acceptors	6
Num. H-bond donors	3
Molar Refractivity	108.51
TPSA	132.12 Å²

Lipophilicity	
Log $P_{o/w}$ (iLOGP)	2.48
Log $P_{o/w}$ (XLOGP3)	0.33
Log $P_{o/w}$ (WLOGP)	3.12
Log $P_{o/w}$ (MLOGP)	1.65
Log $P_{o/w}$ (SILICOS-IT)	3.41
Consensus Log $P_{o/w}$	2.20

Water Solubility	
Log *S* (ESOL)	-2.62
Solubility	9.56e-01 mg/ml ; 2.37e-03 mol/l
Class	Soluble
Log *S* (Ali)	-2.67
Solubility	8.64e-01 mg/ml ; 2.15e-03 mol/l
Class	Soluble
Log *S* (SILICOS-IT)	-6.42
Solubility	1.54e-04 mg/ml ; 3.84e-07 mol/l
Class	Poorly soluble

Pharmacokinetics	
GI absorption	High
BBB permeant	No
P-gp substrate	Yes
CYP1A2 inhibitor	Yes
CYP2C19 inhibitor	No
CYP2C9 inhibitor	No
CYP2D6 inhibitor	No
CYP3A4 inhibitor	No
Log K_p (skin permeation)	-8.52 cm/s

Druglikeness	
Lipinski	Yes; 0 violation
Ghose	Yes
Veber	Yes
Egan	No; 1 violation: TPSA>131.6
Muegge	Yes
Bioavailability Score	0.55

Medicinal Chemistry	
PAINS	0 alert
Brenk	0 alert
Leadlikeness	No; 1 violation: MW>350
Synthetic accessibility	3.60

Table S20. The ADME properties of ZINC000069492350 predicted by SwissADME webserver.

Molecule 10

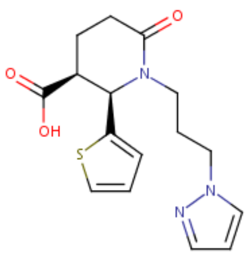

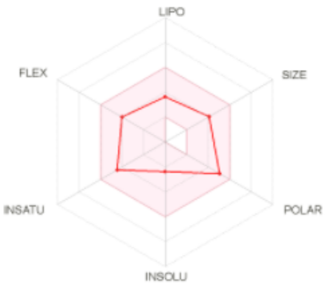

SMILES OC(=O)[C@H]1CCC(=O)N([C@H]1c1cccs1)CCCn1cccn1

Physicochemical Properties	
Formula	C16H19N3O3S
Molecular weight	333.41 g/mol
Num. heavy atoms	23
Num. arom. heavy atoms	10
Fraction Csp3	0.44
Num. rotatable bonds	6
Num. H-bond acceptors	4
Num. H-bond donors	1
Molar Refractivity	90.98
TPSA	103.67 Å²

Lipophilicity	
Log $P_{o/w}$ (iLOGP)	1.97
Log $P_{o/w}$ (XLOGP3)	0.86
Log $P_{o/w}$ (WLOGP)	1.69
Log $P_{o/w}$ (MLOGP)	1.10
Log $P_{o/w}$ (SILICOS-IT)	2.02
Consensus Log $P_{o/w}$	1.53

Water Solubility	
Log *S* (ESOL)	-2.37
Solubility	1.41e+00 mg/ml ; 4.22e-03 mol/l
Class	Soluble
Log *S* (Ali)	-2.62
Solubility	7.99e-01 mg/ml ; 2.40e-03 mol/l
Class	Soluble
Log *S* (SILICOS-IT)	-2.91
Solubility	4.12e-01 mg/ml ; 1.24e-03 mol/l
Class	Soluble

Pharmacokinetics	
GI absorption	High
BBB permeant	No
P-gp substrate	Yes
CYP1A2 inhibitor	No
CYP2C19 inhibitor	No
CYP2C9 inhibitor	No
CYP2D6 inhibitor	No
CYP3A4 inhibitor	No
Log K_p (skin permeation)	-7.72 cm/s

Druglikeness	
Lipinski	Yes; 0 violation
Ghose	Yes
Veber	Yes
Egan	Yes
Muegge	Yes
Bioavailability Score	0.56

Medicinal Chemistry	
PAINS	0 alert
Brenk	0 alert
Leadlikeness	Yes
Synthetic accessibility	3.53

Table S21. The ADME properties of ZINC000084651559 predicted by SwissADME webserver.

Molecule 11

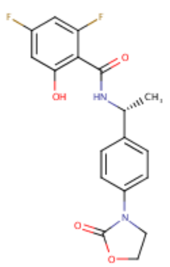

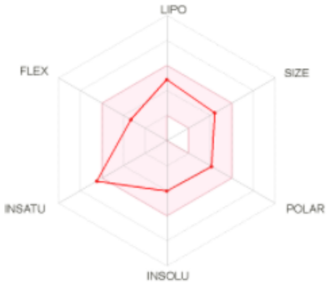

SMILES Fc1cc(O)c(c(c1)F)C(=O)N[C@@H](c1ccc(cc1)N1CCOC1=O)C

Physicochemical Properties	
Formula	C18H16F2N2O4
Molecular weight	362.33 g/mol
Num. heavy atoms	26
Num. arom. heavy atoms	12
Fraction Csp3	0.22
Num. rotatable bonds	5
Num. H-bond acceptors	6
Num. H-bond donors	2
Molar Refractivity	92.18
TPSA	78.87 Å²

Lipophilicity	
Log $P_{o/w}$ (iLOGP)	2.59
Log $P_{o/w}$ (XLOGP3)	3.03
Log $P_{o/w}$ (WLOGP)	3.25
Log $P_{o/w}$ (MLOGP)	2.67
Log $P_{o/w}$ (SILICOS-IT)	2.94
Consensus Log $P_{o/w}$	2.90

Water Solubility	
Log *S* (ESOL)	-4.01
Solubility	3.57e-02 mg/ml ; 9.84e-05 mol/l
Class	Moderately soluble
Log *S* (Ali)	-4.35
Solubility	1.61e-02 mg/ml ; 4.45e-05 mol/l
Class	Moderately soluble
Log *S* (SILICOS-IT)	-5.08
Solubility	3.00e-03 mg/ml ; 8.28e-06 mol/l
Class	Moderately soluble

Pharmacokinetics	
GI absorption	High
BBB permeant	Yes
P-gp substrate	No
CYP1A2 inhibitor	Yes
CYP2C19 inhibitor	No
CYP2C9 inhibitor	Yes
CYP2D6 inhibitor	No
CYP3A4 inhibitor	No
Log K_p (skin permeation)	-6.36 cm/s

Druglikeness	
Lipinski	Yes; 0 violation
Ghose	Yes
Veber	Yes
Egan	Yes
Muegge	Yes
Bioavailability Score	0.55

Medicinal Chemistry	
PAINS	0 alert
Brenk	0 alert
Leadlikeness	No; 1 violation: MW>350
Synthetic accessibility	3.02

Table S22. The ADME properties of ZINC00008992095 predicted by SwissADME webserver.

Molecule 12

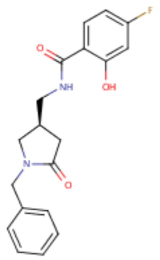

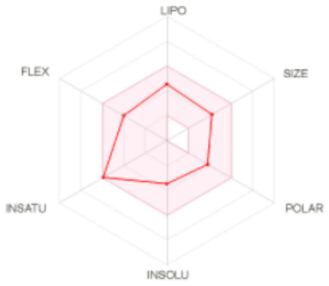

SMILES Fc1ccc(c(c1)O)C(=O)NC[C@H]1CC(=O)N(C1)Cc1ccccc1

Physicochemical Properties	
Formula	C19H19FN2O3
Molecular weight	342.36 g/mol
Num. heavy atoms	25
Num. arom. heavy atoms	12
Fraction Csp3	0.26
Num. rotatable bonds	6
Num. H-bond acceptors	4
Num. H-bond donors	2
Molar Refractivity	94.84
TPSA	69.64 Å²

Lipophilicity	
Log $P_{o/w}$ (iLOGP)	2.72
Log $P_{o/w}$ (XLOGP3)	2.45
Log $P_{o/w}$ (WLOGP)	2.20
Log $P_{o/w}$ (MLOGP)	2.25
Log $P_{o/w}$ (SILICOS-IT)	2.90
Consensus Log $P_{o/w}$	2.51

Water Solubility	
Log *S* (ESOL)	-3.47
Solubility	1.17e-01 mg/ml ; 3.42e-04 mol/l
Class	Soluble
Log *S* (Ali)	-3.56
Solubility	9.52e-02 mg/ml ; 2.78e-04 mol/l
Class	Soluble
Log *S* (SILICOS-IT)	-5.50
Solubility	1.09e-03 mg/ml ; 3.18e-06 mol/l
Class	Moderately soluble

Pharmacokinetics	
GI absorption	High
BBB permeant	Yes
P-gp substrate	Yes
CYP1A2 inhibitor	No
CYP2C19 inhibitor	No
CYP2C9 inhibitor	No
CYP2D6 inhibitor	Yes
CYP3A4 inhibitor	No
Log K_p (skin permeation)	-6.65 cm/s

Druglikeness	
Lipinski	Yes; 0 violation
Ghose	Yes
Veber	Yes
Egan	Yes
Muegge	Yes
Bioavailability Score	0.55

Medicinal Chemistry	
PAINS	0 alert
Brenk	0 alert
Leadlikeness	Yes
Synthetic accessibility	2.60

Table S23. The ADME properties of ZINC000097971592 predicted by SwissADME webserver.

Molecule 13

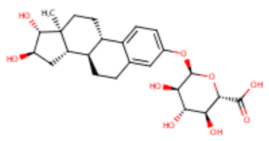

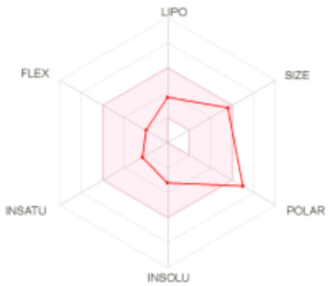

SMILES O[C@@H]1[C@@H](O)[C@@H](Oc2ccc3c(c2)CC[C@@H]2[C@@H]3CC[C@]3([C@H]2C[C@H]([C@@H]3O)O)C)O[C@@H]([C@H]1O)C(=O)O

Physicochemical Properties	
Formula	C24H32O9
Molecular weight	464.51 g/mol
Num. heavy atoms	33
Num. arom. heavy atoms	6
Fraction Csp3	0.71
Num. rotatable bonds	3
Num. H-bond acceptors	9
Num. H-bond donors	6
Molar Refractivity	114.92
TPSA	156.91 Å²

Lipophilicity	
Log $P_{o/w}$ (iLOGP)	1.38
Log $P_{o/w}$ (XLOGP3)	0.89
Log $P_{o/w}$ (WLOGP)	0.15
Log $P_{o/w}$ (MLOGP)	0.25
Log $P_{o/w}$ (SILICOS-IT)	-0.27
Consensus Log $P_{o/w}$	0.48

Water Solubility	
Log *S* (ESOL)	-3.22
Solubility	2.82e-01 mg/ml ; 6.06e-04 mol/l
Class	Soluble
Log *S* (Ali)	-3.77
Solubility	7.89e-02 mg/ml ; 1.70e-04 mol/l
Class	Soluble
Log *S* (SILICOS-IT)	-0.74
Solubility	8.36e+01 mg/ml ; 1.80e-01 mol/l
Class	Soluble

Pharmacokinetics	
GI absorption	Low
BBB permeant	No
P-gp substrate	Yes
CYP1A2 inhibitor	No
CYP2C19 inhibitor	No
CYP2C9 inhibitor	No
CYP2D6 inhibitor	No
CYP3A4 inhibitor	No
Log K_p (skin permeation)	-8.50 cm/s

Druglikeness	
Lipinski	Yes; 1 violation: NHorOH>5
Ghose	Yes
Veber	No; 1 violation: TPSA>140
Egan	No; 1 violation: TPSA>131.6
Muegge	No; 2 violations: TPSA>150, H-don>5
Bioavailability Score	0.11

Medicinal Chemistry	
PAINS	0 alert
Brenk	0 alert
Leadlikeness	No; 1 violation: MW>350
Synthetic accessibility	5.65

Table S24. The ADME properties of ZINC000237948681 predicted by SwissADME webserver.

Molecule 14

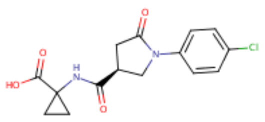

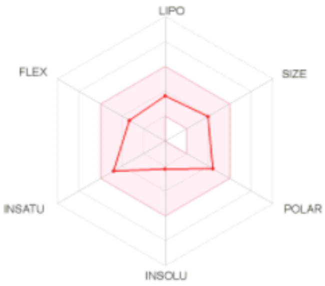

SMILES O=C([C@@H]1CN(C(=O)C1)c1ccc(cc1)Cl)NC1(CC1)C(=O)O

Physicochemical Properties	
Formula	C15H15ClN2O4
Molecular weight	322.74 g/mol
Num. heavy atoms	22
Num. arom. heavy atoms	6
Fraction Csp3	0.40
Num. rotatable bonds	5
Num. H-bond acceptors	4
Num. H-bond donors	2
Molar Refractivity	83.03
TPSA	86.71 Å²

Lipophilicity	
Log $P_{o/w}$ (iLOGP)	1.73
Log $P_{o/w}$ (XLOGP3)	0.84
Log $P_{o/w}$ (WLOGP)	0.98
Log $P_{o/w}$ (MLOGP)	1.31
Log $P_{o/w}$ (SILICOS-IT)	1.64
Consensus Log $P_{o/w}$	1.30

Water Solubility	
Log *S* (ESOL)	-2.24
Solubility	1.85e+00 mg/ml ; 5.73e-03 mol/l
Class	Soluble
Log *S* (Ali)	-2.24
Solubility	1.84e+00 mg/ml ; 5.70e-03 mol/l
Class	Soluble
Log *S* (SILICOS-IT)	-3.29
Solubility	1.66e-01 mg/ml ; 5.15e-04 mol/l
Class	Soluble

Pharmacokinetics	
GI absorption	High
BBB permeant	No
P-gp substrate	Yes
CYP1A2 inhibitor	No
CYP2C19 inhibitor	No
CYP2C9 inhibitor	No
CYP2D6 inhibitor	No
CYP3A4 inhibitor	No
Log K_p (skin permeation)	-7.67 cm/s

Druglikeness	
Lipinski	Yes; 0 violation
Ghose	Yes
Veber	Yes
Egan	Yes
Muegge	Yes
Bioavailability Score	0.56

Medicinal Chemistry	
PAINS	0 alert
Brenk	0 alert
Leadlikeness	Yes
Synthetic accessibility	2.55

Table S25. The ADME properties of ZINC000238950253 predicted by SwissADME webserver.

Molecule 15

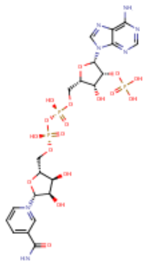

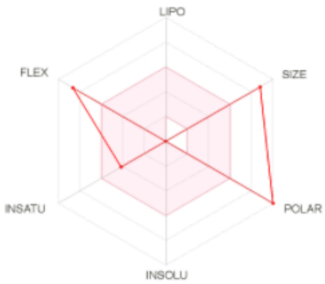

SMILES: O[C@@H]1[C@H](O)[C@H](O[C@H]1[n+]1cccc(c1)C(=O)N)COP(=O)(OP(=O)(OC[C@@H]1O[C@@H]([C@@H]([C@@H]1O)OP(=O)(O)O)n1cnc2c1ncnc2N)O)O

Physicochemical Properties	
Formula	C21H29N7O17P3
Molecular weight	744.41 g/mol
Num. heavy atoms	48
Num. arom. heavy atoms	15
Fraction Csp3	0.48
Num. rotatable bonds	13
Num. H-bond acceptors	20
Num. H-bond donors	9
Molar Refractivity	151.86
TPSA	394.22 Å²

Lipophilicity	
Log $P_{o/w}$ (iLOGP)	-4.57
Log $P_{o/w}$ (XLOGP3)	-7.02
Log $P_{o/w}$ (WLOGP)	-3.54
Log $P_{o/w}$ (MLOGP)	-5.09
Log $P_{o/w}$ (SILICOS-IT)	-7.57
Consensus Log $P_{o/w}$	-5.56

Water Solubility	
Log *S* (ESOL)	0.59
Solubility	2.92e+03 mg/ml ; 3.93e+00 mol/l
Class	Highly soluble
Log *S* (Ali)	-0.55
Solubility	2.12e+02 mg/ml ; 2.85e-01 mol/l
Class	Very soluble
Log *S* (SILICOS-IT)	2.96
Solubility	6.79e+05 mg/ml ; 9.12e+02 mol/l
Class	Soluble

Pharmacokinetics	
GI absorption	Low
BBB permeant	No
P-gp substrate	Yes
CYP1A2 inhibitor	No
CYP2C19 inhibitor	No
CYP2C9 inhibitor	No
CYP2D6 inhibitor	No
CYP3A4 inhibitor	No
Log K_p (skin permeation)	-15.83 cm/s

Druglikeness	
Lipinski	No; 3 violations: MW>500, NorO>10, NHorOH>5
Ghose	No; 4 violations: MW>480, WLOGP<-0.4, MR>130, #atoms>70
Veber	No; 2 violations: Rotors>10, TPSA>140
Egan	No; 1 violation: TPSA>131.6
Muegge	No; 5 violations: MW>600, XLOGP3<-2, TPSA>150, H-acc>10, H-don>5
Bioavailability Score	0.11

Medicinal Chemistry	
PAINS	0 alert
Brenk	2 alerts: phosphor, quaternary_nitrogen_1
Leadlikeness	No; 2 violations: MW>350, Rotors>7
Synthetic accessibility	6.21

Table S26. The ADME properties of ZINC000257306096 predicted by SwissADME webserver.

Molecule 18

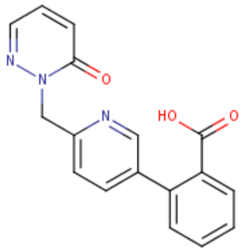

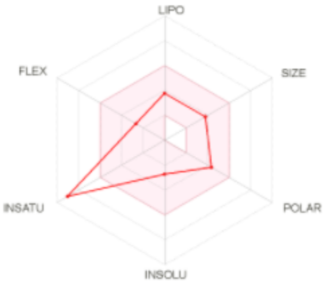

SMILES OC(=O)c1ccccc1c1ccc(nc1)Cn1ncccc1=O

Physicochemical Properties	
Formula	C17H13N3O3
Molecular weight	307.30 g/mol
Num. heavy atoms	23
Num. arom. heavy atoms	18
Fraction Csp3	0.06
Num. rotatable bonds	4
Num. H-bond acceptors	5
Num. H-bond donors	1
Molar Refractivity	84.44
TPSA	85.08 Å²

Lipophilicity	
Log $P_{o/w}$ (iLOGP)	1.77
Log $P_{o/w}$ (XLOGP3)	1.08
Log $P_{o/w}$ (WLOGP)	2.05
Log $P_{o/w}$ (MLOGP)	1.72
Log $P_{o/w}$ (SILICOS-IT)	2.29
Consensus Log $P_{o/w}$	1.78

Water Solubility	
Log *S* (ESOL)	-2.74
Solubility	5.58e-01 mg/ml ; 1.82e-03 mol/l
Class	Soluble
Log *S* (Ali)	-2.46
Solubility	1.07e+00 mg/ml ; 3.48e-03 mol/l
Class	Soluble
Log *S* (SILICOS-IT)	-5.16
Solubility	2.13e-03 mg/ml ; 6.92e-06 mol/l
Class	Moderately soluble

Pharmacokinetics	
GI absorption	High
BBB permeant	No
P-gp substrate	No
CYP1A2 inhibitor	No
CYP2C19 inhibitor	No
CYP2C9 inhibitor	No
CYP2D6 inhibitor	No
CYP3A4 inhibitor	No
Log K_p (skin permeation)	-7.41 cm/s

Druglikeness	
Lipinski	Yes; 0 violation
Ghose	Yes
Veber	Yes
Egan	Yes
Muegge	Yes
Bioavailability Score	0.56

Medicinal Chemistry	
PAINS	0 alert
Brenk	0 alert
Leadlikeness	Yes
Synthetic accessibility	2.70

Table S27. The ADME properties of ZINC000299798705 predicted by SwissADME webserver.

Molecule 19

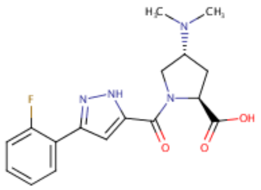

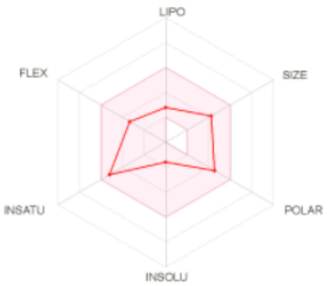

SMILES OC(=O)[C@@H]1C[C@H](CN1C(=O)c1[nH]nc(c1)c1ccccc1F)N(C)C

Physicochemical Properties	
Formula	C17H19FN4O3
Molecular weight	346.36 g/mol
Num. heavy atoms	25
Num. arom. heavy atoms	11
Fraction Csp3	0.35
Num. rotatable bonds	5
Num. H-bond acceptors	6
Num. H-bond donors	2
Molar Refractivity	92.38
TPSA	89.53 Å²

Lipophilicity	
Log $P_{o/w}$ (iLOGP)	1.86
Log $P_{o/w}$ (XLOGP3)	-0.64
Log $P_{o/w}$ (WLOGP)	1.48
Log $P_{o/w}$ (MLOGP)	1.16
Log $P_{o/w}$ (SILICOS-IT)	1.37
Consensus Log $P_{o/w}$	1.05

Water Solubility	
Log *S* (ESOL)	-1.58
Solubility	9.11e+00 mg/ml ; 2.63e-02 mol/l
Class	Very soluble
Log *S* (Ali)	-0.77
Solubility	5.92e+01 mg/ml ; 1.71e-01 mol/l
Class	Very soluble
Log *S* (SILICOS-IT)	-3.41
Solubility	1.34e-01 mg/ml ; 3.86e-04 mol/l
Class	Soluble

Pharmacokinetics	
GI absorption	High
BBB permeant	No
P-gp substrate	Yes
CYP1A2 inhibitor	No
CYP2C19 inhibitor	No
CYP2C9 inhibitor	No
CYP2D6 inhibitor	No
CYP3A4 inhibitor	No
Log K_p (skin permeation)	-8.87 cm/s

Druglikeness	
Lipinski	Yes; 0 violation
Ghose	Yes
Veber	Yes
Egan	Yes
Muegge	Yes
Bioavailability Score	0.55

Medicinal Chemistry	
PAINS	0 alert
Brenk	0 alert
Leadlikeness	Yes
Synthetic accessibility	3.45

Table S28. The ADME properties of ZINC000408592119 predicted by SwissADME webserver.

Molecule 20

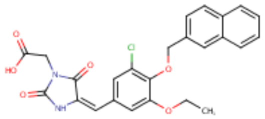

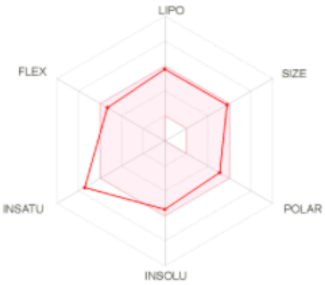

SMILES CCOc1cc(/C=C\2/NC(=O)N(C2=O)CC(=O)O)cc(c1OCc1ccc2c(c1)ccc2)Cl

Physicochemical Properties	
Formula	C25H21ClN2O6
Molecular weight	480.90 g/mol
Num. heavy atoms	34
Num. arom. heavy atoms	16
Fraction Csp3	0.16
Num. rotatable bonds	8
Num. H-bond acceptors	6
Num. H-bond donors	2
Molar Refractivity	134.53
TPSA	105.17 Å²

Lipophilicity	
Log $P_{o/w}$ (iLOGP)	2.87
Log $P_{o/w}$ (XLOGP3)	4.54
Log $P_{o/w}$ (WLOGP)	3.43
Log $P_{o/w}$ (MLOGP)	2.54
Log $P_{o/w}$ (SILICOS-IT)	4.19
Consensus Log $P_{o/w}$	3.51

Water Solubility	
Log *S* (ESOL)	-5.50
Solubility	1.51e-03 mg/ml ; 3.15e-06 mol/l
Class	Moderately soluble
Log *S* (Ali)	-6.47
Solubility	1.63e-04 mg/ml ; 3.38e-07 mol/l
Class	Poorly soluble
Log *S* (SILICOS-IT)	-7.53
Solubility	1.42e-05 mg/ml ; 2.94e-08 mol/l
Class	Poorly soluble

Pharmacokinetics	
GI absorption	High
BBB permeant	No
P-gp substrate	No
CYP1A2 inhibitor	No
CYP2C19 inhibitor	Yes
CYP2C9 inhibitor	Yes
CYP2D6 inhibitor	No
CYP3A4 inhibitor	Yes
Log K_p (skin permeation)	-6.01 cm/s

Druglikeness	
Lipinski	Yes; 0 violation
Ghose	No; 2 violations: MW>480, MR>130
Veber	Yes
Egan	Yes
Muegge	Yes
Bioavailability Score	0.56

Medicinal Chemistry	
PAINS	0 alert
Brenk	2 alerts: hydantoin, michael_acceptor_1
Leadlikeness	No; 3 violations: MW>350, Rotors>7, XLOGP3>3.5
Synthetic accessibility	3.72
